# Amide-controlled, one-pot synthesis of tri-substituted purines generates structural diversity and analogues with trypanocidal activity

**DOI:** 10.1038/srep09139

**Published:** 2015-03-16

**Authors:** Maria J. Pineda de las Infantas y Villatoro, Juan D. Unciti-Broceta, Rafael Contreras-Montoya, Jose A. Garcia-Salcedo, Miguel A. Gallo Mezo, Asier Unciti-Broceta, Juan J. Diaz-Mochon

**Affiliations:** 1 Departamento de Química Farmacéutica y Orgánica. University of Granada, Campus de Cartuja s/n, 18071 Granada, Spain; 2 Pfizer - Universidad de Granada - Junta de Andalucía Centre for Genomics and Oncological Research (GENYO), Parque Tecnológico de Ciencias de la Salud (PTS), Avenida de la Ilustración 114, 18016 Granada, Spain; 3 Unidad de Enfermedades Infecciosas y Microbiología, Complejo Hospitalario de Granada, Instituto de Investigación Biosanitaria de Granada, Dr. Azpitarte, 4, 18012 Granada, Spain; 4 Edinburgh Cancer Research UK Centre, MRC Institute of Genetics and Molecular Medicine, University of Edinburgh, Crewe Road South, Edinburgh EH4 2XR, UK

## Abstract

A novel one-pot synthesis of tri-substituted purines and the discovery of purine analogues with trypanocidal activity are reported. The reaction is initiated by a metal-free oxidative coupling of primary alkoxides and diaminopyrimidines with Schiff base formation and subsequent annulation in the presence of large *N*,*N*-dimethylamides (e.g. *N*,*N*-dimethylpropanamide or larger). This synthetic route is in competition with a reaction previously-reported by our group^1^, allowing the generation of a combinatorial library of tri-substituted purines by the simple modification of the amide and the alkoxide employed. Among the variety of structures generated, two purine analogues displayed trypanocidal activity against the protozoan parasite *Trypanosoma brucei* with IC_50_ < 5 μM, being each of those compounds obtained through each of the synthetic pathways.

Purines are one of the most widely occurring heterocycles in nature^2^. This class of fused [6:5] nitrogen-containing heterocycles are the core structure of nucleobases (e.g. adenine and guanine, essential building blocks of biological structures), and of a wide range of natural compounds with pharmacological properties, including alkaloids (e.g. caffeine and theobromine), cytokines, and natural antibiotics^2^, thus being considered privileged scaffolds for drug development^3^,^4^. Due to their similarity with cell components, purine analogues may act as substrates or inhibitors of enzymes of purine metabolism^5^ or as agonists/antagonists/inhibitors of adenosine receptors^6^ and protein kinases^7^. This is particularly relevant in the development of antiparasitic chemotherapies, since most parasites rely heavily on purine salvage pathways as they cannot synthesize them *de novo*^8^. Purine libraries decorated with different substituents might thus be expected to have a high probability of yielding bioactive compounds and, consequently, purine-derived compounds have been subject to vast exploration in both the heterocyclic and the medicinal chemistry fields^9^,^10^,^11^,^12^. As a result, a number of pharmacologically-active purine analogues have been approved for their clinical application as chemotherapeutic agents (antiviral, antiprotozoal, antifungal and anticancer agents^13^,^14^) and as pharmacodynamic drugs (coronary vasodilator)^15^. Therefore, the development of diversity generating methods to synthesize novel poly-substituted purines potentially represents a rapid source of drug candidates for several therapeutic applications^16^.

Due to their low cost, commercial availability and the range of well-established synthetic protocols existing in the literature, the most common starting material used to generate purine analogues are pyrimidines^17^,^18^,^19^,^20^. Over a decade ago^1^, we reported the one-pot synthesis of poly-substituted purines (**5**) from 4-alkylamino-5-amino-6-chloropyrimidines (**4**), primary alkoxides (**2**) and either *N,N*-dimethylformamide or *N,N*-dimethylacetamide (**1**) (Fig. 1). Alkoxide anions were formed in excess by reaction of sodium hydride and the corresponding primary alcohols. It was proposed that reactive species (alkoxyiminium **3**) was generated in situ by reaction between the amide **1** and the alkoxides **2**, leading to poly-substituted purine analogues with either H or methyl at C8 depending on the amide employed. This mechanism was supported by the formation of the expected purine analogues using dimethylformamide dimethylacetal (orthoamide). S_N_Ar of the chloro atom at C6 by various alkoxides and the R^3^ group of the pyrimidine was used to implement structural diversity, thus allowing the straightforward generation of a 12-member library of purine analogues.

## Results and Discussion

**Figure 1:**
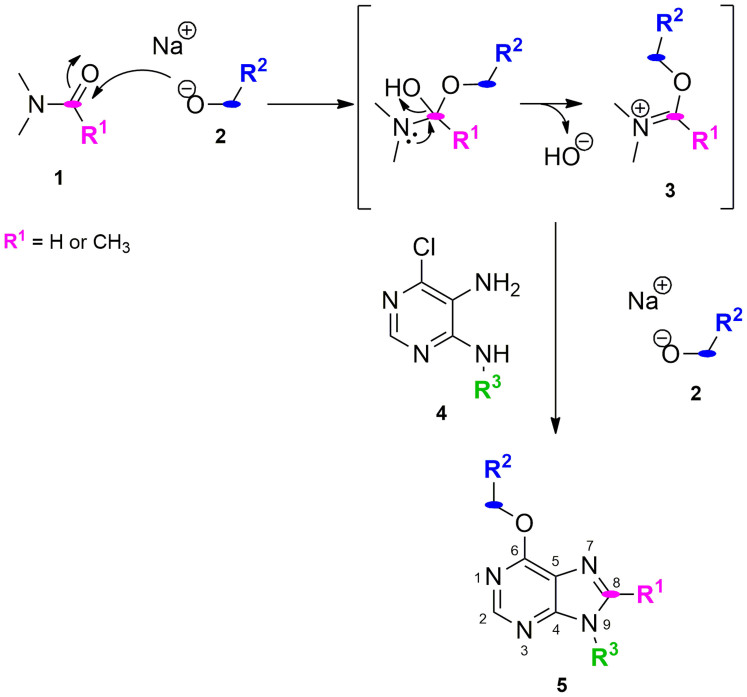
Plausible mechanism of the one-pot synthesis of poly-substituted purines previously developed by our group^1^.

**Figure 2:**
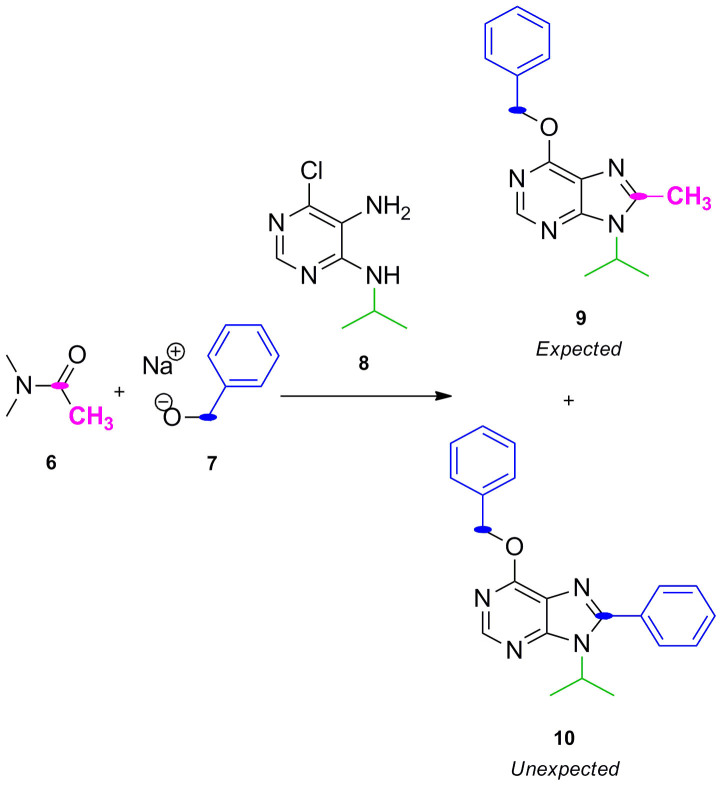
Expected and unexpected products obtained by reaction of *N,N*-dimethylacetamide, benzyl alkoxide (benzyl alcohol + sodium hydride), and 5-amino-6-chloro-4-isopropylamino-pyrimidine.

Motivated by the potential pharmacological properties of these privileged structures, we revisited this methodology to create a new library of poly-substituted purines using a range of different pyrimidines, *N,N*-dimethylamides and alcohols. Interestingly, following the same procedure, purines **9** and **10** were isolated after reaction of *N,N*-dimethylacetamide **6**, benzyl alkoxide **7** (in situ generated by reaction of sodium hydride and benzyl alcohol) and 5-amino-6-chloro-4-isopropylaminopyrimidine **8** (Fig. 2). The unexpected formation of purine analogue **10** suggested that the substituent at C8 originated from the benzyl alkoxide **7** rather than from the amide **6**. During the investigation of the possible mechanism involved in the synthesis of purine **10**, the use of dialkylacetals (orthoamide) as alkoxyiminium ion synthons to obtain quinazolines from anthranilamides was reported^21^, which endorsed the mechanism proposed in Fig. 1 but left the formation of purine **10** unexplained. Together with the fact that alkoxyiminium ions are also considered a source of very reactive species capable of doing electrophilic *N*-alkylation^22^, the intriguing generation of purine **10** and the potential versatility of the process prompted us to both investigate and exploit this synthetic method further.

**Figure 3:**
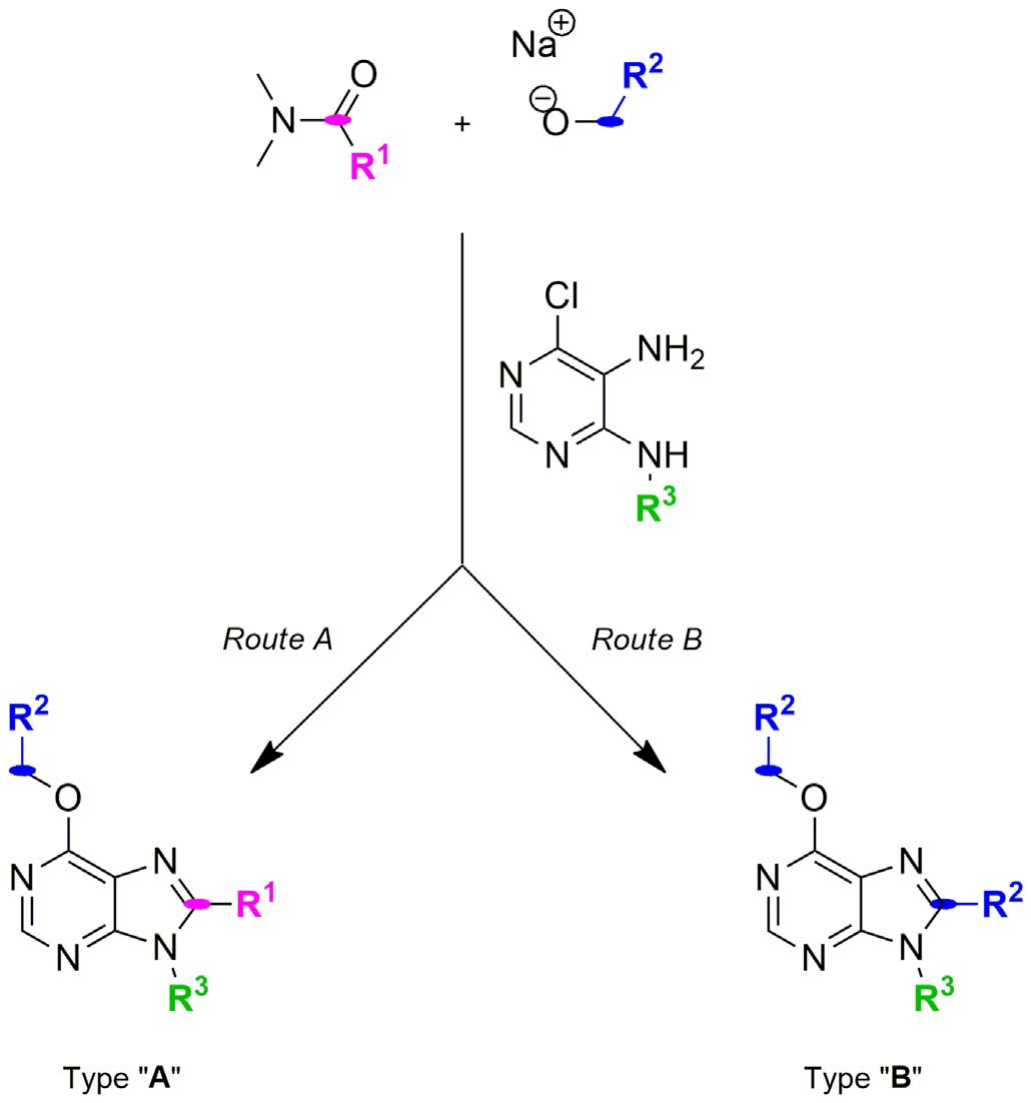
One-pot synthesis of poly-substituted purines using sodium hydride and different *N,N*-dimethylamides, primary alcohols and 4-alkylamino-5-amino-6-chloropyrimidines.

A set of reactions were planned to generate new purine analogues and, in turn, finding out key aspects of the mechanism through which purine **10** was formed. Reactions among 4-alkylamino-5-amino-6-chloropyrimidines, *N,N*-dimethylamides and different alcohol sources were carried out. Following purification and characterization, products were classified into two categories: (i) type A purines, containing a C8 substituent (**R^1^**) derived from the amide used, and (ii) type B purines, with the C8 substituent (**R^2^**) revealing an alternate mechanism where the cyclo-condensation agent appeared to originate from the alcohol/alkoxide species (see Fig. 3).

Table 1 summarizes the reactions performed and the major purine analogue isolated (see Fig. 3 for chemical structures of both type of compounds). It was clearly observed that steric features of the reactants played a key role in determining towards which route the reaction occurs. Large amides such as *N,N*-dimethylisopropionamide and *N,N*-dimethylbenzamide (**R^1^**_ = _^i^Pr or Ph) gave rise to type B purines with each of the alcohols used (entries B5–9). Similarly, the use of *N,N*-dimethylpropionamide (**R^1^**_ = _ Et) with benzyl or p-methoxybenzyl alcohols (bulky **R^2^**) led to type B analogues (entries B1-4). Interestingly, type A purines were the major products when *N,N*-dimethylpropionamide (**R^1^**_ = _ Et) was used together with a small alcohol such as ethanol (entry A2 and 3), and when a smaller amide such as *N,N*-dimethylacetamide were used (entry A1). Minor traces of type B (<2.5%) analogues were also isolated from these reactions, showing a competition between both routes. In accordance with previous observation^1^, reactions developed with *N,N*-dimethylformamide exclusively produced type A purine analogues regardless the alcohol employed (entries A4 and 5).

This synthetic study demonstrates competition between two different synthetic pathways, with the kinetics of intermediate formation for route A and B driving the final outcome. When the amide employed is small (e.g. DMF), reaction between amides and alkoxides will form reactive alkoxyiminium (**3**) species that will lead to type A analogues. On the contrary, route B governs the synthetic process in the presence of larger *N,N*-dimethylamides, suggesting that either the resulting alkoxyiminium (**3**) species is too stable/sterically hindered to be attacked by the diaminopyrimidines or its own formation is hindered by the size of the amide.

**Figure 4:**
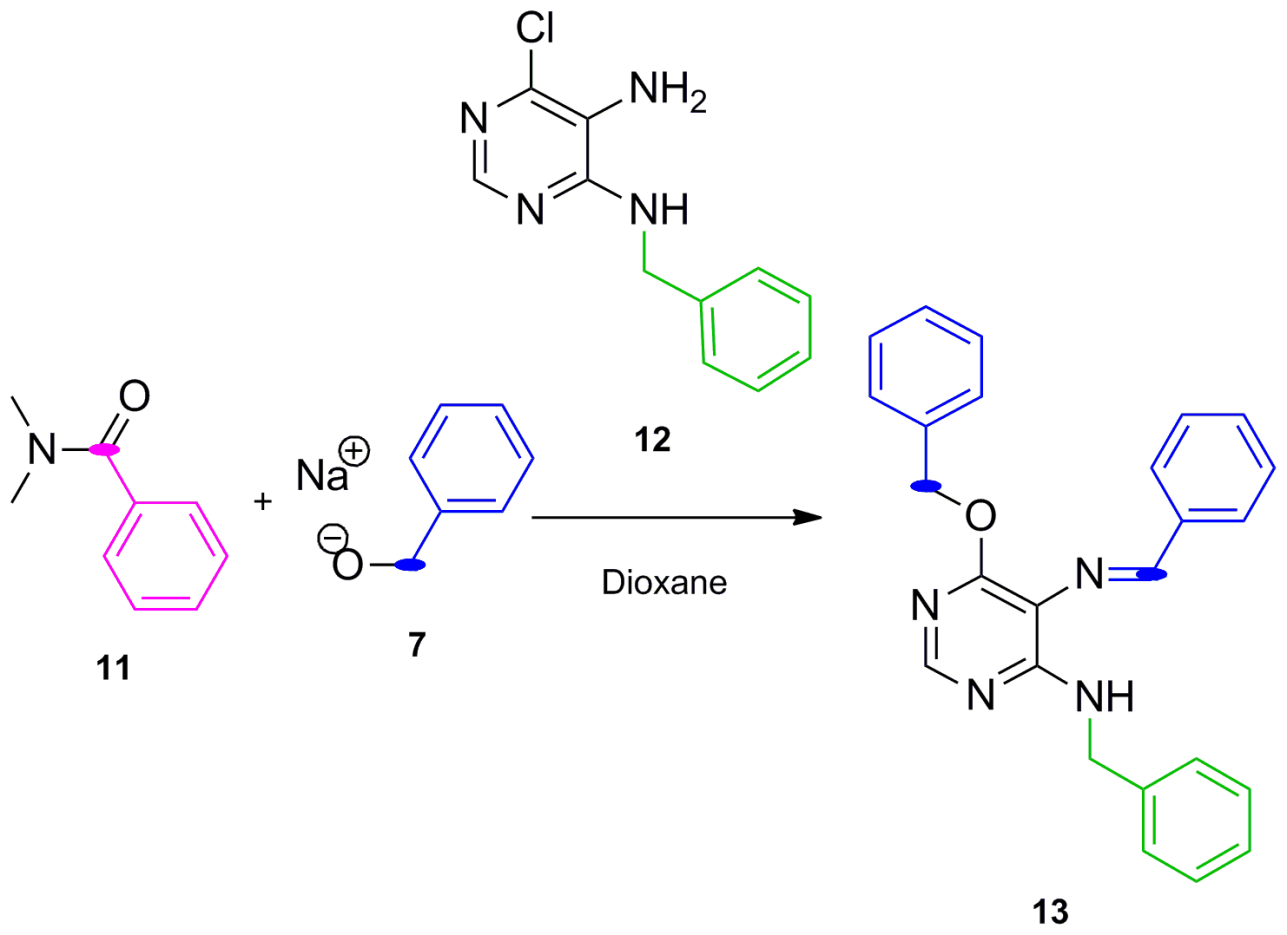
Synthesis of Schiff base 13 by sterically-hindered starting materials.

As mentioned before, examination of the type B purines synthesized clearly indicated that their C8 substituents were originated from the primary alcohols/alkoxides (R^2^). Since a carbon electrophilic center directly derived from an alcohol could only mediate amine alkylation, any synthetic pathway leading to route B would necessarily involve an oxidation step (e.g. of the alcohol/alkoxide into the corresponding aldehyde or the *N*-alkylated pyrimidine to the Schiff base), which would in turn act as the cyclo-condensation agent. We anticipated that introducing steric impediments in the starting materials could allow the trapping of the imine intermediate before the cyclization is completed, thus proving the transitional formation of Schiff base. To this end, *N,N*-dimethylbenzamide **11**, benzyl alkoxide **7** and 5-amino-4-benzylamino-6-chloropyrimidine **12** were handpicked and subjected to the same reaction conditions. As a result (Fig. 4), Schiff base intermediate **13** was isolated without detectable formation of the purine analogue, corroborating the involvement of an oxidative coupling of benzyl alcohol and 5-amino-4-benzylamino-6-chloropyrimidine **12** as the essential initiating step required to generate type B purine analogues.

**Figure 5:**
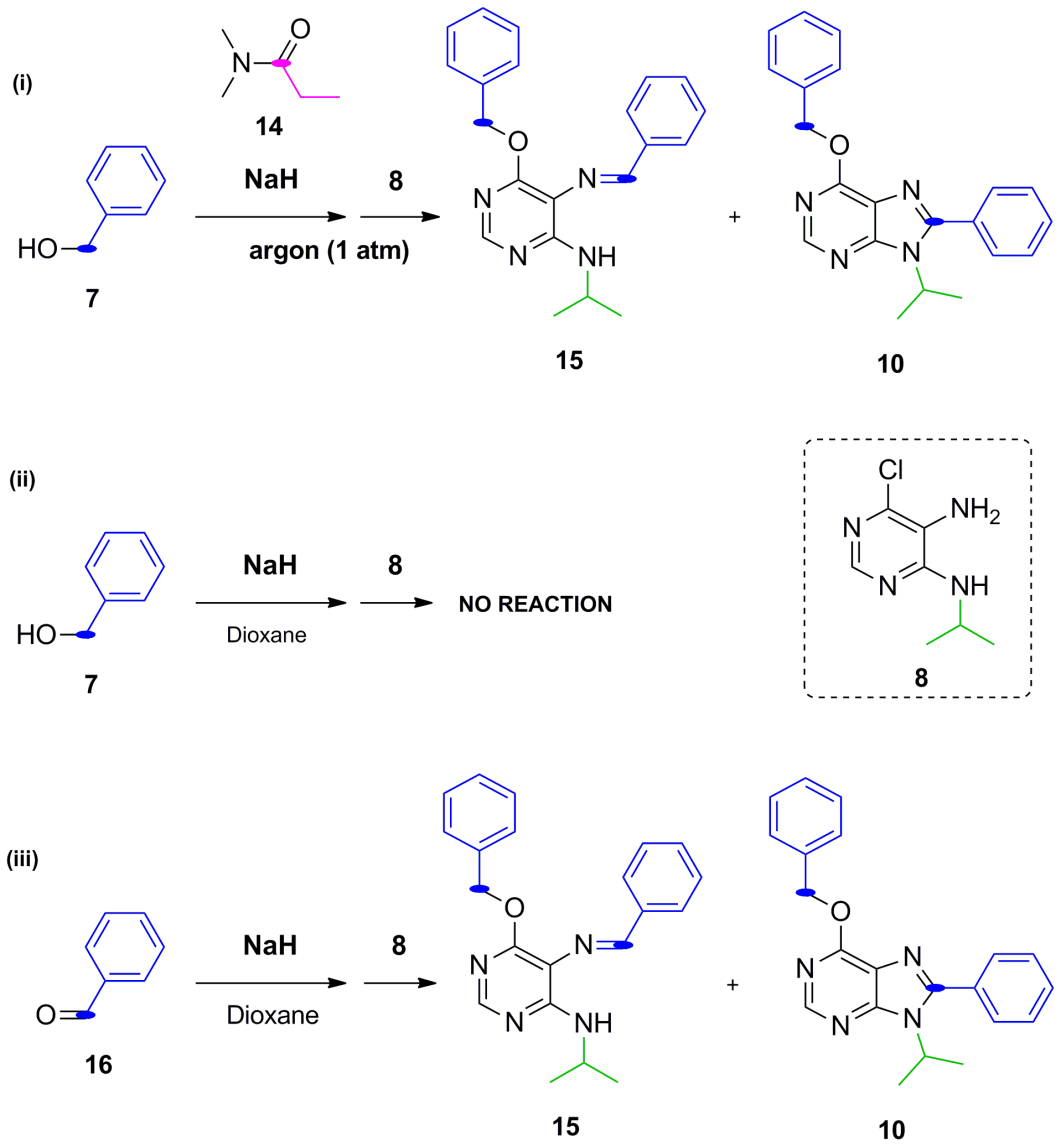
Study of the significance of different chemical reactants on the generation of type B analogues.

Since reagents with higher oxidation states than aldehydes are typically required to cyclo-condense and give rise to an aromatic heterocycle^23^,^24^,^25^,^26^, a second oxidative step will need to occur for driving the transformation of an unstable dihydropurine intermediate^27^ (resulting from the intramolecular attack of the alkylamino group in C4 to the imine) into a purine system and thus complete synthetic route B. Therefore, overall, synthesis of type B purine analogues would require two oxidative steps for completion: first, oxidative coupling of an alcohol and an exocyclic amine group to generate a Schiff base (able to transitionally generate a dihydropurine derivative); and second, the oxidative generation of a purine system from an unstable 7,8-dihydropurine intermediate^28^. Literature provide different examples of each of this oxidative steps independently but, to our knowledge, this is the first time that both are reported concomitantly to obtain substituted purines. For instance, a recent study has reported the use of NaH/air as the oxidant source to obtain aldehyde from alcohols without the need of using metals as catalyst^29^. Within this line of research, three different manuscripts have reported metal-free oxidative coupling of alcohols and amines, mediated by strong basic conditions, to give rise to either imine or alkylated amines.^30^,^31^,^32^. The synthesis of purine rings from diaminopyrimidines using both aromatic and aliphatic aldehydes and an iron catalyst has also been previously described^33^. Furthermore, evidences of metal-free oxygen-promoted oxidation of electron-poor heterocycles in strong basic conditions have been shown by us^34^ and others^35^. With these precedents in mind, a set of reactions using reagents with large substituents (so that Route B pathway was to be favored) were designed to shed some light on the oxidative aspects of route B mechanism. Three reactions were performed in parallel with NaH and pyrimidine **8** as starting material and: (i) benzyl alcohol **7** and *N,N*-dimethylpropionamide **14** in argon atmosphere (oxygen-free environment), (ii) amide-free reaction with benzyl alcohol **7** and (iii) amide-free reaction with benzaldehyde **16** instead of benzyl alcohol (Fig. 5).

In the absence of oxygen, reaction (i) yielded a mixture of the purine end-product **10** and Schiff base intermediate **15**. This finding was, in principle, contradictory to previous report as metal-free oxidative couplings are achieved under aerobic conditions. However, this could be explained by presence of oxygen traces coming from the cylinder, an observation also found by Adimurthy^30^ The presence of Schiff base intermediate **15** shows the importance of oxygen as a promoter of the second oxidation step. When the process was carried out in the absence of the amide (panel (ii) of Fig. 5) and using dioxane as solvent the reaction did not take place and the starting material **8** was recovered. To shed light into the role of the amide in the reaction, the same reaction was repeated with toluene instead of dioxane as Adimurthy^30^ observed that oxidative iminations are promoted in toluene but not in dioxane. In agreement with Adimurthy's observations, Schiff base intermediate **15** was obtained under these conditions. Interestingly, purine **10** was not formed, highlighting the assisting role of amides in the annulation step but not for the oxidative imination.

We then investigated if aldehyde intermediates, which would appear to be the oxidative intermediate needed for Schiff base formation in the first step, could be identified. Reaction of benzaldehyde **16**, NaH and pyrimidine **8** (condition (iii)) gave rise to purine **10** and Schiff base **15**, demonstrating that the direct use of the aldehyde^36^ can lead to the end-products without the need of the amide, meaning that at this point the aldehyde intermediate hypothesis was still credible. In this case and remarkably, while benzyl alcohol **7** was not present in the reaction mixture, C6-benzoxy products were obtained, suggesting a partial reduction of benzaldehyde into benzyl alkoxide probably mediated by NaH^37^. We then carried out a set of reactions to try to capture the aldehyde intermediate, which might be transitionally created in-situ. 2,4-dinitrophenylhydrazine was chosen as indicator in substitution of the pyrimidine, aiming that upon reaction with the expected aldehyde intermediate would create a hydrazone with a colour change. Benzyl alcohol **7**, *N,N-*dimethylpropanamide **14** and NaH were used as the other reagents under the same conditions mentioned above. However, only 2,4-dinitrophenylhydrazine decomposition was observed, likely due to the strong basic conditions of the reaction. Reactions using just benzyl alcohol **7**, *N,N*-dimethylpropanamide **14** and NaH, in the absence of any amino-containing starting material, were then carried to try to identify benzaldehyde by TLC. However, no traces of benzaldehyde **16** were detected. Hence, we could not identify aldehyde species under any of these conditions and, even though Adimurthy reported the presence of 1%-2% of in situ generated catalytic aldehyde as the responsible species to trigger these reactions, we cannot confirm that, in our case, the oxidative imination takes place through aldehyde intermediates.

**Figure 6:**
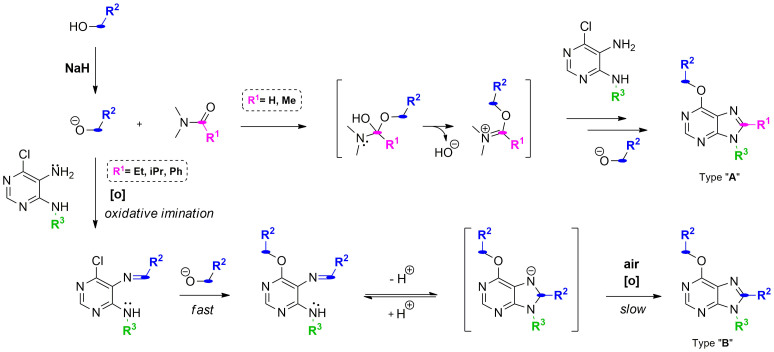
Proposed mechanisms of action for routes A and B. In accordance to the literature^31^, the imine intermediate can give rise, in a reversible manner, to dihydropurines. The tandem synthetic process would end upon irreversible oxidation into purines.

To summarize, the following features of the process have been gathered from these studies are:

- There is competition between alkoxyiminium ion formation (created by reacting *N,N-*dimethylamides and alkoxide) and metal-free oxidative imination (reaction between amine and alcohols assisted by strong basic conditions in open systems). When large *N,N*-dimethylamides (R^1^_ = _ Et, iPr, Ph) are used, the formation of alkoxyiminium ions is disfavoured and therefore purine formation via oxidative imination governs. In case of using smaller *N,N*-dimethylamides (R^1^_ = _ H, Me), alkoxyiminium ion formation is formed faster and purines type A are obtained (Fig. 6).
- There is no evidence of neither NaH to be directly involved in the oxidation steps nor aldehyde to be the intermediate required to obtain the Schiff base.
- Final products from reactions (i) and (iii) and the starting material recovered from reaction (ii) demonstrate that S_N_Ar of the chloro atom at C6 by the alkoxide takes place upon formation of the Schiff base in a rapid manner.
- While the second oxidation step (purine formation) does not require the presence of the amide, the type of solvent influences whether the annulation step does or does not take place. Incomplete reactions (i) and (iii) indicate that oxygen and strong basic conditions are promoters of the purine ring formation.

**Figure 7:**
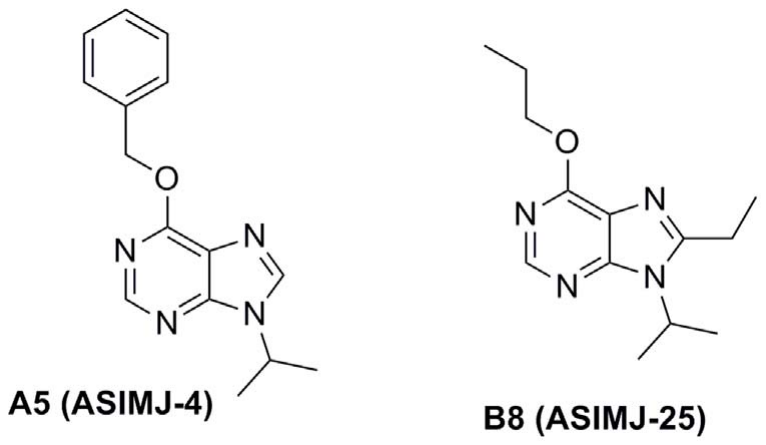
Chemical structures of compounds A5 (ASIMJ-4) and B8 (ASIMJ-25), the most active of the library against the protozoan parasite *Trypanosoma brucei.*

The second part of the work was to investigate whether the chemical diversity produced through the described synthetic methodology had resulted in the generation of biologically active purines. Bearing in mind that purine salvage pathways have been proposed as an interesting therapeutic target for pathogenic protozoa^8^,^38^ we decided to screen the antiparasitic properties of these novel compounds against *Trypanosoma brucei*, the causative agent of African trypanosomiasis, also known as sleeping sickness^39^. Therefore, a trypanotoxicity assay using 11 different concentrations of each of the library members was performed against bloodstream forms of *T. brucei*. Half-inhibitory concentrations (IC_50_) were determined for each of the compounds (Table 1). The screening highlighted the trypanocidal properties of two derivatives: compound B8 (ASIMJ-25), with an IC_50_ of 4.8 ± 0.2 μM mean ± SEM), and the purine analogue A5 (ASIMJ-4) (Fig. 7), which was the most active antiparasite compound of the series with an IC_50_ of 1 ± 0.1 μM (mean ± SEM). Interestingly, these active purines were generated through each of the synthetic routes, and the only common feature was the isopropyl group at the position 9 of the purine.

**Figure 8:**
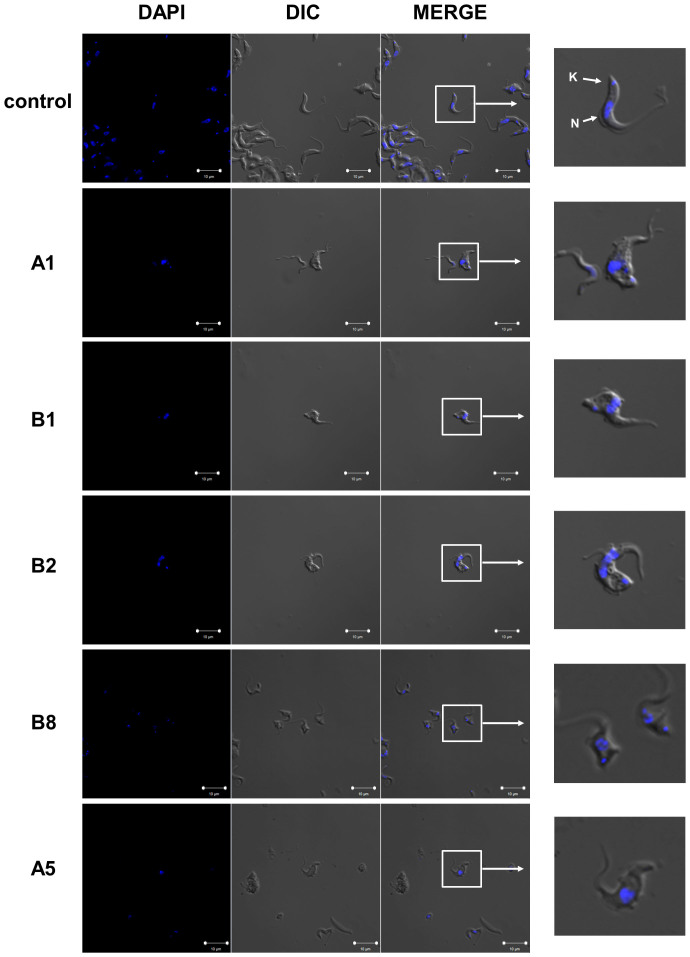
Confocal fluorescence and optical microscopy DIC of bloodstream trypanosomes incubated for 24 h with IC_50_ concentrations of A1 (AIMJ-5), A5 (ASIMJ-4), B1 (ASIMJ-6), B2 (ASIMJ-27), B8 (ASIMJ-25) and controls. DAPI channel stain nucleus (N) and kinetoplast (K).

This inhibitory effect on trypanosomes growth was further analyzed in compounds with the different IC_50_ values, including the two most active compounds of the library, by optical and fluorescence microscopy after DAPI staining. As expected, the numbers of parasites on wells incubated with the selected inhibitors A1 (ASIMJ-5), A5 (ASIMJ-4), B1 (ASIMJ-6), B2 (ASIMJ-27) and B8 (ASIMJ-25), using their IC_50_ concentration values, were much lower than on control wells. Moreover, all of these compounds induced profound morphological changes in the appearance of cells, with enlargement of the flagellar pocket and multiple nuclei, which is a typical phenotype of dying trypanosomes (Fig. 8)^40^,^41^.

## Conclusions

In conclusion, a 14-member library of poly-substituted purines were synthesized from 4-alkylamino-5-amino-6-chloropyrimidines using primary alcohols, sodium hydride and *N,N*-dimethylalkyl/arylamides. Competition between the formation of reactive alkoxyiminium ions and the oxidation of alcohols defined which products were obtained. In the absence of steric impediments, purine analogues were synthesized via reaction of alkoxyiminium ions and diaminopyrimidines. On the contrary, in the presence of larger *N,N*-dimethylamides, a more complex synthetic route dominated, giving rise to an unexpected set of purine analogues. The tandem reaction sequence was initiated by a metal-free oxidative coupling of primary alkoxide and exocyclic amino group at C4 of the pyrimidine ring, obtaining a Schiff base intermediate. This intermediate underwent an intramolecular cyclocondensation with the amino group at C5 driven by an oxygen-promoted alkaline oxidation. Two Schiff base intermediates were isolated and could be obtained in higher yields by increasing the size of the substituents of the amides and of the amino group found at C5 of the pyrimidine. The role of large *N*,*N*-dimethylamides for the formation of Schiff base under these conditions seems to be not crucial as toluene (but not dioxane) also enabled the oxidative imination. However, *N,N*-dimethylamides promoted the second oxidation step to obtain purine rings whereas toluene did not, demonstrating the relevant role of the solvent in the reaction cascade. One aspect that is still inconclusive is which alcohol-derived species carry out the oxidative couplings with amino groups. In this study we could not confirm that the oxidative imination happened via an aldehyde intermediate.

To the best of our knowledge, this tandem synthetic process has been described herein for the first time. From a synthetic/medicinal point of view, and even if the reaction yields are low to moderate, the interest of the present methodology relies on its versatility, since it allows to generate a range of different tri-substituted purine analogues by the simple selection of readily available starting materials.

Finally, we have shown the capacity of some of these novel purine derivatives to reduce trypanosome viability by inducing an enlarged flagellar pocket phenotype with one compound, A5 (ASIMJ-4), presenting an IC_50_ value of 1 μM. Given the relatively small molecular weight of this compound (MW = 263 Da), compound A5(ASIMJ-4) could be considered a fragment with unusually high phenotypic activity. These interesting results underscore the importance of developing short and versatile synthetic pathways capable of generating structural diversity in order to produce novel purine libraries, in rapid parallel fashions, with pharmacological/therapeutic interest.

## Experimental Section

### General Experimental

Reaction courses and products mixtures where routinely monitored by TLC on silica gel Merck 60–200 mesh silica gel. Melting points were determined on a Stuart Scientific SMP3 apparatus and are uncorrected. ^1^H-NMR spectra were obtained in CDCl_3_, CD_3_OD solutions on a Varian Inova Unity (300 MHz) and Varian Direct Drive (400 MHz and 500 MHz). Chemical shifts (δ) are given in ppm upfield from tetramethylsilane. ^13^C-NMR spectra were obtained in CDCl_3_, CD_3_OD solutions on a Varian Direct Drive (125 MHz). All products reported showed ^1^H-NMR and ^13^C-NMR spectra in agreement with the assigned structures. Mass spectra were obtained by electrospray (ESY) with a LCT Premier XE Micromass Instrument (High resolution mass spectrometry).

#### General procedure for the preparation of compounds B1-9 (Route B)

NaH (50%) in mineral oil (3.7 mmol, 10 equiv.) was added to a mixture cooled at 0°C constituted of the corresponding alcohol (3.7 mmol, 10 equiv.) and *N,N*-dimethylamide (18.5 mmol, 50 equiv.). In case of using *N*,*N*-dimethylbenzamide, 1,4-dioxane (2 mL) was used as solvent. The solution was stirred at room temperature for 30 min and then at 90°C for another 30 min. After this time, substituted pyrimidines (0.37 mmol, 1 equiv., 5-amino-6-chloro-4-isopropylaminopyrimidine **8** or 5-amino-4-*tert*-butylamino-6-chloropyrimidine, which prepared as described elsewhere^1^) were dissolved either in the same amide (18.5 mmol, 50 equiv.) or in 1,4-dioxane (2 mL, in the case of *N*,*N*-dimethylbenzamide) and added dropwise to the mixture, which was then heated for 24 h at 90°C. The reaction mixture was brought to pH 7 with an aq. saturated NH_4_Cl solution, and then extracted with CH_2_Cl_2_ (3 × 15 mL). The combined organic extracts were dried over Na_2_SO_4,_ filtered and the organic solvent evaporated. Crudes were purified by flash chromatography on silica gel using ethyl acetate/petroleum ether as mobile phases.

#### 6-Benzyloxy-9-isopropyl-8-phenyl-9H-purine (B1)ASIMJ-6

White solid, mp 111–112°C; yield 40%. δ_H_ (300.20 MHz, CDCl_3_): 8.57 (1H, s, N*CH*N), 7.70–7.38 (10H, m, *Ph,* CH_2_*Ph*), 5.72 (2H, s, OC*H_2_*Ph), 4.80 (1H, m, *CH*(CH_3_)_2_), 1.76 (6H, d, J = 6 Hz, NCH(C*H*_3_)_2_). δ_C_ (125.68 MHz, CDCl_3_): 160.54, 153.14, 151.01, 136.66, 130.40, 129.80, 128.95, 128.76, 128.61, 128.27, 128.05, 122.14, 68.42, 50.04, 21.51. ES + HRMS: Calculated M + H = 345.1715. C_21_H_21_N_4_O. Obtained: 345.1716.

#### 9-tert-butyl-6-(benzyloxy)-8-phenyl-9H-purine (B2) ASIMJ-27

White solid, mp: 113–114°C; yield 45%. δ_H_ (300.20 MHz, CDCl_3_): 8.59 (1H, s, N*CH*N), 7.58–7.34 (10H, m, *Ph,* CH_2_*Ph*), 5.69 (2H, s, OC*H_2_*Ph), 1.70 (9H, s, NC(C*H*_3_)_3_). δ_C_ (125.68 MHz, CDCl_3_): 160.62, 154.52, 153.37, 150.46, 136.58, 134.97, 130.11, 129.72, 128.85, 128.59, 128.27, 128.09, 121.76, 68.43, 61.06, 31.15. ES + HRMS: Calculated M + H = 359.1872. C_22_H_23_N_4_O. Obtained: 359.1880.

#### 6-(3-methoxybenzyloxy)-9-isopropyl-8-(3-methoxyphenyl)-9H-purine (B3)

White viscous oil; yield 28%. δ_H_ (499.79 MHz, CDCl_3_): 8.53 (1H, s, N*CH*N), 7.44–6.85 (8H, m, *Ph,* CH_2_*Ph*), 5.66 (2H, s, OC*H_2_*Ph), 4.79 (1H, m, C*H*(CH_3_)_2_), 3.87 (3H, s, OCH_2_PhOC*H_3_*), 3.80 (3H, s, PhOC*H_3_*), 1.72 (6H, d, NCH(C*H*_3_)_2_). δ_C_ (125.68 MHz, CDCl_3_): 160.27, 159.64, 153.59, 152.75, 150.76, 137.94, 131.25, 129.41, 121.63, 120.78, 116.25, 114.92, 113.88, 113.70, 68.03, 55.45, 55.26, 49.81, 21.26. ES + HRMS: Calculated M + H = 405.1927. C_23_H_25_N_4_O_3_. Obtained: 405.1930.

#### 6-(3-methoxybenzyloxy)-9-tert-butyl-8-(3-methoxyphenyl)-9H-purine (B4)

Yellow viscous oil; yield 22%. δ_H_ (400.57 MHz, CDCl_3_): 8.55 (1H, s, N*CH*N), 7.48–6.83 (8H, m, *Ph,* CH_2_*Ph*), 5.63 (2H, s, OC*H_2_*Ph), 3.83 (3H, s, OCH_2_PhOC*H_3_*), 3.79 (3H, s, PhOC*H_3_*), 1.68 (9H, s, NC(C*H*_3_)_3_). δ_C_ (125.68 MHz, CDCl_3_): 160.29, 159.61, 159.02, 154.21, 152.87, 150.23, 137.83, 129.38, 128.92, 122.49, 120.86, 119.08, 115.41, 113.91, 113.29, 112.24, 68.06, 55.40, 55.27, 30.76. ES + HRMS: Calculated M + H = 419.2083. C_24_H_27_N_4_O_3_. Obtained: 419.2086.

#### 9-Isopropyl-6-methoxy-9H-purine (B5)

White viscous oil; yield 10%. δ_H_ (400.57 MHz, CDCl_3_): 8.53 (1H, s, N*CH*N), 7.98 (1H, s, NH*CH*N^i^Pr), 4.89 (1H, m, *CH*(CH_3_)_2_), 4.18 (3H, s, OC*H_3_*), 1.63 (6H, d, J = 8 Hz, NCH(C*H*_3_)_2_). δ_C_(125.68 MHz, CDCl_3_): 161.19, 152.25, 151.86, 139.89, 122.07, 54.26, 47.63, 22.78. ES + HRMS: Calculated M + H: 193.1089. C_9_H_13_N_4_O. Obtained: 193.1087.

#### 6-Ethoxy-9-isopropyl-8-methyl-9H-purine (with N,N-dimethylisopropionamide) (B6)

Yellow viscous oil; yield 10%. δ_H_(499.79 MHz, CDCl_3_): 8.44 (1H, s, N*CH*N), 4.73 (1H, m, *CH*(CH_3_)_2_), 4.62 (2H, q, J = 10 Hz, OC*H_2_*CH_3_), 2.64 (3H, s, *CH_3_*), 1.68 (6H, d, J = 5 Hz, NCH(C*H*_3_)_2_), 1.50 (3H, t, J = 10 Hz, OCH_2_C*H_3_*). δ_C_(125.68 MHz, CDCl_3_): 159.74, 152.88, 151.75, 150.53, 120.71, 62.67, 48.31, 21.24, 15.22, 14.58. ES + HRMS: Calculated M + H = 221.1402. C_11_H_17_N_4_O. Obtained: 221.1401.

#### 6-Ethoxy-9-isopropyl-8-methyl-9H-purine (with N,N-dimethylbenzamide) (B7)

Yellow viscous oil; yield 20%. δ_H_(499.79 MHz, CDCl_3_): 8.44 (1H, s, N*CH*N), 4.73 (1H, m, *CH*(CH_3_)_2_), 4.62 (2H, q, J = 10 Hz, OC*H_2_*CH_3_), 2.64 (3H, s, *CH_3_*), 1.68 (6H, d, J = 5 Hz, NCH(C*H*_3_)_2_), 1.51 (3H, t, J = 10 Hz, OCH_2_C*H_3_*). δ_C_(125.68 MHz, CDCl_3_): 159.74, 152.88, 151.75, 150.53, 120.71, 62.67, 48.31, 21.24, 15.22, 14.58. ES + HRMS: Calculated M + H = 221.1402. C_11_H_17_N_4_O. Obtained: 221.1396.

#### 8-Ethyl-9-isopropyl-6-propoxy-9H-purine (B8)ASIMJ-25

Yellow viscous oil; yield 27% δ_H_ (300.20 MHz, CDCl_3_): 8.43 (1H, s, N*CH*N), 4.69 (1H, m, *CH*(CH_3_)_2_), 4.52 (2H, t, J = 9 Hz, OC*H_2_*CH_2_CH_3_), 2.94 (2H, q, J = 6 *CH_2_*CH_3_), 1.92 (2H, m, OCH_2_C*H_2_*CH_3_), 1.70 (6H, d, J = 10 Hz, NCH(C*H*_3_)_2_), 1.43 (3H, t, J = 6 Hz, CH_2_C*H*_3_), 1.05 (3H, t, J = 9, OCH*_2_*CH_2_C*H*_3_). δ_C_(125.68 MHz, CDCl_3_): 160.28, 155.28, 150.65, 150.31, 124.41, 68.65, 48.57, 21.24, 23.13, 22.49, 21.51, 12.49, 10.66. ES + HRMS: Calculated M + H = 249.1715. C_13_H_21_N_4_O. Obtained: 249.1721.

#### 6-Isobutoxy-8,9-diisopropyl-9H-purine (B9)

White viscous oil; yield 22%. δ_H_ (300.20 MHz, CDCl_3_): 8.44 (1H, s, N*CH*N), 4.74 (1H, m, N*CH*(CH_3_)_2_), 4.36 (2H, d, OC*H_2_*Pr^i^, J = 9 Hz), 3.28 (1H, m, C*CH*(CH_3_)_2_), 2.29 (1H, m, CH_2_*CH*(CH_3_)_2_), 1.72 (6H, d, J = 9 Hz, NCH(C*H*_3_)_2_), 1.47 (6H, d, J = 9 Hz, CCH(C*H*_3_)_2_), 1.06 (6H, d, J = 9 Hz, CH_2_CH(C*H*_3_)_2_). ES + HRMS: Calculated M + H = 277.2028. C_15_H_25_N_4_O. Obtained: 277.2036.

#### General procedure for the preparation of compounds A1-5 (Route A)

NaH (50%) in mineral oil (3.7 mmol, 10 equiv.) was added to a mixture cooled at 0°C constituted of the corresponding alcohol (3.7 mmol, 10 equiv.) and *N,N*-dimethylamide (18.5 mmol, 50 equiv.). The solution was stirred at room temperature for 30 min and then at 90°C for another 30 min. After this time, substituted pyrimidines (0.37 mmol, 1 equiv.) were dissolved in the amide (18.5 mmol, 50 equiv.) and added dropwise to the mixture which was then heated for 24 h at 90°C. The reaction mixture was brought to pH 7 with an aq. saturated NH_4_Cl solution, and then extracted with CH_2_Cl_2_ (3 × 15 mL). The combined organic extracts were dried over Na_2_SO_4,_ filtered and the organic solvent evaporated. Crudes were purified by flash chromatography on silica gel using ethyl acetate/petroleum ether as mobile phases.

#### 6-(Benzyloxy)-9-isopropyl-8-methyl-9H-purine (A1)ASIMJ-5

White solid, mp: 84-86°C; yield 16%. δ_H_ (499.79 MHz, CDCl_3_): 8.47 (1H, s, N*CH*N), 7.54–7.30 (5H, m, Ph), 5.65 (2H, s, OC*H_2_*Ph), 4.75 (1H, m, *CH*(CH_3_)_2_), 2.65 (3H, s, C*H_3_*), 1.69 (6H, d, J = 10 Hz, NCH(C*H*_3_)_2_). δ_C_ (125.68 MHz, CDCl_3_): 159.41, 153.57, 150.69, 150.41, 136.42, 128.43 128.35, 127.98, 120.73, 68.06, 48.35, 21.23, 15.22. ES + HRMS: Calculated M + H = 283.1559. C_16_H_19_N_4_O. Obtained: 283.1558.

#### 6-Ethoxy-8-ethyl-9-isopropyl-9H-purine (A2)

Yellow viscous oil; yield 29%. δ_H_(300.20 MHz, CDCl_3_): 8.47 (1H, s, N*CH*N), 4.72 (1H, m, *CH*(CH_3_)_2_), 4.67 (2H, q, J = 6 Hz, OC*H_2_*CH_3_), 2.96 (2H, q, J = 6, C*H_2_*CH_3_), 1.74 (6H, d, J = 6 Hz, NCH(C*H*_3_)_2_), 1.53 (3H, t, J = 6 Hz, OCH_2_C*H_3_*), 1.47 (3H, t, J = 6 Hz, -CH_2_C*H_3_*). ES + HRMS: Calculated M + H = 235.1559. C_12_H_19_N_4_O. Obtained: 235.1557.

#### 6-Ethoxy-8-ethyl-9-(n-butyl)-9H-purine (A3)

(This compound has already been prepared by us and is described elsewhere^1^). Yellow viscous oil; yield 31%. δ_H_ (200 MHz, CD_3_OD): 8.42 (1H, s, N*CH*N), 4.59 (2H, q, J = 7.1 Hz, OC*H_2_*CH_3_), 4.23 (t, J = 7.4 Hz, 2H), 2.93 (2H, q, J = 7.5 Hz, C*H_2_*CH_3_), 1.79 (2H, m, NCH_2_C*H*_2_), 1.43 (3H, t, J = 7.1 Hz, OCH_2_C*H_3_*), 1.39 (3H, t, J = 7.5 Hz, -CCH_2_C*H_3_*), 1.38 (2H, m, NCH_2_CH_2_C*H*_2_), 0.93 (3H, t, J = 7.4 Hz, NCH_2_CH_2_CH_2_C*H*_3_). δ_C_ (75 MHz, CD_3_OD: 160.3, 156.3, 154.7, 151.4, 121.1, 62.9, 42.9, 32.5, 21.3, 20.5, 14.8, 13.9, 11.4. MS (70 eV) m/z (%): 249.2 (M^+1^); 271.1; 221.1.

#### 6-Ethoxy-9-isopropyl-9H-purine (A4)

Light yellow solid; yield 44%. δ_H_ (499.79 MHz, CDCl_3_): 8.49 (1H, s, N*CH*N), 7.96 (1H, s, NH*CH*N), 4.88 (1H, m, *CH*(CH_3_)_2_), 4.64 (2H, q, J = 5 Hz, OC*H_2_*CH_3_), 1.60 (6H, d, J = 5 Hz, NCH(C*H*_3_)_2_), 1.49 (3H, t, J = 5 Hz, OCH_2_C*H_3_*). δ_C_ (125.68 MHz, CDCl_3_): 160.81, 151.70, 139.53, 121.83, 62.98, 47.33, 22.60, 14.51. ES + HRMS: Calculated M + H = 207.1246. C_10_H_15_N_4_O. Obtained: 207.1245.

#### 6-(Benzyloxy)-9-isopropyl-9H-purine (A5)ASIMJ-4

White solid; yield 38%; mp 167–169°C. δ_H_ (499.79 MHz, CDCl_3_): 8.56 (1H, s, N*CH*N), 8.00 (1H, s, NH*CH*N), 7.56-7.30 (5H, m, *Ph*), 5.69 (2H, s, OC*H_2_*Ph), 4.91 (1H, m, *CH*(CH_3_)_2_), 1.64 (6H, d, J = 10 Hz, NCH(C*H*_3_)_2_). δ_C_ (125.68 MHz, CDCl_3_): 160.53, 151.63, 139.77, 136.27, 130.03, 128.41, 128.29, 128.05, 68.29, 47.43, 22.63. ES + HRMS: Calculated M + H = 269.1402. C_15_H_17_N_4_O. Obtained: 269.1402.

#### Concomitantly preparation of 6-benzyloxy-9-isopropyl-8-phenyl-9H-purine (10) and 6-benzyloxy-9-isopropyl-8-methyl-9H-purine (9) (Fig. 2)

NaH (50%) in mineral oil (3.7 mmol, 10 equiv.) was added to a mixture cooled at 0°C constituted of benzyl alcohol (7.4 mmol, 20 equiv.) and *N,N*-dimethylacetamide (1.8 mL, 18.5 mmol, 50 equiv.). The solution was stirred at room temperature for 30 min and then at 90°C for the same time. 5-amino-6-chloro-4-isopropylaminopyrimidine **8** (0.37 mmol, 1 equiv.) dissolved in *N,N*-dimethylacetamide (1.8 mL, 18.5 mmol, 50 equiv.) was added dropwise to the mixture which was then heated for 24 h at 90°C. The reaction mixture was brought to pH 7 with an aq. saturated NH_4_Cl solution, and then extracted with CH_2_Cl_2_ (3 × 15 mL). The combined organic extracts were dried over Na_2_SO_4,_ filtered and the organic solvent evaporated. Crudes were purified by flash chromatography on silica gel using ethyl acetate/petroleum ether as mobile phases. Compounds **9** and **10** were isolated with 20% yield each.

#### 6-Benzyloxy-9-isopropyl-8-methyl-9H-purine (9; Fig. 2)

White solid; yield 20%. δ_H_ (499.79 MHz, CDCl_3_): 8.47 (1H, s, N*CH*N), 7.54–7.30 (5H, m, Ph), 5.65 (2H, s, OC*H_2_*Ph), 4.75 (1H, m, *CH*(CH_3_)_2_), 2.65 (3H, s, C*H_3_*), 1.69 (6H, d, J = 10 Hz, NCH(C*H*_3_)_2_). δ_C_ (125.68 MHz, CDCl_3_): 159.41, 153.57, 150.69, 150.41, 136.42, 128.43 128.35, 127.98, 120.73, 68.06, 48.35, 21.23, 15.22. ES + HRMS: Calculated M + H = 283.1559. C_16_H_19_N_4_O. Obtained: 283.1555.

#### 6-Benzyloxy-9-isopropyl-8-phenyl-9H-purine (10; Fig. 2)

White solid, mp: 111–112°C; yield 20%. δ_H_ (300.20 MHz, CDCl_3_): 8.57 (1H, s, N*CH*N), 7.70–7.38 (10H, m, *Ph,* CH_2_*Ph*), 5.72 (2H, s, OC*H_2_*Ph), 4.80 (1H, m, *CH*(CH_3_)_2_), 1.76 (6H, d, J = 6 Hz, NCH(C*H*_3_)_2_). δ_C_ (125.68 MHz, CDCl_3_): 160.54, 153.14, 151.01, 136.66, 130.40, 129.80, 128.95, 128.76, 128.61, 128.27, 128.05, 122.14, 68.42, 50.04, 21.51. ES + HRMS: Calculated M + H = 345.1715. C_21_H_21_N_4_O. Obtained: 345.1716.

#### Preparation of N^4^-benzyl-N^5^-benzylidene-6-(benzyloxy)pyrimidine-4,5-diamine (13) (Fig. 4)

NaH (50%) in mineral oil (3.7 mmol, 10 equiv.) was added to a mixture cooled at 0°C constituted of benzyl alcohol (3.7 mmol, 10 equiv.) and *N,N*-dimethylbenzamide (18.5 mmol, 50 equiv.) in 1,4-dioxane (2 mL). The solution was stirred at room temperature for 30 min and then at 90°C for the same time. 5-amino-4-benzylamino-6-chloropyrimidine **12** dissolved in 1,4-dioxane (2 mL) was added dropwise to the mixture which was then heated for 24 h at 90°C. The reaction mixture was brought to pH 7 with an aq. saturated NH_4_Cl solution, and then extracted with CH_2_Cl_2_ (3 × 15 mL). The combined organic extracts were dried over Na_2_SO_4,_ filtered and the organic solvent evaporated. Crudes were purified by flash chromatography on silica gel using ethyl acetate/petroleum ether as mobile phases. Yellow oil; yield 10%. δ_H_ (499.79 MHz, CDCl_3_): 9.23 (1H, s, N*CH*Ph), 8.21 (1H, s, N*CH*N), 8.01–7.35 (15H, m,NCH*Ph,* NHCH*_2_Ph,* OCH*_2_Ph*), 5.53 (2H, s, OC*H_2_*Ph), 5.46 (2H, s, NHC*H_2_*Ph), 4.82 (1H, d, J = 5, Hz, N*H*). δ_C_ (125.68 MHz, CDCl_3_): 161.73, 158.71, 156.73, 144.68, 136.75, 134, 131.15, 128.71, 128.52, 128.10, 127.71, 127.34, 127.24, 114.20, 68.30, 44.98. ES + HRMS: Calculated M + H = 395.1872. C_25_H_23_N_4_O. Obtained: 395.1873.

#### Concomitantly preparation of 6-benzyloxy-9-isopropyl-8-phenyl-9H-purine (10) and N^5^-benzylidene-6-(benzyloxy)-N^4^-isopropylpyrimidine-4,5-diamine (15) (Fig. 5 (i))

In this case, in order to eliminate oxygen from the reaction, this was performed under argon atmosphere. Furthermore, *N,N*-dimethylpropionamide was degassed by bubbling an argon stream assisted by an ultrasound device. NaH (50%) in mineral oil (3.7 mmol, 10 equiv.) was added to a mixture cooled at 0°C constituted of benzyl alcohol (3.7 mmol, 10 equiv.) and *N,N*-dimethylpropionamide (2.3 mL, 18.5 mmol, 50 equiv.). The solution was stirred at room temperature for 30 min and then at 90°C for the same time. 5-amino-6-chloro-4-isopropylaminopyrimidine **8** (0.37 mmol, 1 equiv.) dissolved in *N,N*-dimethylpropionamide (2.3 mL, 18.5 mmol, 50 equiv.) was added dropwise to the mixture which was then heated for 24 h at 90°C. The reaction mixture was brought to pH 7 with an aq. saturated NH_4_Cl solution, and then extracted with CH_2_Cl_2_ (3 × 15 mL). The combined organic extracts were dried over Na_2_SO_4,_ filtered and the organic solvent evaporated. Crudes were purified by flash chromatography on silica gel using ethyl acetate/petroleum ether as mobile phases. Compounds **10** and **15** were isolated with 20% yield each.

#### 6-Benzyloxy-9-isopropyl-8-phenyl-9H-purine (10; Fig. 5 (i))

White solid, mp:111–112°C; yield 23%. δ_H_ (300.20 MHz, CDCl_3_): 8.57 (1H, s, N*CH*N), 7.70–7.38 (10H, m, *Ph,* CH_2_*Ph*), 5.72 (2H, s, OC*H_2_*Ph), 4.80 (1H, m, *CH*(CH_3_)_2_), 1.76 (6H, d, J = 6 Hz, NCH(C*H*_3_)_2_). δ_C_ (125.68 MHz, CDCl_3_): 160.54, 153.14, 151.01, 136.66, 130.40, 129.80, 128.95, 128.76, 128.61, 128.27, 128.05, 122.14, 68.42, 50.04, 21.51. ES + HRMS: Calculated M + H = 345.1715. C_21_H_21_N_4_O. Obtained: 345.1718.

#### N^5^-Benzylidene-6-(benzyloxy)-N^4^-isopropylpyrimidine-4,5-diamine (15; Fig. 5 (i))

Yellow oil; yield 10%. δ_H_(499.79 MHz, CDCl_3_): 9.19 (1H, s, N*CH*Ph), 8.18 (1H, s, N*CH*N), 7.79 (2H, s, H-2′NCH*Ph*), 7.47–7.30 (8H, m, *Ph,* CH*Ph*), 6.02 (1H, d, J = 10 Hz, N*H*), 5.51 (2H, s, OC*H_2_*Ph), 4.35 (1H, m, *CH*(CH_3_)_2_), 1.32 (6H, d, J = 5 Hz, NCH(C*H*_3_)_2_). δ_C_(125.68 MHz, CDCl_3_): 161.40, 158.53, 154.40, 137.34, 136.95, 134.49, 131.08, 128.75, 128.21, 127.68, 110.91, 68.02, 42.73, 23.23. ES + HRMS: Calculated M + H = 347.1872. C_21_H_23_N_4_O. Obtained: 347.1873.

#### Reaction of 5-amino-6-chloro-4-isopropylaminopyrimidine (8) with benzyl alcohol and without amide (Fig. 5 (ii))

NaH (50%) in mineral oil (3.7 mmol, 10 equiv.) was added to a mixture cooled at 0°C constituted of benzyl alcohol (3.7 mmol, 10 equiv.) and 1,4-dioxane (2 mL). The solution was stirred at room temperature for 30 min and then at 90°C for the same time. 5-amino-6-chloro-4-isopropylaminopyrimidine **8** (0.37 mmol, 1 equiv.) dissolved in 1,4-dioxane (2 mL) was added dropwise to the mixture which was then heated for 24 h at 90°C. The reaction mixture was brought to pH 7 with an aq. saturated NH_4_Cl solution, and then extracted with CH_2_Cl_2_ (3 × 15 mL). The combined organic extracts were dried over Na_2_SO_4,_ filtered and the organic solvent evaporated. Crudes were purified by flash chromatography on silica gel using ethyl acetate/petroleum ether. In this case, the starting materials were recovered.

#### Preparation of 6-benzyloxy-9-isopropyl-8-phenyl-9H-purine (10) and N^5^-benzylidene-6-(benzyloxy)-N^4^-isopropylpyrimidine-4,5-diamine (15) (Fig. 5 (iii)

NaH (50%) in mineral oil (3.7 mmol, 10 equiv.) was added to a mixture cooled at 0°C constituted of benzaldehyde (3.7 mmol, 10 equiv.) and 1,4-dioxane (2 mL). The solution was stirred at room temperature for 30 min and then at 90°C for the same time. 5-amino-6-chloro-4-isopropylaminopyrimidine **8** (0.37 mmol, 1 equiv.) dissolved in 1,4-dioxane (2 mL) was added dropwise to the mixture which was then heated for 24 h at 90°C. The reaction mixture was brought to pH 7 with an aq. saturated NH_4_Cl solution, and then extracted with CH_2_Cl_2_ (3 × 15 mL). The combined organic extracts were dried over Na_2_SO_4,_ filtered and the organic solvent evaporated. Crudes were purified by flash chromatography on silica gel using ethyl acetate/petroleum ether as mobile phases. Compounds **10** and **15** were isolated with a 23% yield and 10% yield respectively.

#### 6-Benzyloxy-9-isopropyl-8-phenyl-9H-purine (10; Fig. 5 (iii))

White solid; yield 23%. δ_H_ (300 MHz, CDCl_3_): 8.57 (1H, s, N*CH*N), 7.70–7.38 (10H, m, *Ph,* CH_2_*Ph*), 5.72 (2H, s, OC*H_2_*Ph), 4.80 (1H, m, *CH*(CH_3_)_2_), 1.76 (6H, d, J = 6 Hz, NCH(C*H*_3_)_2_). δ_C_ (125.68 MHz, CDCl_3_): 160.54, 153.14, 151.01, 136.66, 130.40, 129.80, 128.95, 128.76, 128.61, 128.27, 128.05, 122.14, 68.42, 50.04, 21.51. ES + HRMS: Calculated M + H = 345.1715. C_21_H_21_N_4_O. Obtained: 345.1718.

#### N^5^-Benzylidene-6-(benzyloxy)-N^4^-isopropylpyrimidine-4,5-diamine (15; Fig. 5 (iii))

Yellow oil; yield 10%. δ_H_ (499.79 MHz, CDCl_3_): 9.19 (1H, s, N*CH*Ph), 8.18 (1H, s, N*CH*N), 7.79 (2H, s, H-2′NCH*Ph*), 7.47–7.30 (8H, m,*Ph,* CH*Ph*), 6.02 (1H, d, J = 10 Hz, N*H*), 5.51 (2H, s, OC*H_2_*Ph), 4.35 (1H, m, *CH*(CH_3_)_2_), 1.32 (6H, d, J = 5 Hz, NCH(C*H*_3_)_2_). δ_C_ (125.68 MHz, CDCl_3_): 161.40, 158.53, 154.40, 137.34, 136.95, 134.49, 131.08, 128.75, 128.21, 127.68, 110.91, 68.02, 42.73, 23.23. ES + HRMS: Calculated M + H = 347.1872. C_21_H_23_N_4_O. Obtained: 347.1873.

### Biological assays

#### Cell culture

*T. brucei* subsp. *brucei*, strain Lister 427 VSG 221 were grown in axenic culture at 37°C and 5% CO_2_ in HMI-9 media supplemented with 10% heat-inactivated foetal bovine serum (Gibco).

#### Trypanotoxicity assays

The sensitivity of trypanosomes to the whole library of purine derivatives was assessed using resazurin sodium protocol as described previously^42^. Exponentially growing parasites were harvested and prepared at initial density of 2 × 10^5^ trypanosomes per mL. Each well of a 96-well tissue culture plate containing 50 μL from a serial doubling dilutions of drug was inoculated with 50 μL of trypanosome culture, with the exception of two rows which received only media. Eleven different final concentrations of compounds, ranging from 0.5 to 500 μM per assay were tested in a six-replicate format. Each assay was repeated three times. The same volume of solvent (DMSO) at each concentration set point was tested in parallel. Parasites were incubated for 20 h at 37°C and 5% CO_2_. Then, 20 μL of 0.5 mM resazurin dye (Sigma, R7017) was added to each well and plates were incubated for a further 4 h. The reaction was stopped by adding 50 μL of 3%sodium dodecyl sulfate (SDS) in PBS and then read on a Tecan Infinite F200 reader (Tecan Austria GmbH, Austria) using an excitation wavelength of 535 nm and an emission wavelength of 590 nm. Half-inhibitory concentration (IC_50_) value was calculated using GraphPad Prism5 Software and defined as the concentration of drug required to diminish fluorescence output by 50%. Data are expresses as the IC_50_ mean value ± standard error of the mean (S.E.M.)

#### Confocal Microscopy

Morphological phenotype was analyzed by fluorescence and optical microscopy (DIC, Differential Interference Contrast). After 24 hours of purines treatment using their IC_50_ concentration values, parasites were fixed in 4% paraformaldehyde (PFA). Then, trypanosomes were washed three times and spread on poly-L-lysine-coated slides and mounted in DAPI-containing Vectashield medium (Vector Laboratories, Burlingame, CA, USA). Image acquisition was performed with Confocal Scanning Microscope Zeiss LSM 710 and Zen 2012 software.

## Supplementary Material

Supplementary Information Supplementary Material

## Figures and Tables

**Table 1:** List of starting materials employed and the types of poly-substituted purines synthesized. Half-inhibitory concentrations (IC_50_) against the protozoan parasite *Trypanosoma brucei* presented as mean ± SEM. n = 6

entry	R^1^	R^2^	R^3^	“A”% (R^1^, R^2^, R^3^)	“B”% (R^2^,R^3^)	IC_50_ (μM)^a^ *T.brucei*
A1	Me	Ph	^i^Pr	16%	traces	69.2 ± 1.8
A2	Et	Me	^i^Pr	29%	traces	137.1 ± 6.9
A3^1^	Et	Me	Bu	31%	traces	51.8 ± 3.3
A4	H	Me	^i^Pr	44%	-	14.7 ± 0.6
A5	H	Ph	^i^Pr	38%	-	1 ± 0.1
B1	Et	Ph	^i^Pr	-	40%	52.1 ± 1
B2	Et	Ph	^t^Bu	-	45%	18.3 ± 0.5
B3	Et	MeOPh	^i^Pr	-	28%	42.7 ± 1
B4	Et	MeOPh	^t^Bu	-	22%	80.5 ± 3.5
B5	^i^Pr	H	^i^Pr	-	10%	37.5 ± 1.5
B6	^i^Pr	Me	^i^Pr	-	10%	32 ± 1.7
B7	Ph	Me	^i^Pr	-	20%	22.7 ± 1.4
B8	Ph	Et	^i^Pr	-	27%	4.8 ± 0.2
B9	Ph	^i^Pr	^i^Pr	-	22%	12.6 ± 0.6

*^a^*Cell viability was assessed by resazurin dye. Half-inhibitory concentration (IC_50_) values were calculated using GraphPad Prism5 Software and defined as the concentration of purine required to diminish fluorescence output by 50%. n = 6.

## Notes

**Supplementary Material**: ^1^H NMR, ^13^C NMR and HRMS spectra of all the compounds presented in this study. HSQC spectrum of compound **15**.
