# Supplementary material for: Amide-controlled, one-pot synthesis of tri-substituted purines generates structural diversity and analogues with trypanocidal activity
Source: Sci Rep. 2015 Mar 16;5:9139. doi: 10.1038/srep09139 (PMC5390905; doi:10.1038/srep09139)

Supplementary Information

Amide-controlled, one-pot synthesis of tri-substituted purines generates structural diversity and analogues with trypanocidal activity

Maria J. Pineda de las Infantas y Villatoro[a],*, Juan D. Unciti-Broceta[b], Rafael Contreras Montoya[b], Jose A. Garcia-Salcedo[b,c], Miguel A. Gallo Mezo[a], Asier Unciti-Broceta[d],* and Juan J. Diaz-Mochon[a,b,*]

[a] *Departamento de Química Farmacéutica y Orgánica. University of Granada, Campus de Cartuja s/n, 18071 Granada, Spain*

[b] *Pfizer - Universidad de Granada - Junta de Andalucía Centre for Genomics and Oncological Research (GENYO), Parque Tecnológico de Ciencias de la Salud (PTS), Avenida de la Ilustración 114, 18016 Granada, Spain*

[c] *Unidad de Enfermedades Infecciosas, Hospital Universitario San Cecilio, Instituto de Investigación Biosanitaria de Granada, Dr. Azpitarte, 4, 18012 Granada, Spain*

[d] *Edinburgh Cancer Research UK Centre, MRC Institute of Genetics and Molecular Medicine, University of Edinburgh, Crewe Road South, Edinburgh EH4 2XR, UK.*

* To whom the correspondence should be addressed. E-mail: mjpineda@ugr.es, Asier.Unciti-Broceta@igmm.ed.ac.uk, juanjose.diaz@genyo.es

8. 5-amino-6-chloro-4-isopropylaminopyrimidine (H-NMR)

17 Jul 2012

Acquisition Time (sec)	2.5559	***Date***	Jan 31 2012
File Name	C:\Users\usuario\Documents\Espectros Asier\PRE-AM1	***Frequency (MHz)***	400.58
Nucleus	1H	***Number of Transients***	8
Original Points Count	16384	***Points Count***	16384
Pulse Sequence	s2pul	***Solvent***	CHLOROFORM-D
Sweep Width (Hz)	6410.26	***Temperature (degree C)***	25.000

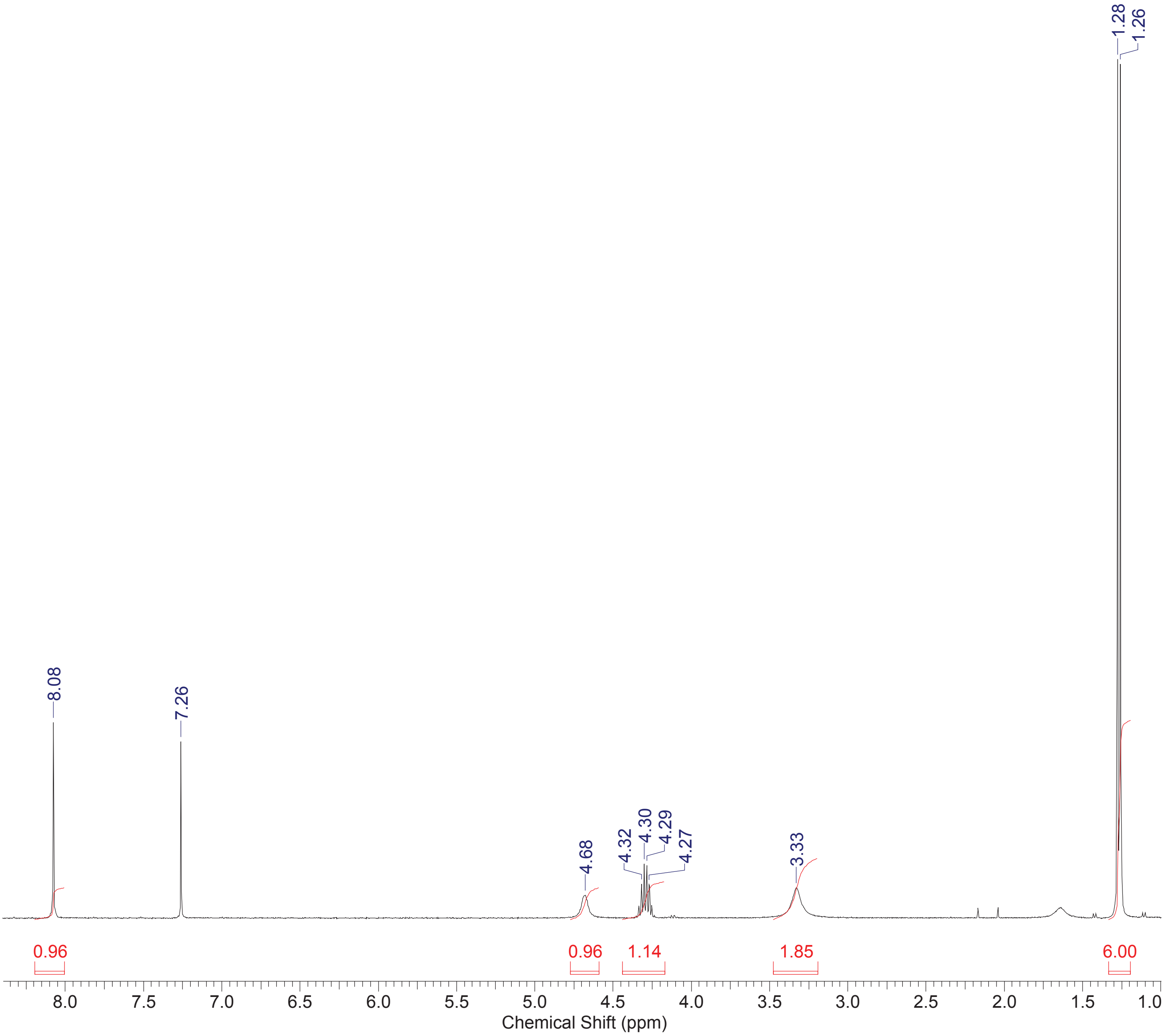

No.	(ppm)	(Hz)	Height
1	1.26	504.8	0.9945
2	1.28	511.1	1.0000
3	3.33	1333.5	0.0371
4	4.27	1710.0	0.0404
5	4.29	1716.6	0.0631
6	4.30	1723.3	0.0649
7	4.32	1729.9	0.0412
8	4.68	1873.9	0.0284
9	7.26	2908.4	0.2072
10	8.08	3235.1	0.2294

No.	(ppm)	Value	Absolute Value
1	[1.19 .. 1.34]	6.000	2.56874e+7
2	[3.19 .. 3.48]	1.853	7.93366e+6
3	[4.17 .. 4.44]	1.143	4.89318e+6
4	[4.59 .. 4.77]	0.960	4.11209e+6
5	[8.00 .. 8.20]	0.960	4.11063e+6

12. 5-Amino-4-benzylamino-6-chloropyrimidine (C-NMR)

8 Jul 2013

Acquisition Time (sec)	1.0486	***Date***	Mar 6 2012		
File Name	C:\Users\usuario\Documents\Espectros Asier\12-511_Pre-2\carbono				
Frequency (MHz)	125.68	***Nucleus***	13C	***Original Points Count***	32768
Points Count	32768	***Pulse Sequence***	s2pul	***Solvent***	CHLOROFORM-D
Sweep Width (Hz)	31250.00	***Temperature (degree C)***	25.000		

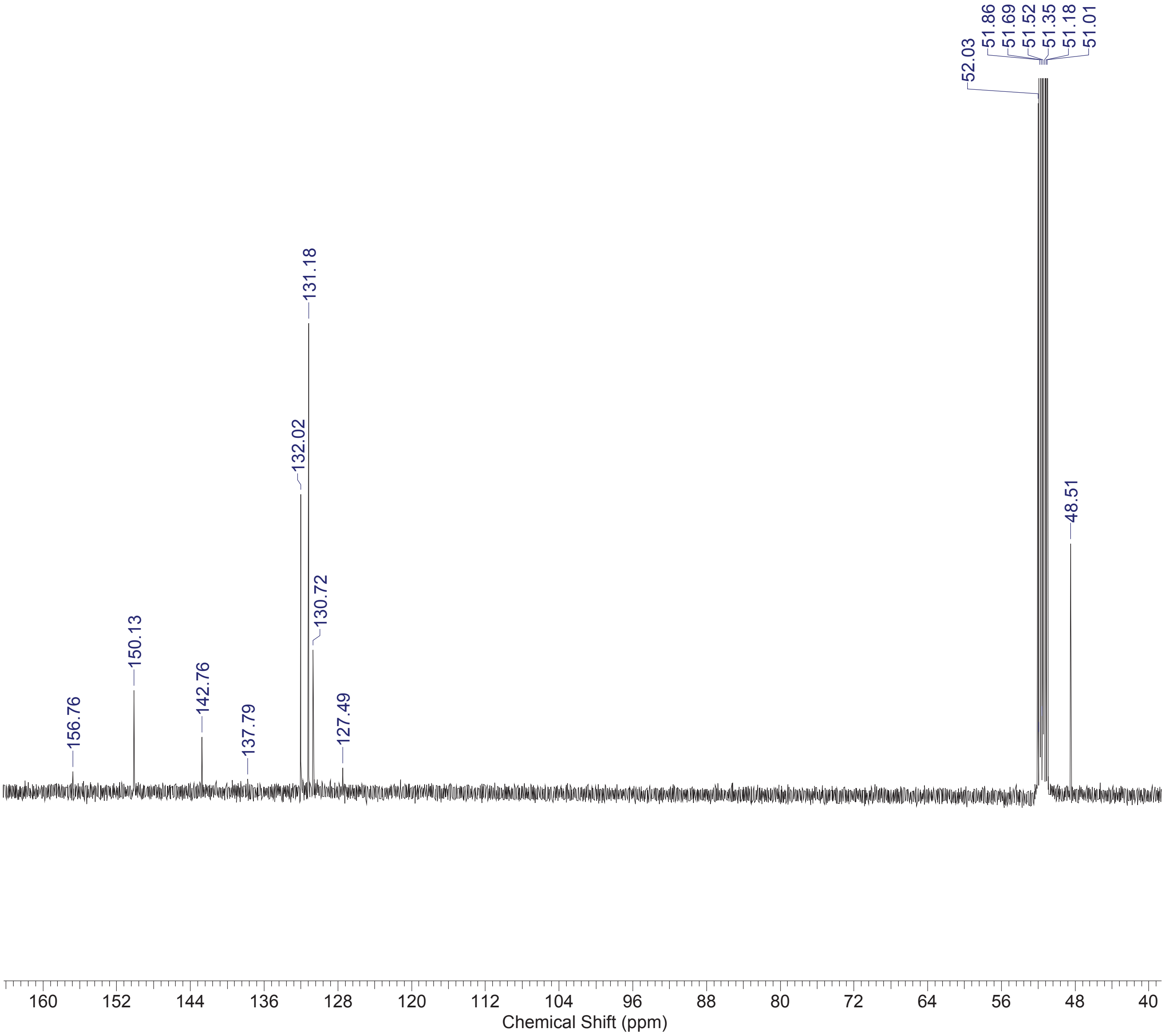

No.	(ppm)	(Hz)	Height
1	48.51	6096.4	0.0362
2	51.01	6411.1	0.1618
3	51.18	6432.1	0.3818
4	51.35	6454.0	0.8678
5	51.52	6475.0	1.0000
6	51.63	6489.3	0.0104
7	51.69	6496.9	0.7416
8	51.81	6511.3	0.0145
9	51.86	6517.9	0.4658
10	51.97	6532.2	0.0080

No.	(ppm)	(Hz)	Height
11	52.03	6539.9	0.1005
12	127.49	16023.5	0.0035
13	130.72	16429.8	0.0207
14	131.18	16487.0	0.0684
15	132.02	16592.8	0.0434
16	137.79	17318.6	0.0019
17	142.76	17943.3	0.0080
18	150.13	18869.3	0.0148
19	156.76	19701.9	0.0029

12. 5-Amino-4-benzylamino-6-chloropyrimidine (H-NMR)

8 Jul 2013

Acquisition Time (sec)	1.7432	***Date***	Mar 6 2012		
File Name	C:\Users\usuario\Documents\Espectros Asier\12-511_Pre-2\proton				
Frequency (MHz)	499.79	***Nucleus***	1H	***Number of Transients***	80
Original Points Count	13048	***Points Count***	16384	***Pulse Sequence***	s2pul
Solvent	METHANOL-D4			***Sweep Width (Hz)***	7485.03
Temperature (degree C)	25.000				

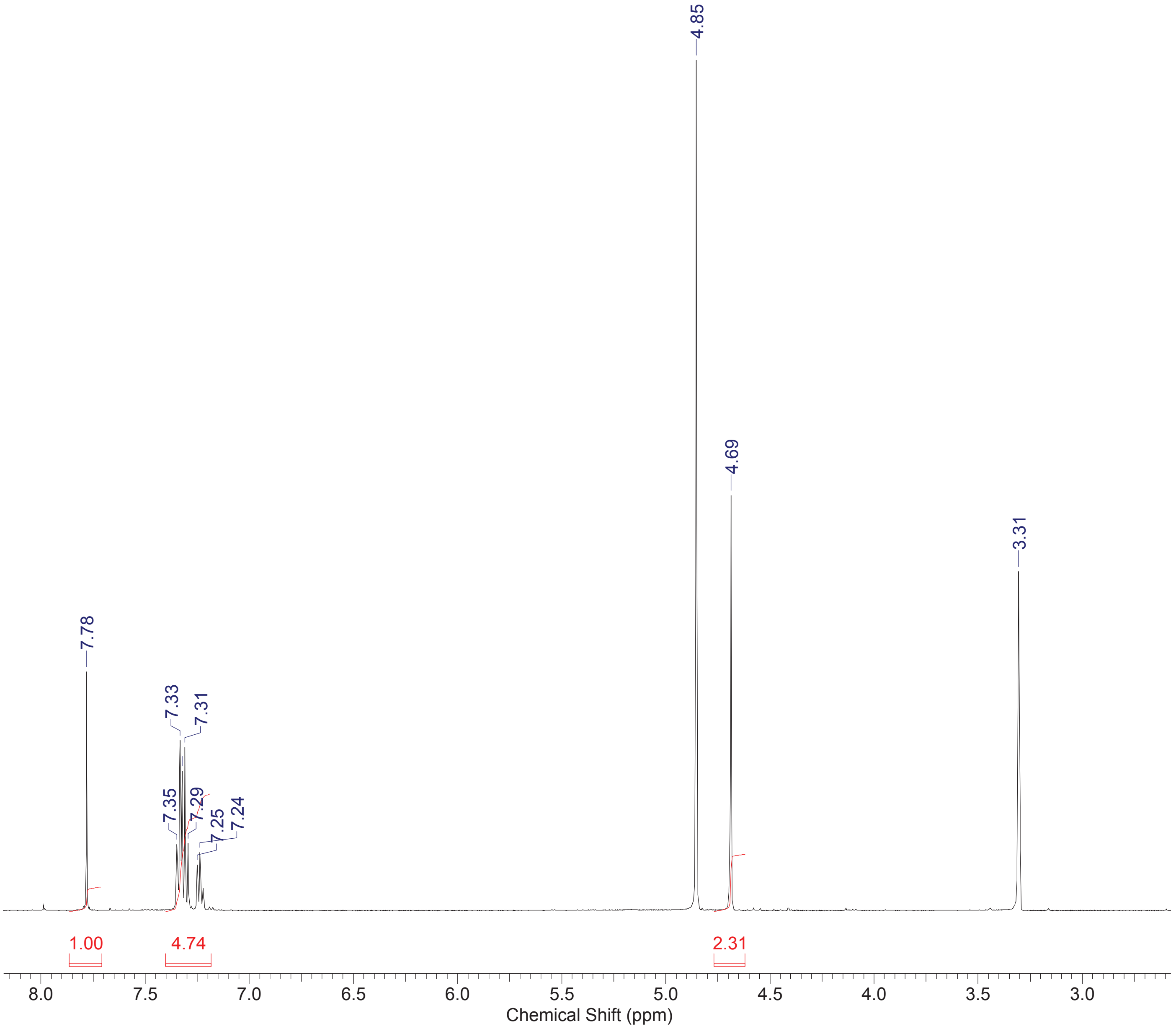

No.	(ppm)	(Hz)	Height
1	3.31	1652.0	0.3992
2	4.69	2342.8	0.4889
3	4.85	2425.5	1.0000
4	7.24	3616.6	0.0695
5	7.25	3623.4	0.0555
6	7.29	3644.9	0.0804
7	7.31	3652.7	0.1928
8	7.32	3660.0	0.1651
9	7.33	3665.0	0.2014
10	7.35	3671.9	0.0790
11	7.78	3889.3	0.2820

No.	(ppm)	Value	Absolute Value
1	[4.62 .. 4.77]	2.308	5.89304e+7
2	[7.18 .. 7.40]	4.741	1.21016e+8
3	[7.71 .. 7.87]	1.000	2.55278e+7

12. 5-Amino-4-benzylamino-6-chloropyrimidine (MS)

Elemental Composition Report

Single Mass Analysis

Tolerance = 5.0 mDa / DBE: min = -1.5, max = 50.0
Element prediction: Off
Number of isotope peaks used for i-FIT = 3

Monoisotopic Mass, Even Electron Ions
383 formula(e) evaluated with 7 results within limits (up to 50 best isotopic matches for each mass)
Elements Used:
C: 0-11 H: 0-1000 N: 0-9 O: 0-20 Cl: 0-1

MJ-2 89 (1.975)

1: TOF MS ES+

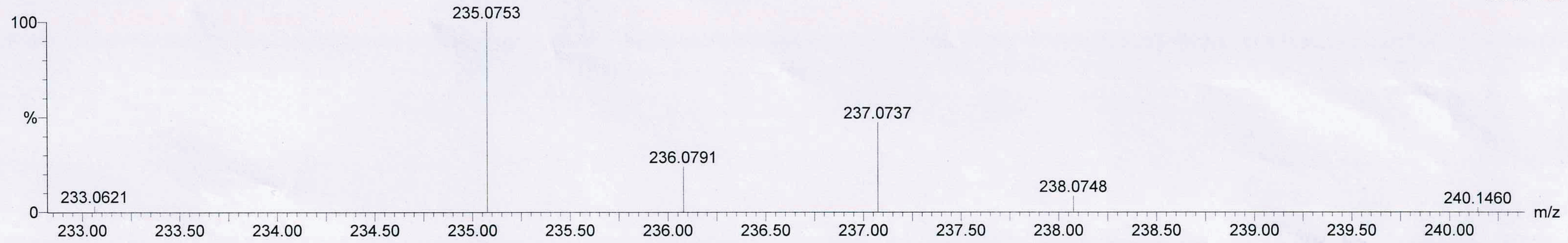

		mDa	PPM	DBE	i-FIT	Formula
Minimum:				-1.5		
Maximum:		5.0	10.0	50.0		

Mass	Calc. Mass	mDa	PPM	DBE	i-FIT	Formula
235.0753	235.0750	0.3	1.3	7.5	24.3	C11 H12 N4 Cl
	235.0737	1.6	6.8	2.5	31.4	C10 H16 O4 Cl
	235.0710	4.3	18.3	3.5	43.6	C6 H12 N6 O2 Cl
	235.0719	3.4	14.5	7.5	173.8	C11 H11 N2 O4
	235.0791	-3.8	-16.2	3.5	198.1	C5 H11 N6 O5
	235.0778	-2.5	-10.6	-1.5	205.2	C4 H15 N2 O9
	235.0751	0.2	0.9	-0.5	222.4	H11 N8 O7

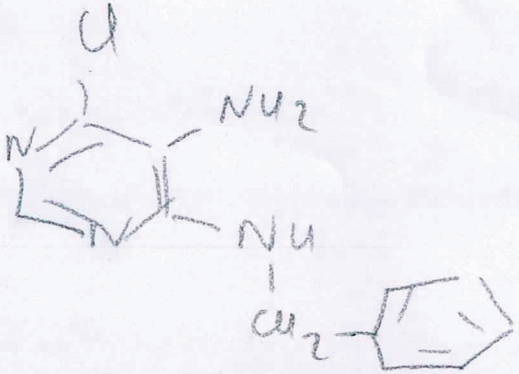

13. N4-benzyl-N5-benzylidene-6-(benzyloxy)pyrimidine-4,5-diamine (C-NMR)

8 Jul 2013

Acquisition Time (sec)	1.0486	***Date***	Nov 23 2012		
File Name	C:\Users\usuario\Documents\Espectros Asier\12-23752_ASIMJ-30-1\carbono				
Frequency (MHz)	125.68	***Nucleus***	13C	***Number of Transients***	4800
Original Points Count	32768	***Points Count***	32768	***Pulse Sequence***	s2pul
Solvent	CHLOROFORM-D			***Sweep Width (Hz)***	31250.00
Temperature (degree C)	25.000				

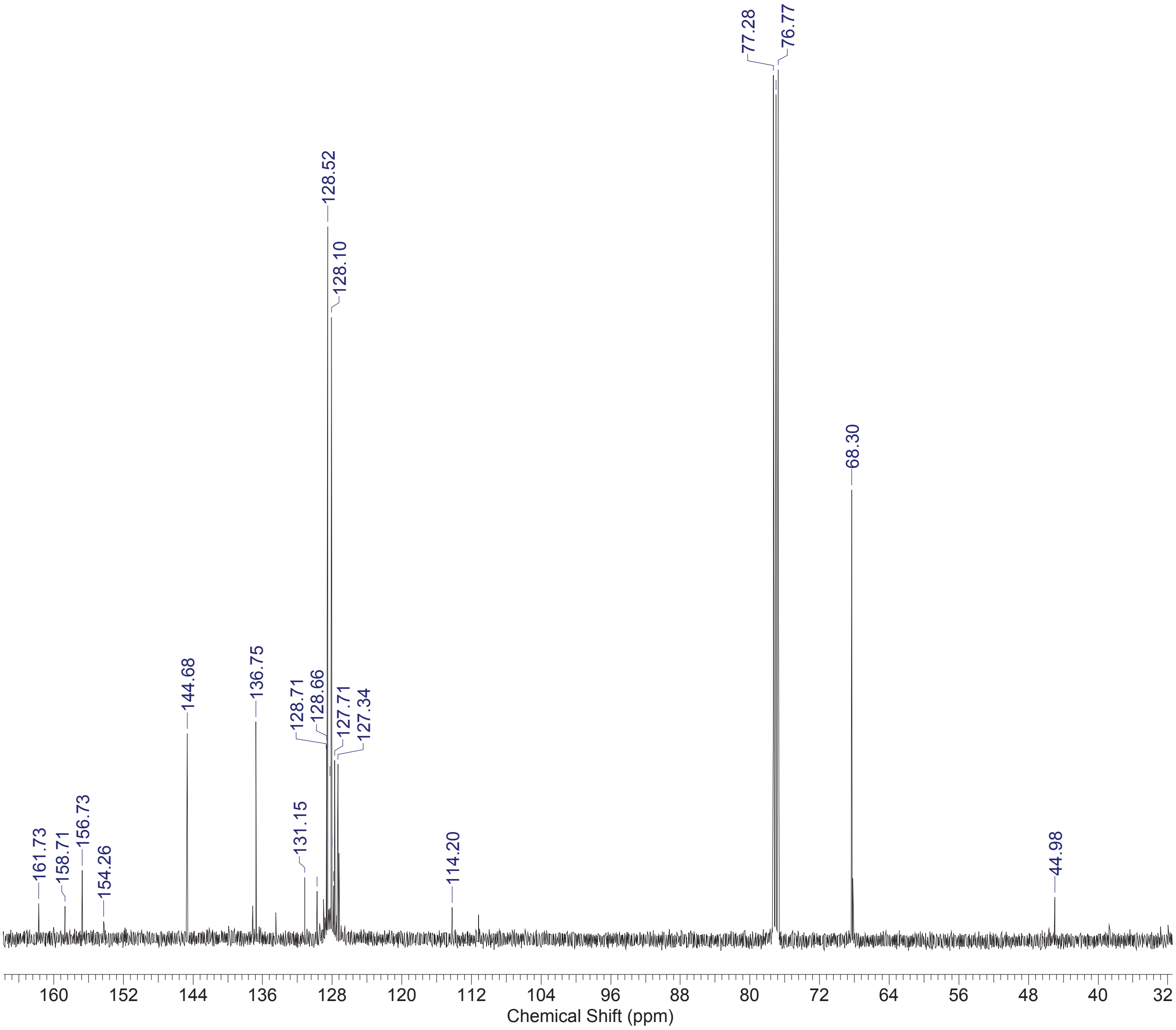

No.	(ppm)	(Hz)	Height	No.	(ppm)	(Hz)	Height
1	44.98	5652.9	0.0478	13	128.24	16117.9	0.1818
2	68.30	8583.6	0.5167	14	128.52	16153.2	0.8196
3	76.77	9648.9	1.0000	15	128.66	16170.4	0.2225
4	77.03	9681.4	0.9718	16	128.71	16177.0	0.2132
5	77.28	9712.8	0.9939	17	129.76	16308.6	0.0542
6	114.20	14353.6	0.0357	18	131.15	16483.2	0.0704
7	127.24	15992.0	0.0981	19	136.75	17187.0	0.2494
8	127.34	16004.4	0.2005	20	144.68	18184.6	0.2363
9	127.71	16051.1	0.2053	21	154.26	19388.2	0.0201
10	127.85	16069.3	0.0603	22	156.73	19698.1	0.0787
11	128.01	16088.3	0.1016	23	158.71	19948.0	0.0371
12	128.10	16099.8	0.7153	24	161.73	20326.6	0.0404

13. N4-benzyl-N5-benzylidene-6-(benzyloxy)pyrimidine-4,5-diamine (H-NMR)

8 Jul 2013

**Acquisition Time (sec)**	2.0447	**Date**	Nov 23 2012		
**File Name**	C:\Users\usuario\Documents\Espectros Asier\12-23752_ASIMJ-30-1\proton				
**Frequency (MHz)**	499.79	**Nucleus**	1H	**Number of Transients**	8
**Original Points Count**	16384	**Points Count**	16384	**Pulse Sequence**	s2pul
**Solvent**	CHLOROFORM-D			**Sweep Width (Hz)**	8012.82
**Temperature (degree C)**	25.000				

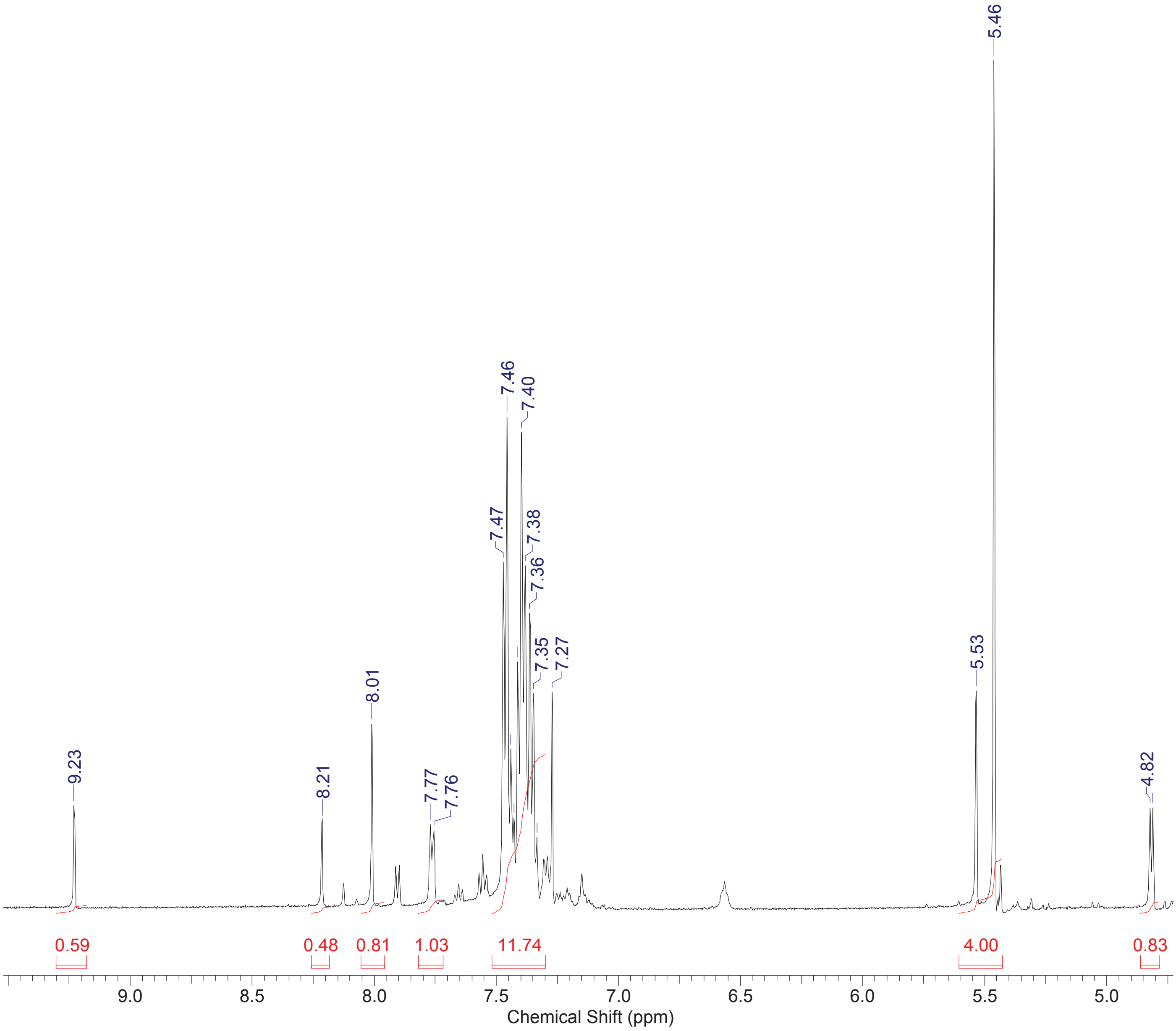

No.	(ppm)	(Hz)	Height
1	4.81	2404.2	0.1236
2	4.82	2410.0	0.1237
3	5.46	2729.4	1.0000
4	5.53	2766.1	0.2609
5	7.27	3634.7	0.2593
6	7.33	3665.6	0.0892
7	7.35	3672.9	0.2579
8	7.36	3680.2	0.3517
9	7.38	3689.5	0.4076
10	7.40	3696.9	0.5635

No.	(ppm)	(Hz)	Height
11	7.41	3704.2	0.2949
12	7.43	3712.0	0.1116
13	7.44	3718.4	0.1921
14	7.46	3726.7	0.5819
15	7.47	3734.5	0.4113
16	7.76	3876.4	0.0969
17	7.77	3883.7	0.1042
18	8.01	4003.5	0.2218
19	8.21	4104.8	0.1094
20	9.23	4612.9	0.1266

No.	(ppm)	Value	Absolute Value
1	[4.79 .. 4.86]	0.828	2.78104e+6
2	[5.43 .. 5.61]	4.000	1.34299e+7
3	[7.30 .. 7.52]	11.745	3.94331e+7
4	[7.72 .. 7.82]	1.026	3.44435e+6
5	[7.96 .. 8.05]	0.815	2.73543e+6
6	[8.18 .. 8.26]	0.477	1.60142e+6
7	[9.18 .. 9.30]	0.590	1.98125e+6

15. N5-benzylidene-6-(benzyloxy)-N4-isopropylpyrimidine-4_,5-diamine HSQC

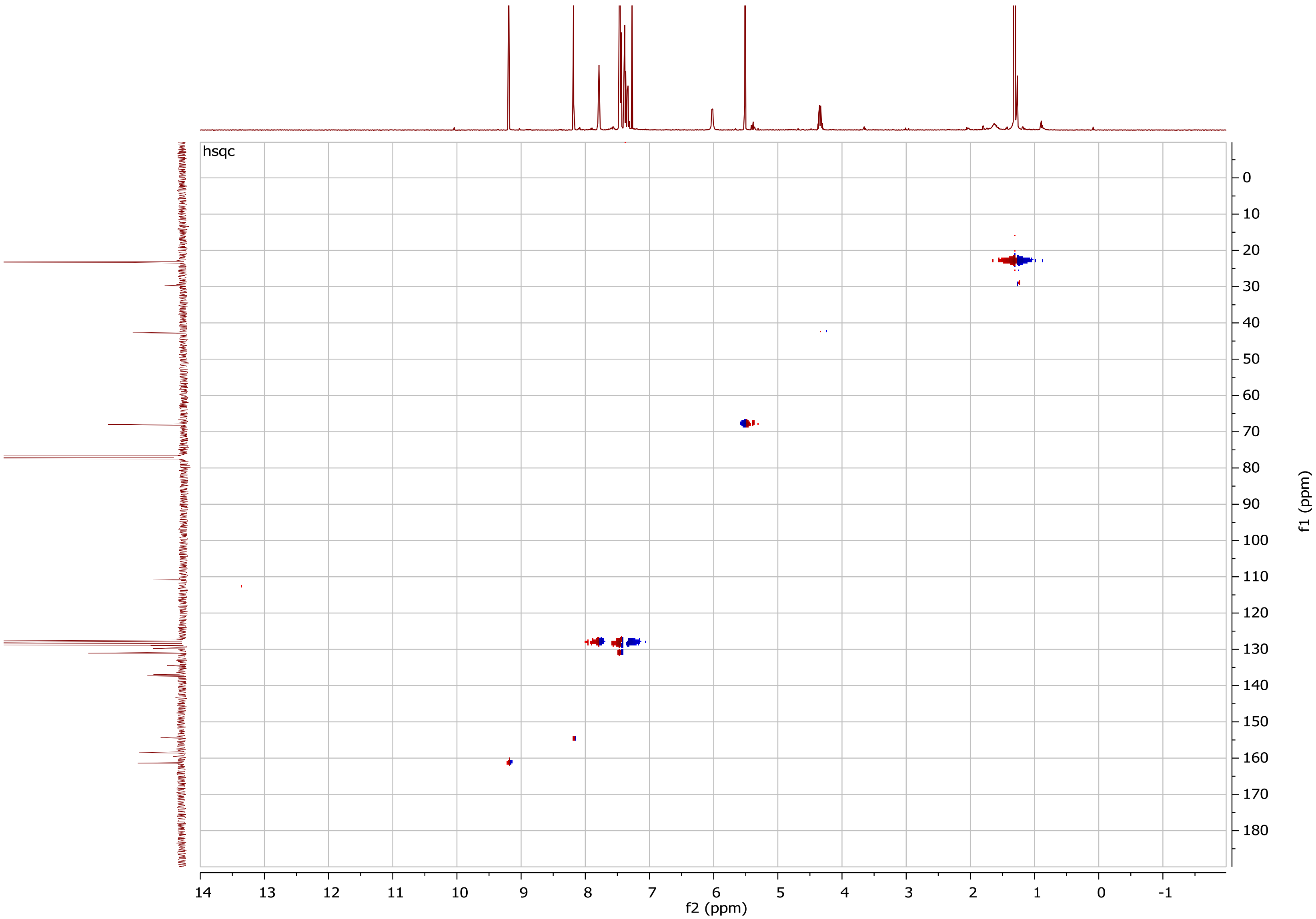

15. N5-benzylidene-6-(benzyloxy)-N4-isopropylpyrimidine-4_,5-diamine HSQC Zoom in

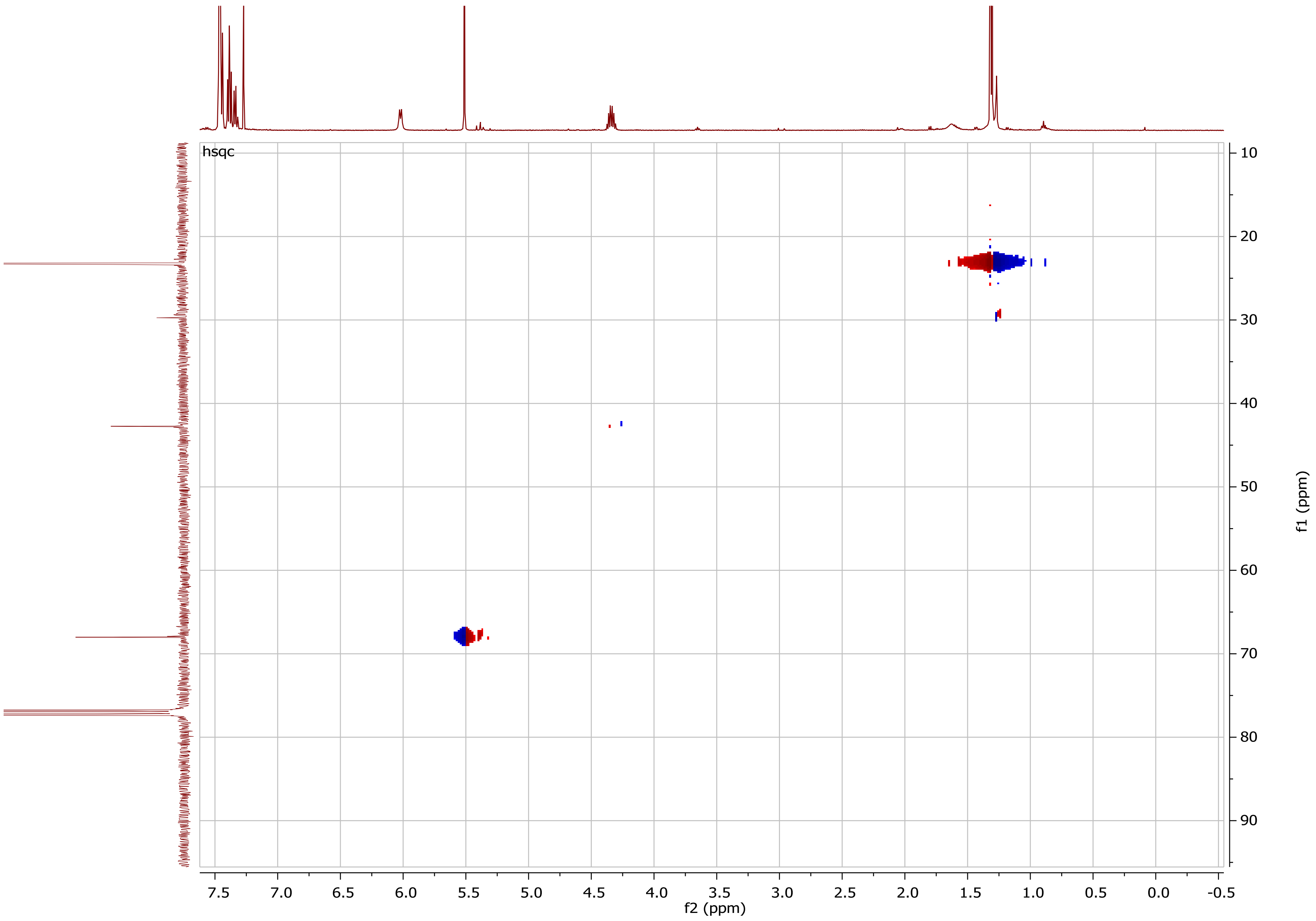

15. N5-benzylidene-6-(benzyloxy)-N4-isopropylpyrimidine-4_,5-diamine HSQC Zoom in

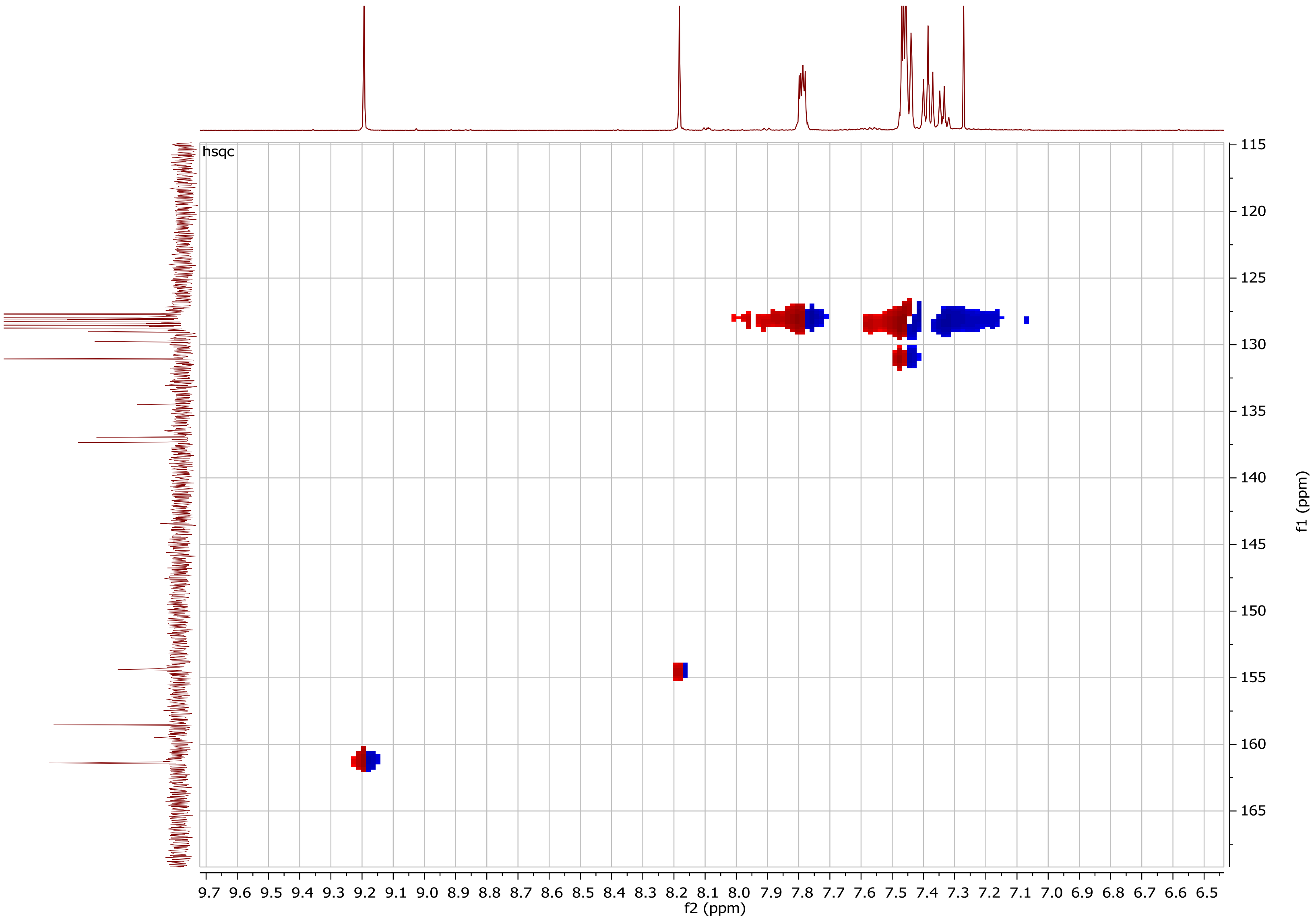

13. N4-benzyl-N5-benzylidene-6-(benzyloxy)pyrimidine-4,5-diamine (MS)

Elemental Composition Report

Single Mass Analysis

Tolerance = 5.0 PPM / DBE: min = -1.5, max = 50.0
Element prediction: Off
Number of isotope peaks used for i-FIT = 3

Monoisotopic Mass, Even Electron Ions
900 formula(e) evaluated with 5 results within limits (up to 50 best isotopic matches for each mass)
Elements Used:
C: 0-25 H: 0-1000 N: 0-10 O: 0-10 Na: 0-1

MJ-30-1 4 (0.087)

1: TOF MS ES+

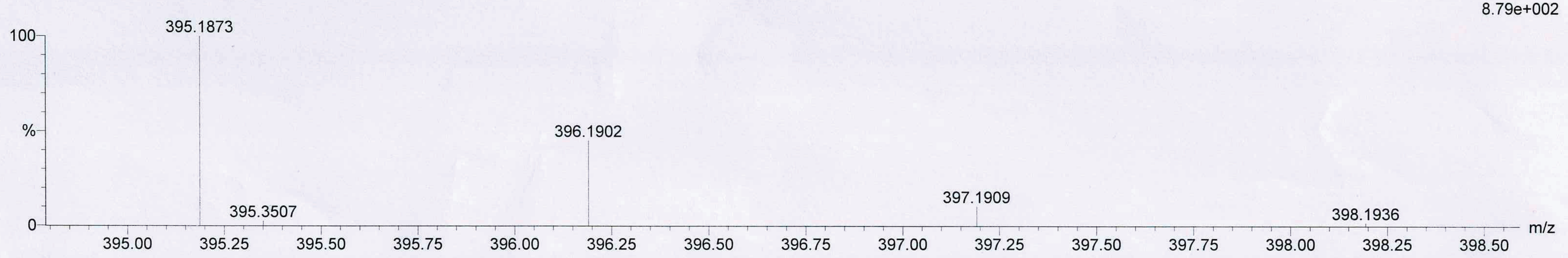

Minimum:				-1.5		
Maximum:		5.0	5.0	50.0		

Mass	Calc. Mass	mDa	PPM	DBE	i-FIT	Formula
395.1873	395.1872	0.1	0.3	16.5	32.0	C25 H23 N4 O
	395.1858	1.5	3.8	11.5	38.3	C24 H27 O5
	395.1890	-1.7	-4.3	3.5	90.9	C13 H27 N6 O8
	395.1880	-0.7	-1.8	5.5	95.3	C12 H24 N10 O4 Na
	395.1866	0.7	1.8	0.5	106.1	C11 H28 N6 O8 Na

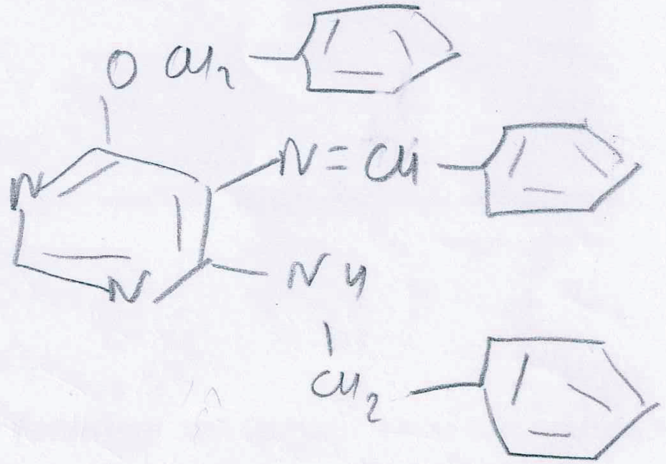

15. N5-benzylidene-6-(benzyloxy)-N4-isopropylpyrimidine-4,5-diamine (C-NMR)

8 Jul 2013

Acquisition Time (sec)	1.0486	***Comment***	new experiment	***Date***	Jul 3 2013
File Name	C:\Users\usuario\Documents\Espectros 2"Gen-Asier\13-15463_Benz-1\carbono				
Frequency (MHz)	125.68	***Nucleus***	13C	***Number of Transients***	3200
Original Points Count	32768	***Points Count***	32768	***Pulse Sequence***	s2pul
Solvent	CHLOROFORM-D			***Sweep Width (Hz)***	31250.00
Temperature (degree C)	25.000				

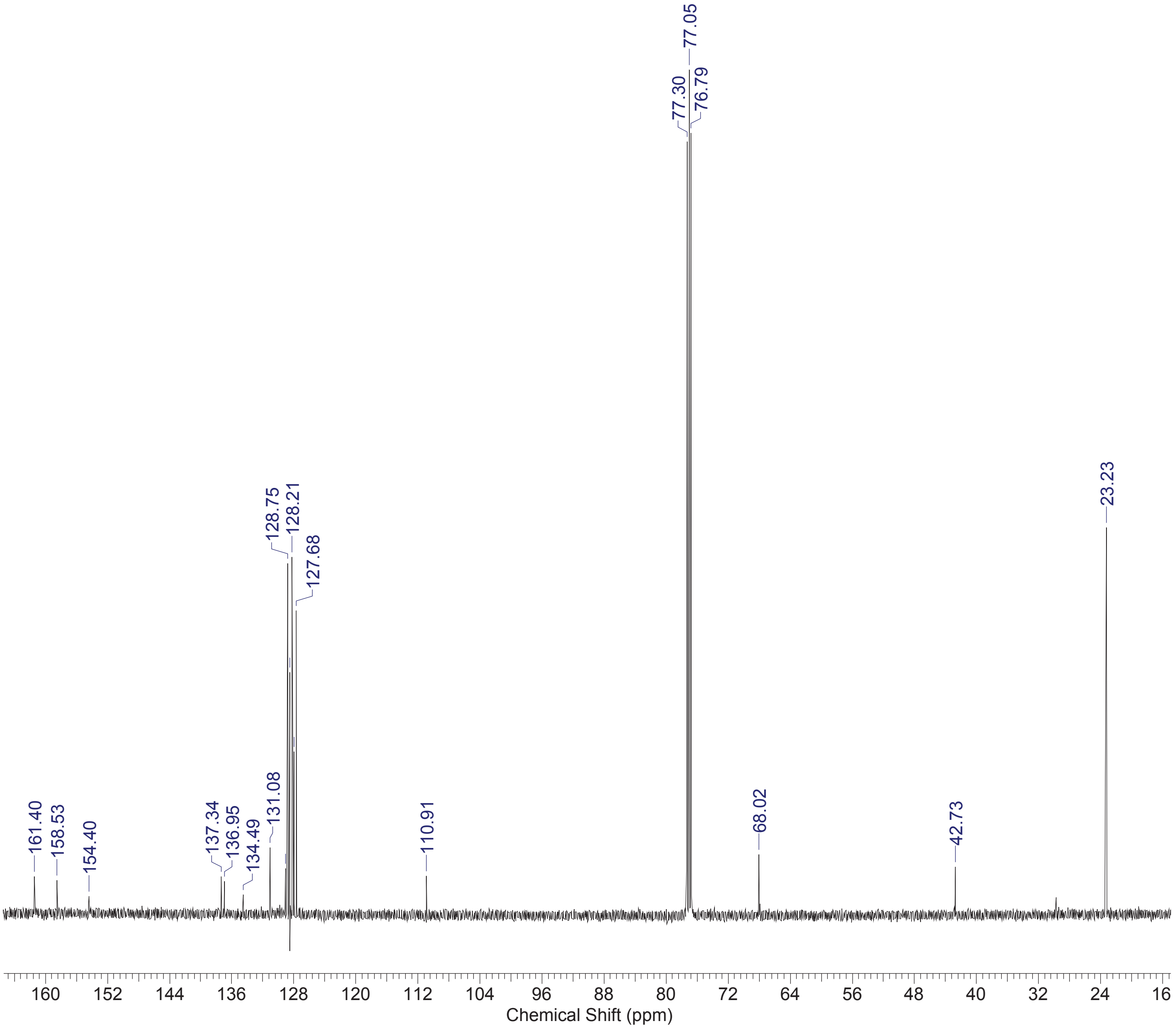

No.	(ppm)	(Hz)	Height	No.	(ppm)	(Hz)	Height
1	23.23	2919.2	0.4578	11	128.53	16154.6	0.2868
2	42.73	5371.1	0.0564	12	128.75	16182.3	0.4156
3	68.02	8548.9	0.0705	13	129.03	16217.6	0.0545
4	76.79	9651.3	0.9251	14	131.08	16474.1	0.0790
5	77.05	9683.8	1.0000	15	134.49	16903.3	0.0235
6	77.30	9715.2	0.9148	16	136.95	17212.3	0.0390
7	110.91	13939.2	0.0453	17	137.34	17260.9	0.0447
8	127.68	16047.8	0.3596	18	154.40	19405.8	0.0215
9	127.96	16082.2	0.1924	19	158.53	19924.6	0.0402
10	128.21	16113.6	0.4232	20	161.40	20285.1	0.0449

15. N5-benzylidene-6-(benzyloxy)-N4-isopropylpyrimidine-4,5-diamine (H-NMR)

3 Jul 2013

Acquisition Time (sec)	2.0447	***Date***	Jun 24 2013
File Name	C:\Users\usuario\Documents\Espectros 2"Gen-Asier\13-15463_Benz-1\PROTON_01		
Frequency (MHz)	499.79	***Nucleus***	1H
Number of Transients	8		
Original Points Count	16384	***Points Count***	16384
Pulse Sequence	s2pul		
Solvent	CHLOROFORM-D	***Sweep Width (Hz)***	8012.82
Temperature (degree C)	25.000		

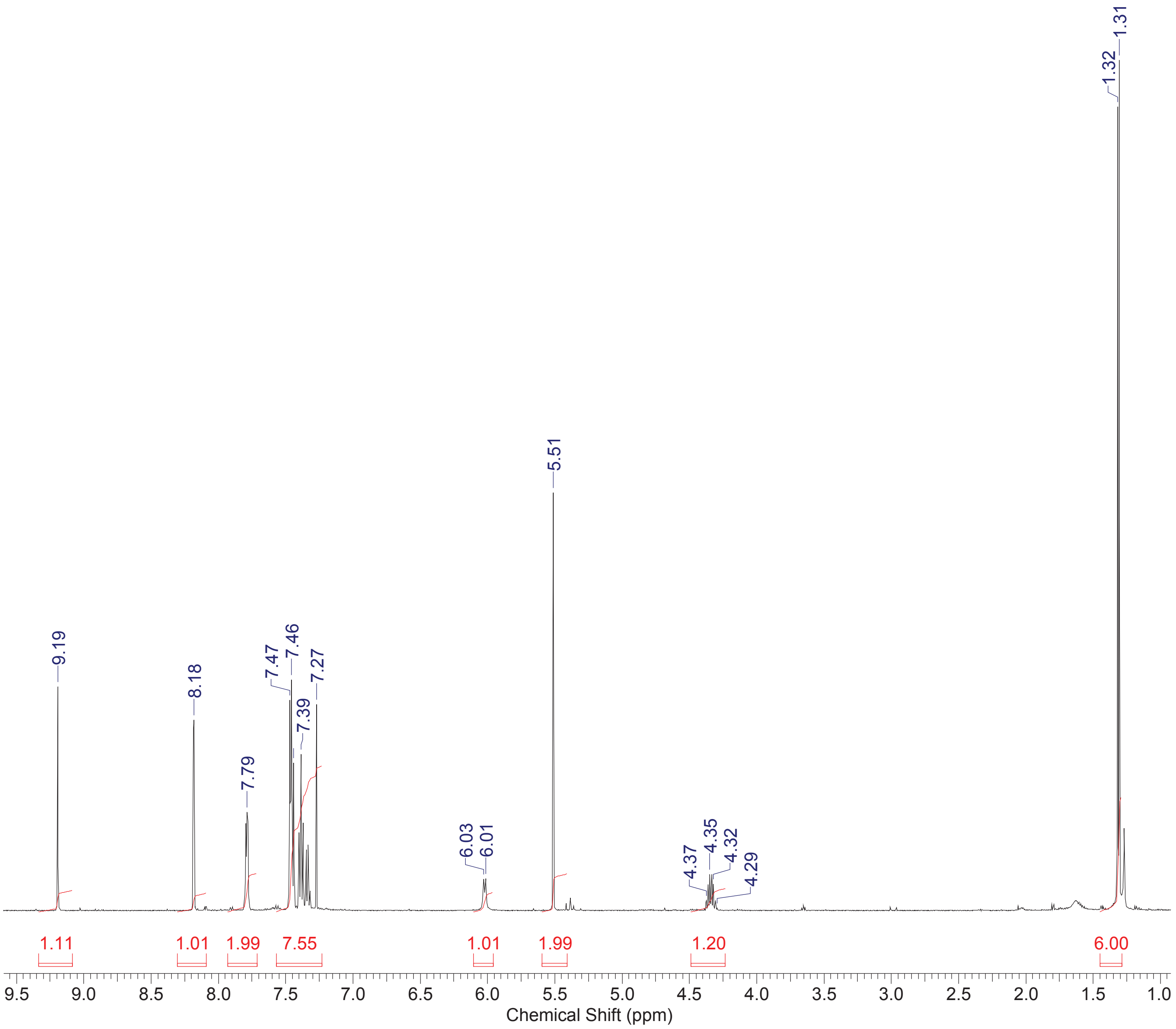

No.	(ppm)	(Hz)	Height
1	1.31	652.2	1.0000
2	1.32	659.1	0.9448
3	4.29	2145.9	0.0044
4	4.32	2159.1	0.0327
5	4.35	2173.8	0.0440
6	4.37	2186.5	0.0131
7	5.51	2754.9	0.4921
8	6.01	3005.8	0.0392
9	6.03	3013.6	0.0388
10	7.27	3634.2	0.2440
11	7.39	3691.5	0.1852
12	7.44	3718.4	0.1749
13	7.46	3727.2	0.2726
14	7.46	3730.6	0.2329
15	7.47	3733.5	0.2488
16	7.79	3892.0	0.1170
17	8.18	4089.6	0.2253
18	9.19	4594.8	0.2644

No.	(ppm)	Value	Absolute Value
1	[1.29 .. 1.45]	6.000	3.44717e+8
2	[4.23 .. 4.49]	1.202	6.90548e+7
3	[5.41 .. 5.60]	1.992	1.14457e+8
4	[5.96 .. 6.11]	1.009	5.79428e+7
5	[7.23 .. 7.57]	7.548	4.33660e+8
6	[7.71 .. 7.93]	1.991	1.14368e+8
7	[8.09 .. 8.31]	1.008	5.79299e+7
8	[9.08 .. 9.33]	1.110	6.37490e+7

15. N5-benzylidene-6-(benzyloxy)-N4-isopropylpyrimidine-4,5-diamine (H-NMR)

Elemental Composition Report

Single Mass Analysis

Tolerance = 10.0 PPM / DBE: min = -1.5, max = 50.0
Element prediction: Off
Number of isotope peaks used for i-FIT = 3

Monoisotopic Mass, Even Electron Ions
215 formula(e) evaluated with 3 results within limits (up to 50 best isotopic matches for each mass)
Elements Used:
C: 0-21 H: 0-100 N: 0-5 O: 0-5 Na: 0-1

02-1 4 (0.086)
1: TOF MS ES+

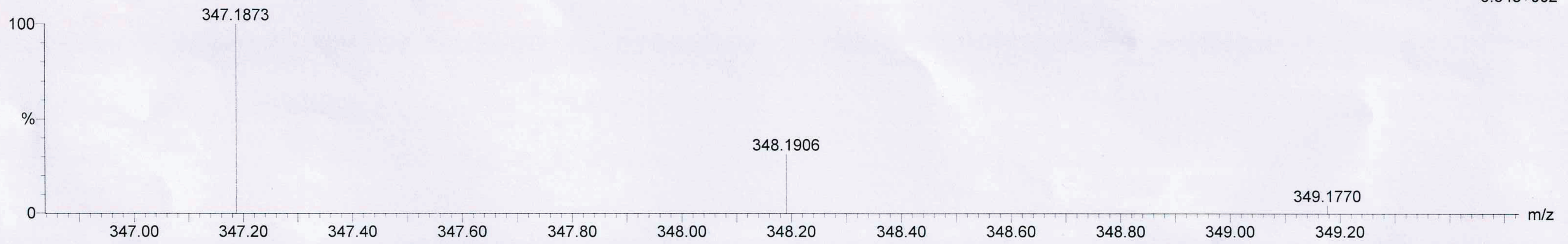

		mDa	PPM	DBE		
Minimum:				-1.5		
Maximum:		5.0	10.0	50.0		

Mass	Calc. Mass	mDa	PPM	DBE	i-FIT	Formula
347.1873	347.1872	0.1	0.3	12.5	3.2	C21 H23 N4 O
	347.1858	1.5	4.3	7.5	5.6	C20 H27 O5
	347.1848	2.5	7.2	9.5	6.4	C19 H24 N4 O Na

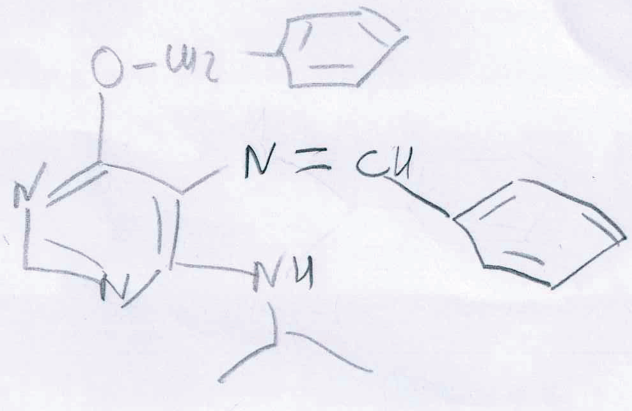

18. 5-amino-4-tert-butylamin-6-chloropyrimidine (MS)

Elemental Composition Report

Single Mass Analysis

Tolerance = 5.0 PPM / DBE: min = -1.5, max = 50.0
Element prediction: Off
Number of isotope peaks used for i-FIT = 3

Monoisotopic Mass, Even Electron Ions
960 formula(e) evaluated with 3 results within limits (up to 50 closest results for each mass)
Elements Used:
C: 0-17 H: 0-1000 N: 0-10 O: 0-10 Na: 0-1 S: 0-1 Cl: 0-1

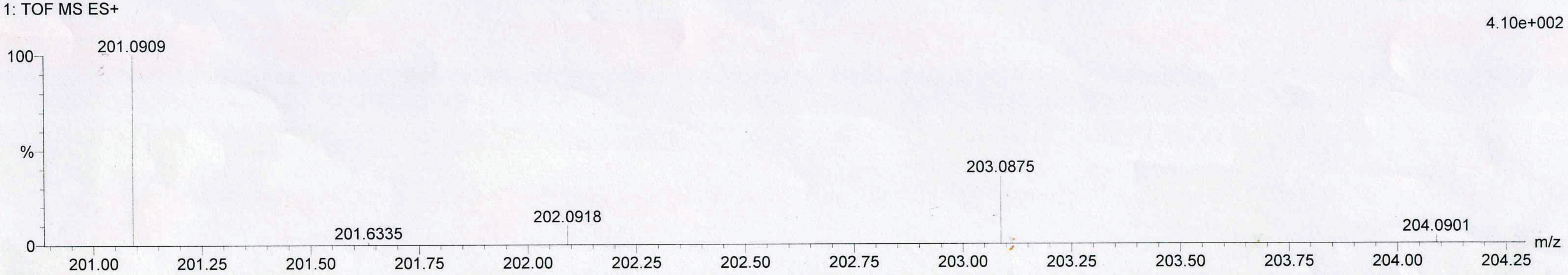

Mass	Calc. Mass	mDa	PPM	DBE	i-FIT	Formula
Minimum:				-1.5		
Maximum:		20.0	5.0	50.0		
201.0909	201.0907	0.2	1.0	3.5	0.6	C8 H14 N4 Cl
	201.0909	0.0	0.0	-1.5	52.4	C5 H17 N2 O4 S
	201.0916	-0.7	-3.5	7.5	72.7	C13 H13 O2

18. 5-amino-4-tert-butylamino-6-chloropyrimidine (H-NMR)

2 Jul 2013

Acquisition Time (sec)	2.0447	***Date***	Jun 19 2012		
File Name	C:\Users\usuario\Documents\Espectros Asier\Pre-TB-1			***Frequency (MHz)***	499.79
Nucleus	1H	***Number of Transients***	1	***Original Points Count***	16384
Points Count	16384	***Pulse Sequence***	s2pul	***Solvent***	CHLOROFORM-D
Sweep Width (Hz)	8012.82	***Temperature (degree C)***	25.000		

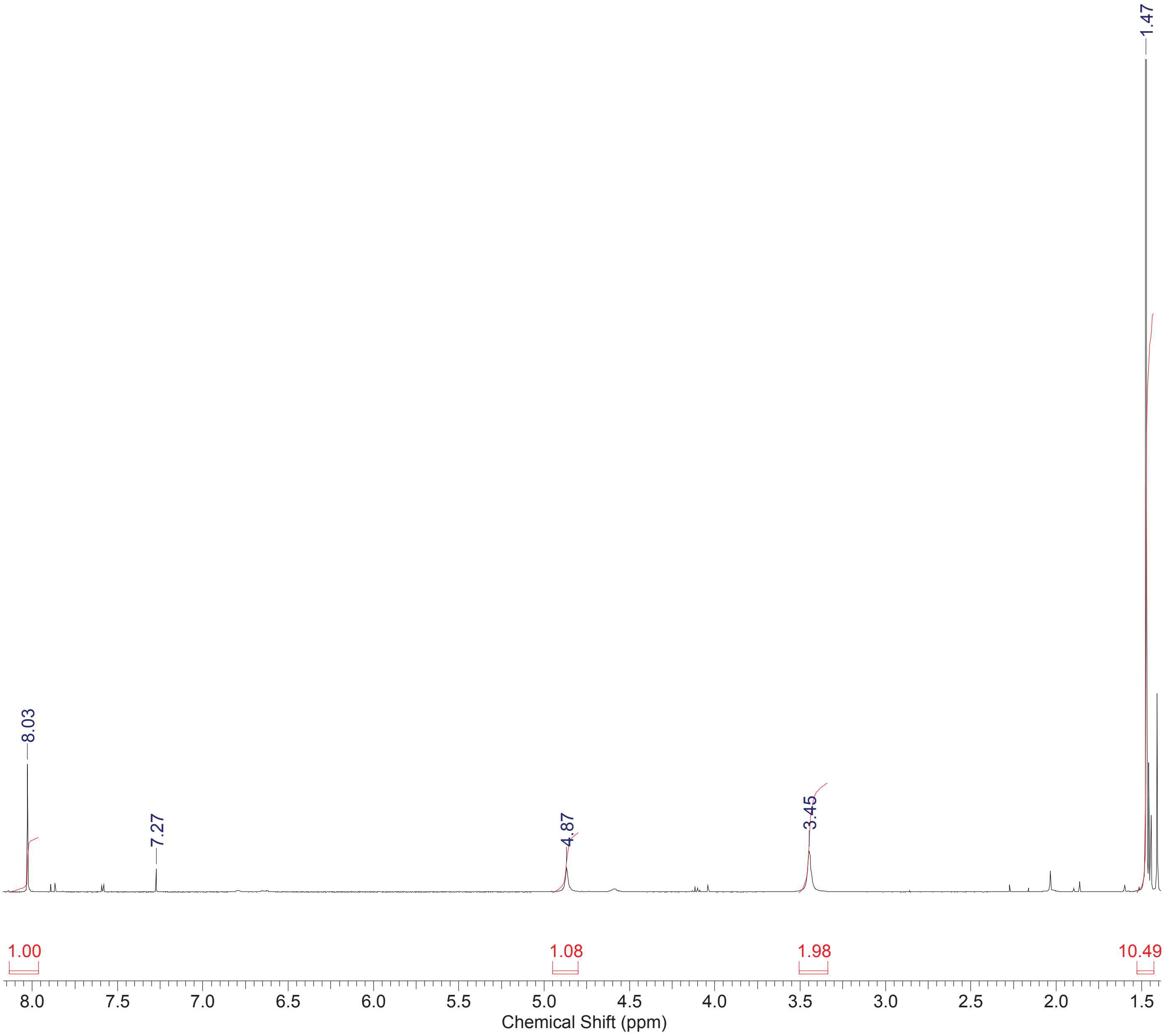

No.	(ppm)	(Hz)	Height
1	1.47	736.5	1.0000
2	3.45	1722.5	0.0217
3	4.87	2433.1	0.0131
4	7.27	3635.3	0.0125
5	8.03	4011.9	0.0669

No.	(ppm)	Value	Absolute Value
1	[1.43 .. 1.53]	10.486	1.68216e+8
2	[3.34 .. 3.51]	1.981	3.17831e+7
3	[4.80 .. 4.95]	1.083	1.73672e+7
4	[7.96 .. 8.14]	1.000	1.60426e+7

9. 6-Benzyloxy-9-isopropyl-8-methyl-9H-purine (MS)

Elemental Composition Report

Single Mass Analysis

Tolerance = 5.0 mDa / DBE: min = -1.5, max = 50.0
Element prediction: Off
Number of isotope peaks used for i-FIT = 3

Monoisotopic Mass, Even Electron Ions
44 formula(e) evaluated with 2 results within limits (up to 50 closest results for each mass)
Elements Used:
C: 0-16 H: 0-1000 N: 0-5 O: 0-1 Na: 0-1

ASIMJ-5 12-04 9 (0.212)

1: TOF MS ES+

6.64e+002

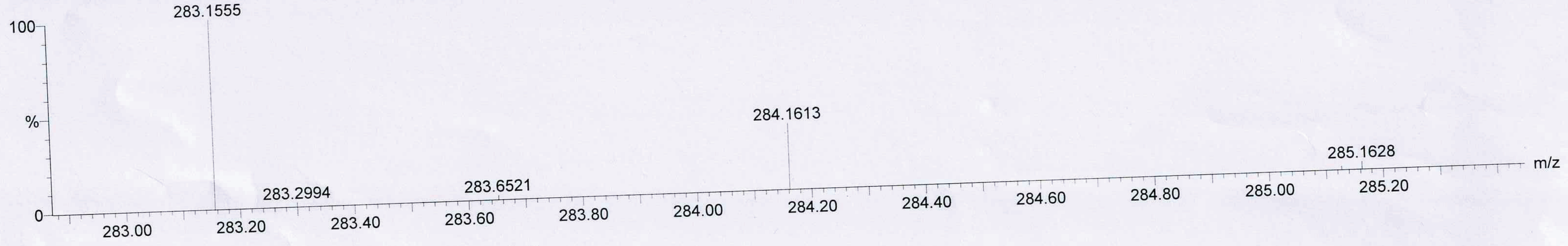

Minimum:				-1.5		
Maximum:		5.0	10.0	50.0		
Mass	Calc. Mass	mDa	PPM	DBE	i-FIT	Formula
283.1555	283.1559	-0.4	-1.4	9.5	23.1	C16 H19 N4 O
	283.1535	2.0	7.1	6.5	31.6	C14 H20 N4 O Na

A1, 9.   6-Benzyloxy-9-isopropyl-8-methyl-9H-purine (C-NMR)

2 Jul 2013

Acquisition Time (sec)	1.0486	***Date***	Feb 22 2012		
File Name	C:\Users\usuario\Documents\Espectros Asier\RMN-158-12_ASIMJ-5\carbono				
Frequency (MHz)	125.68	***Nucleus***	13C	***Number of Transients***	640
Original Points Count	32768	***Points Count***	32768	***Pulse Sequence***	s2pul
Solvent	CHLOROFORM-D			***Sweep Width (Hz)***	31250.00
Temperature (degree C)	25.000				

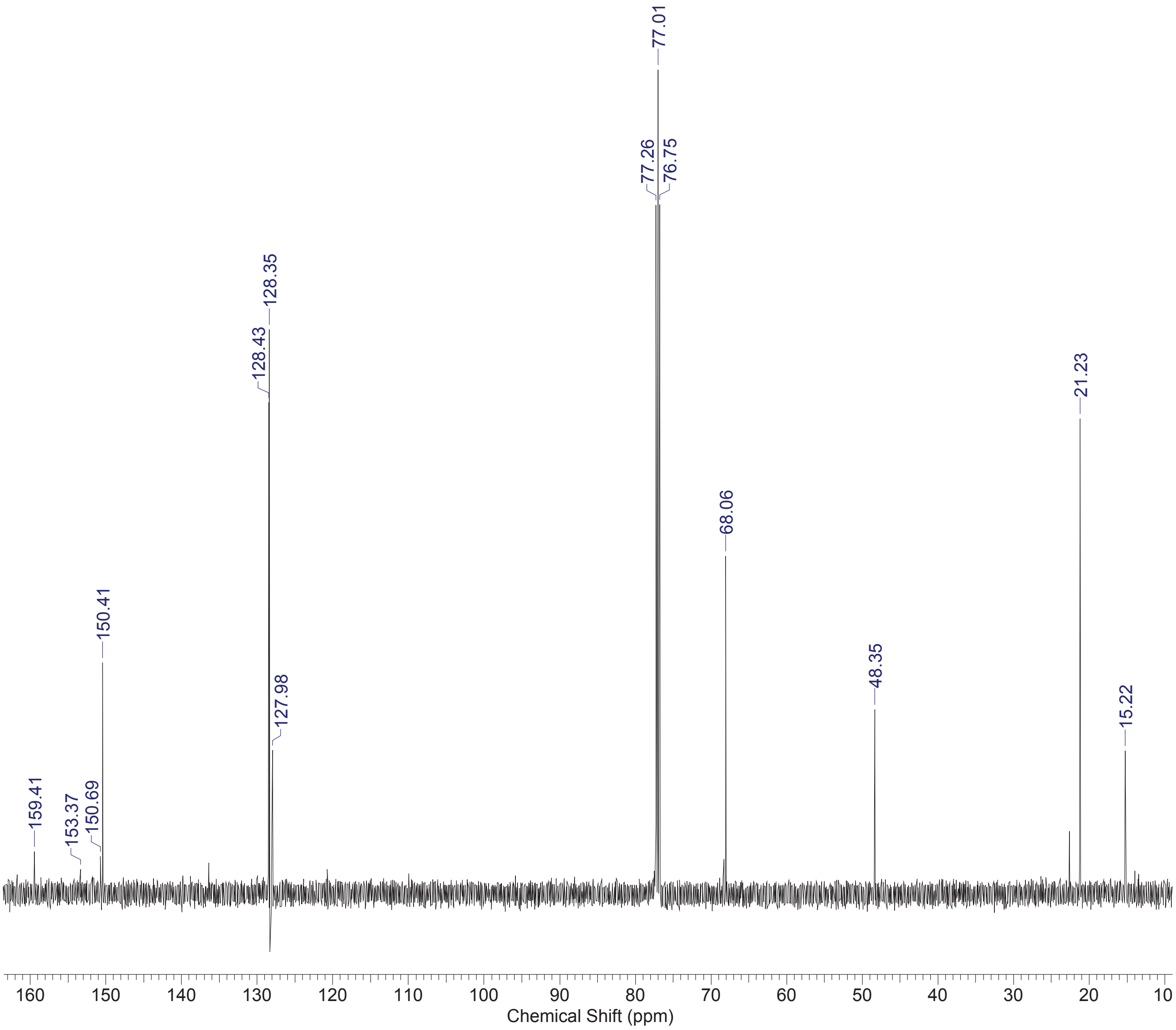

No.	(ppm)	(Hz)	Height
1	15.22	1913.4	0.1727
2	21.23	2667.8	0.5761
3	48.35	6077.3	0.2229
4	68.06	8554.1	0.4096
5	76.75	9646.1	0.8368
6	77.01	9678.5	1.0000
7	77.26	9710.0	0.8358
8	127.98	16085.5	0.1738
9	128.35	16132.2	0.6845
10	128.43	16141.7	0.5959
11	150.41	18903.7	0.2798
12	150.69	18939.9	0.0444
13	153.37	19276.6	0.0292
14	159.41	20035.7	0.0507

A1, 9. 6-Benzyloxy-9-isopropyl-8-methyl-9H-purine (DEPT)

2 Jul 2013

Acquisition Time (sec)	1.0486	***Comment***	new experiment	***Date***	Feb 22 2012
File Name	C:\Users\usuario\Documents\Espectros Asier\RMN-158-12_ASIMJ-5\dept				
Frequency (MHz)	125.68	***Nucleus***	13C	***Number of Transients***	2400
Original Points Count	32768	***Points Count***	32768	***Pulse Sequence***	DEPT135
Solvent	CHLOROFORM-D			***Sweep Width (Hz)***	31250.00
Temperature (degree C)	25.000				

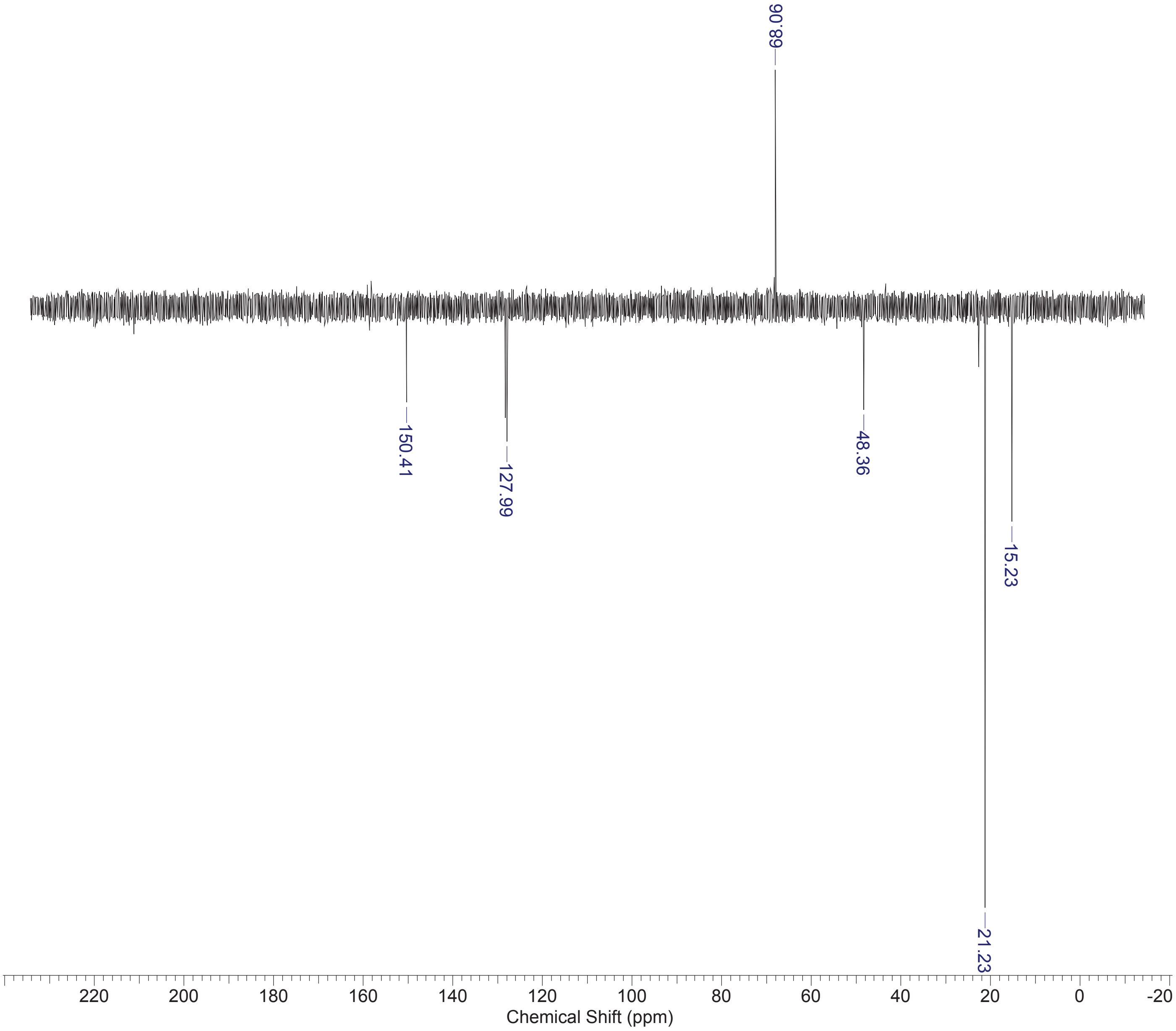

No.	(ppm)	(Hz)	Height
1	15.23	1914.4	-0.9040
2	21.23	2668.8	-2.5316
3	48.36	6078.3	-0.4334
4	68.06	8554.1	1.0000
5	127.99	16086.4	-0.5665
6	150.41	18903.7	-0.4006

A1,9. 6-Benzyloxy-9-isopropyl-8-methyl-9H-purine (H-NMR)

2 Jul 2013

Acquisition Time (sec)	1.9923	***Date***	Feb 22 2012		
File Name	C:\Users\usuario\Documents\Espectros Asier\RMN-158-12_ASIMJ-5\proton				
Frequency (MHz)	499.79	***Nucleus***	1H	***Number of Transients***	1
Original Points Count	15964	***Points Count***	16384	***Pulse Sequence***	s2pul
Solvent	CHLOROFORM-D			***Sweep Width (Hz)***	8012.82
Temperature (degree C)	25.000				

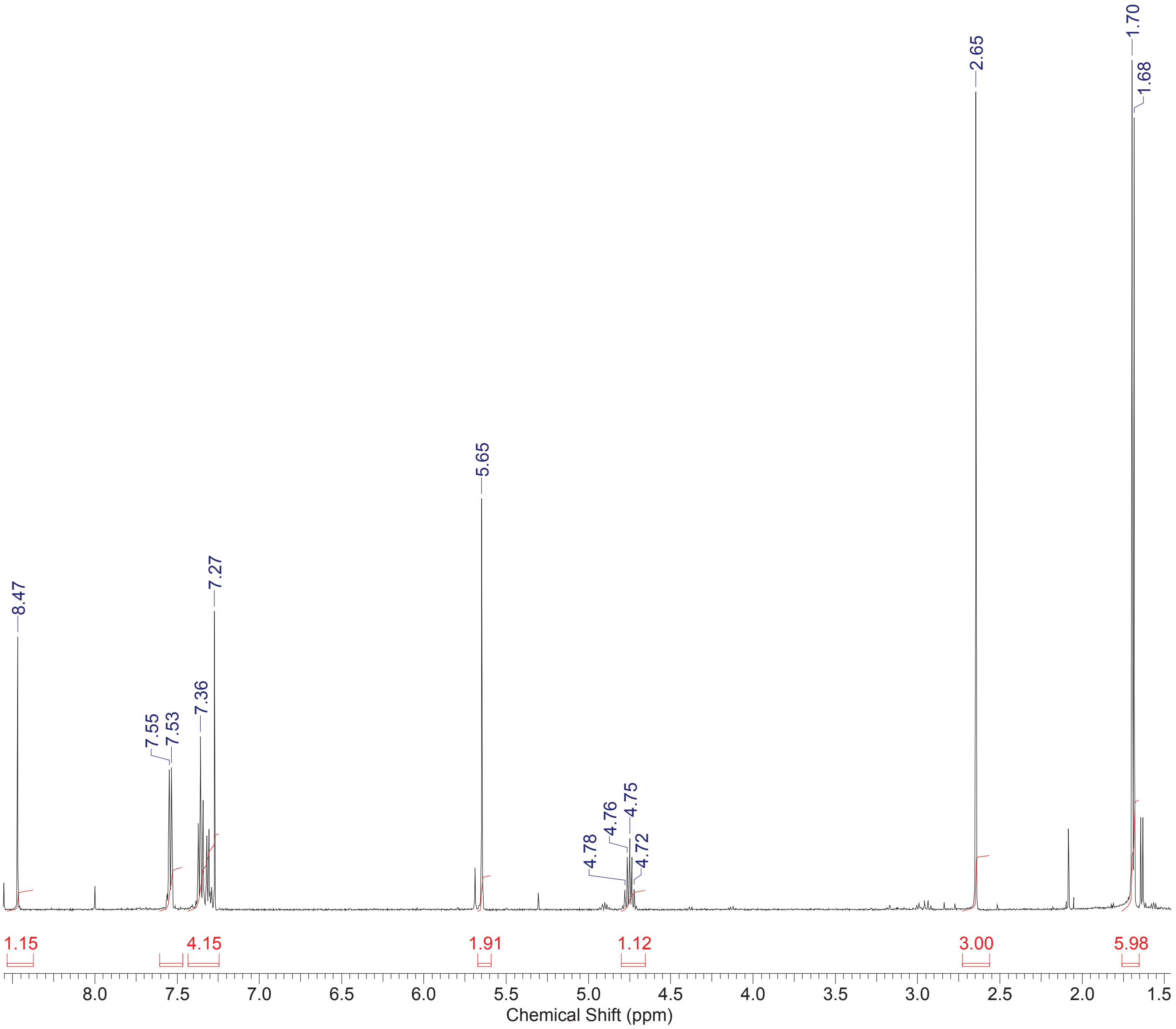

No.	(ppm)	(Hz)	Height
1	1.68	841.9	0.9325
2	1.70	848.7	1.0000
3	2.65	1322.6	0.9626
4	4.72	2360.5	0.0255
5	4.75	2374.2	0.0857
6	4.76	2381.0	0.0634
7	4.78	2387.9	0.0253
8	5.65	2823.2	0.4849
9	7.27	3634.6	0.3526
10	7.36	3677.1	0.2053
11	7.53	3765.2	0.1687
12	7.55	3772.0	0.1659
13	8.47	4232.7	0.3220

No.	(ppm)	Value	Absolute Value
1	[1.66 .. 1.76]	5.985	2.44828e+6
2	[2.56 .. 2.73]	3.000	1.22724e+6
3	[4.65 .. 4.80]	1.124	4.59616e+5
4	[5.59 .. 5.67]	1.908	7.80710e+5
5	[7.25 .. 7.43]	4.154	1.69922e+6
6	[7.47 .. 7.61]	2.360	9.65603e+5
7	[8.37 .. 8.53]	1.147	4.69248e+5

A1. 6-(Benzyloxy)-9-isopropyl-8-methyl-9H-purine (MS)

Elemental Composition Report

Single Mass Analysis

Tolerance = 5.0 PPM / DBE: min = -1.5, max = 50.0
Element prediction: Off
Number of isotope peaks used for i-FIT = 3

Monoisotopic Mass, Even Electron Ions
525 formula(e) evaluated with 4 results within limits (up to 50 best isotopic matches for each mass)
Elements Used:
C: 0-22 H: 0-1000 N: 0-5 O: 0-6 F: 0-3

12/3293

ASIMJ-5 9 (0.217)

1: TOF MS ES+
9.28e+001

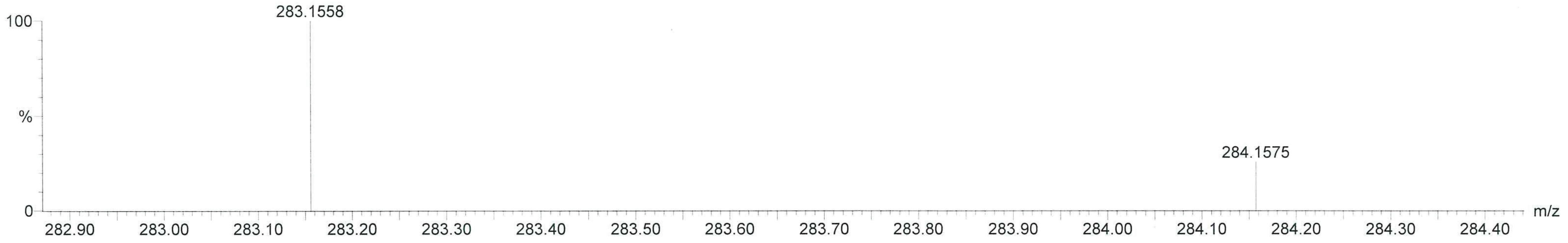

Minimum:				-1.5		
Maximum:		20.0	5.0	50.0		

Mass	Calc. Mass	mDa	PPM	DBE	i-FIT	Formula
283.1558	283.1559	-0.1	-0.4	9.5	n/a	C16 H19 N4 O
	283.1570	-1.2	-4.2	5.5	n/a	C13 H20 N4 O2 F
	283.1545	1.3	4.6	4.5	n/a	C15 H23 O5
	283.1557	0.1	0.4	0.5	n/a	C12 H24 O6 F

A2. 6-Ethoxy-8-ethyl-9-isopropyl-9H-purine (H-NMR)

11 Jul 2012

Acquisition Time (sec)	2.0484	***Comment***	ASI-MJ-2	***Date***	Feb 6 2012
File Name	C:\Users\usuario\Documents\Espectros Asier\1H-ASI-MJ-2				
Frequency (MHz)	300.20	***Nucleus***	1H	***Number of Transients***	64
Original Points Count	7379	***Points Count***	8192	***Pulse Sequence***	s2pul
Solvent	CHLOROFORM-D			***Sweep Width (Hz)***	3602.31
Temperature (degree C)	30.000				

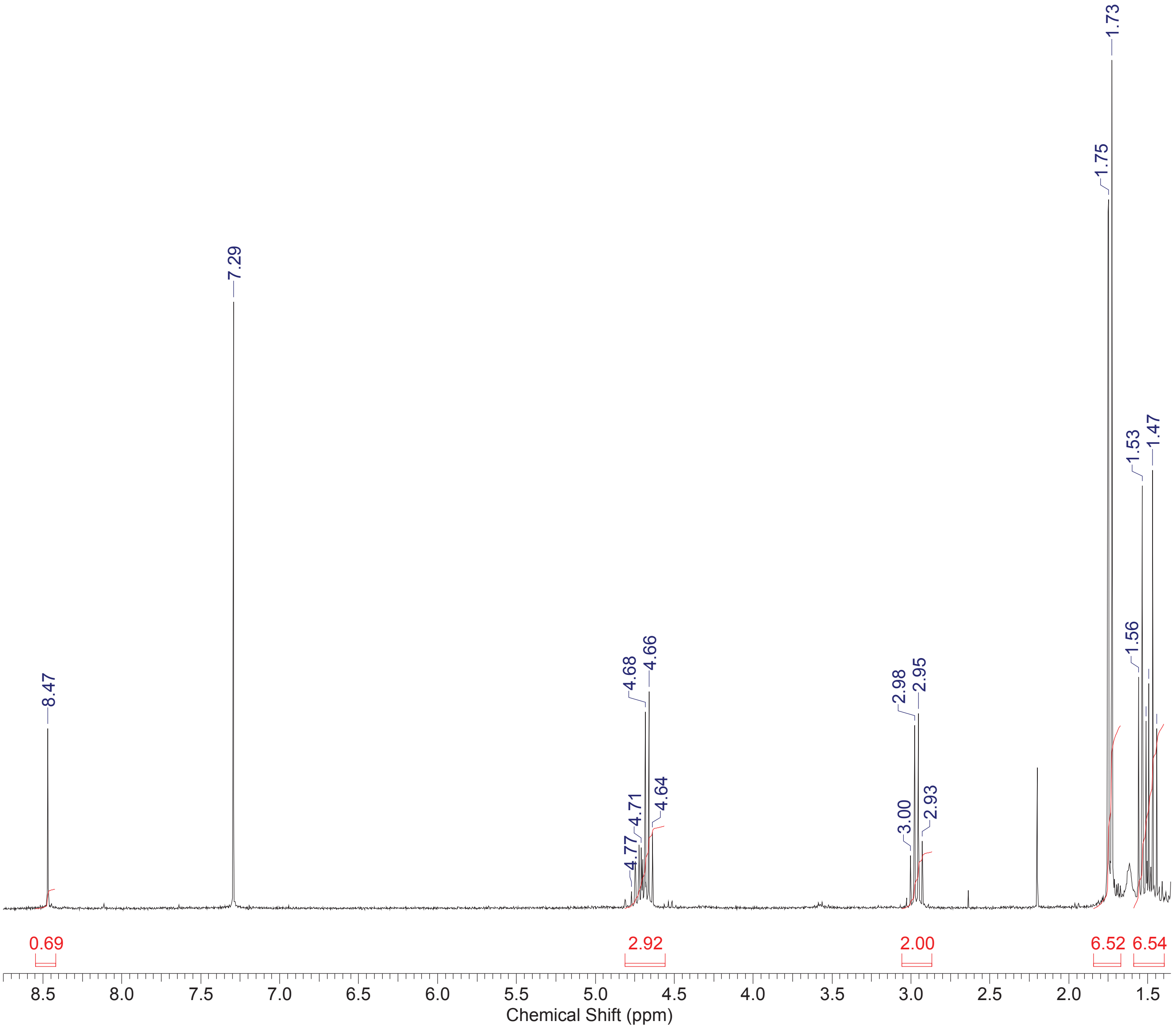

No.	(ppm)	(Hz)	Height
1	1.44	433.4	0.2121
2	1.47	440.9	0.5167
3	1.49	448.4	0.2655
4	1.51	453.2	0.2214
5	1.53	460.7	0.4987
6	1.56	467.7	0.2730
7	1.73	518.7	1.0000
8	1.75	525.3	0.8356
9	2.93	878.9	0.0799
10	2.95	886.4	0.2303

No.	(ppm)	(Hz)	Height
11	2.98	893.9	0.2163
12	3.00	901.3	0.0624
13	4.64	1392.1	0.0903
14	4.66	1399.2	0.2558
15	4.68	1406.2	0.2320
16	4.71	1413.7	0.0724
17	4.77	1432.2	0.0207
18	7.29	2189.5	0.7151
19	8.47	2542.2	0.2124

No.	(ppm)	Value	Absolute Value
1	[1.39 .. 1.59]	6.536	2.20237e+8
2	[1.67 .. 1.84]	6.517	2.19591e+8
3	[2.87 .. 3.06]	2.000	6.73878e+7
4	[4.56 .. 4.81]	2.918	9.83286e+7
5	[8.42 .. 8.55]	0.693	2.33543e+7

A2. 6-Ethoxy-8-ethyl-9-isopropyl-9H-purine (MS)

Elemental Composition Report

Single Mass Analysis

Tolerance = 5.0 PPM / DBE: min = -1.5, max = 50.0
Element prediction: Off
Number of isotope peaks used for i-FIT = 3

Monoisotopic Mass, Even Electron Ions
427 formula(e) evaluated with 2 results within limits (up to 50 best isotopic matches for each mass)
Elements Used:
C: 0-22 H: 0-1000 N: 0-5 O: 0-6 F: 0-3

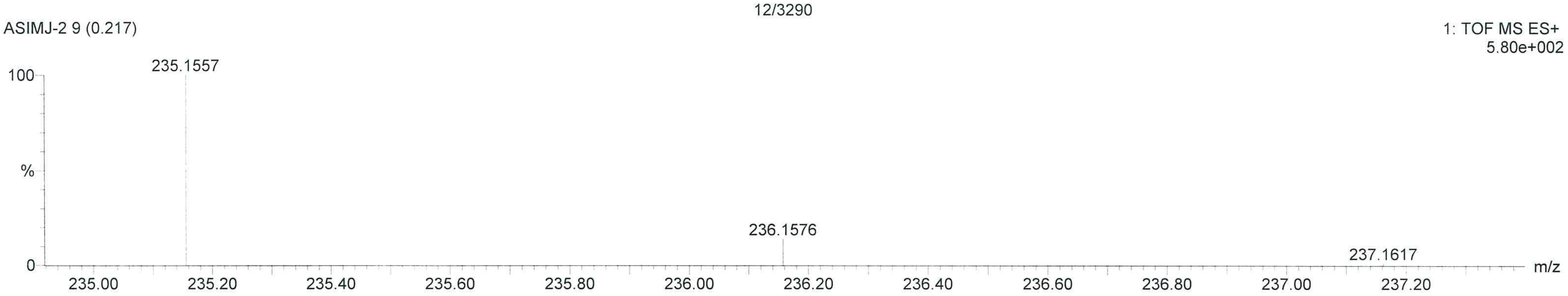

Minimum:				-1.5		
Maximum:		20.0	5.0	50.0		
Mass	Calc. Mass	mDa	PPM	DBE	i-FIT	Formula
235.1557	235.1559	-0.2	-0.9	5.5	0.3	C12 H19 N4 O
	235.1545	1.2	5.1	0.5	1.0	C11 H23 O5

A4. 6-Ethoxy-9-isopropyl-9H-purine (DEPT)

11 Jul 2012

Acquisition Time (sec)	1.0486	***Date***	Feb 4 2012		
File Name	C:\Users\usuario\Documents\Espectros Asier\RMN-99-12_ASIMJ-1\DEPT_01				
Frequency (MHz)	125.68	***Nucleus***	13C	***Number of Transients***	1200
Original Points Count	32768	***Points Count***	32768	***Pulse Sequence***	DEPT135
Solvent	CHLOROFORM-D			***Sweep Width (Hz)***	31250.00
Temperature (degree C)	25.000				

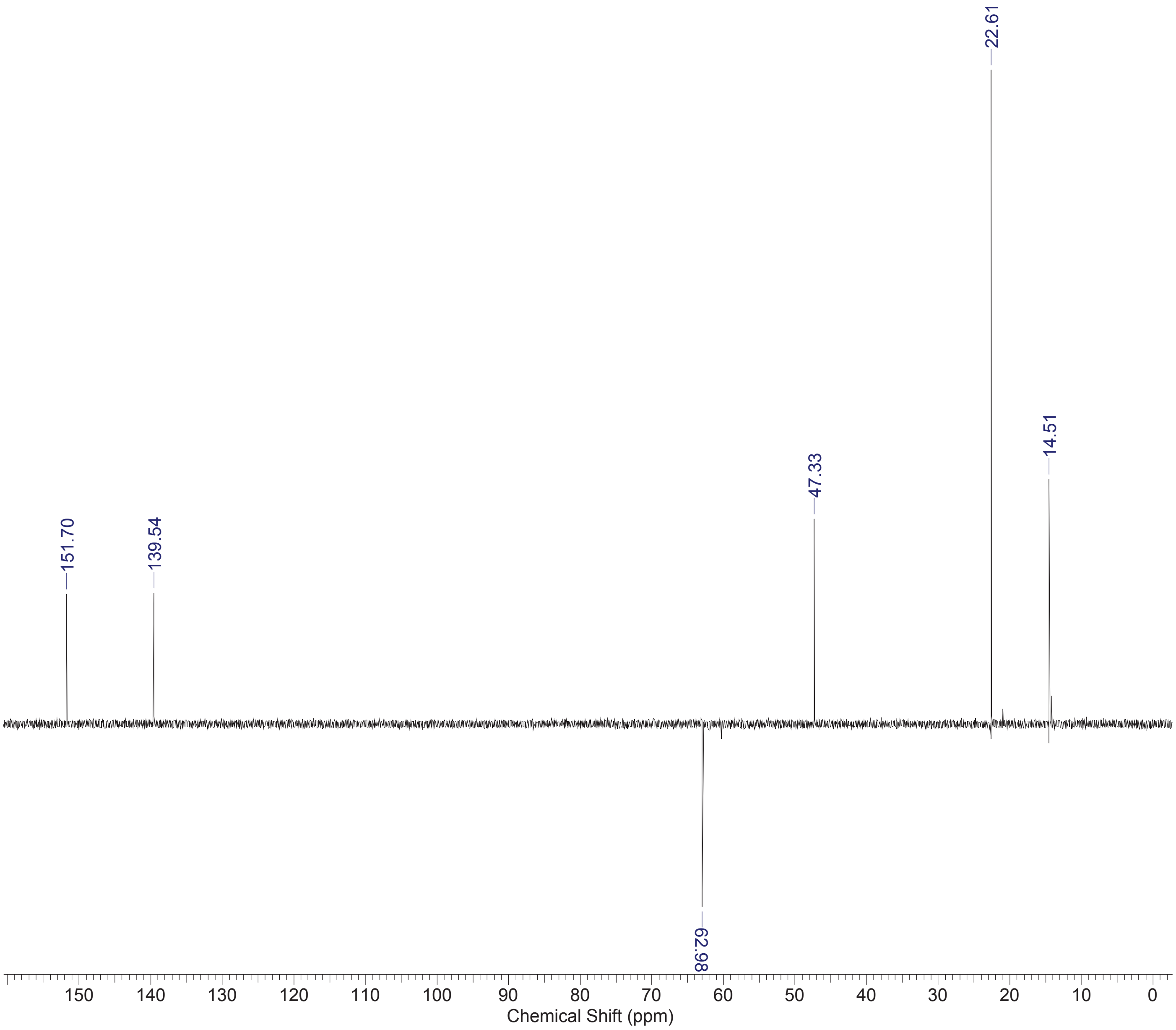

No.	(ppm)	(Hz)	Height
1	14.51	1823.8	0.3743
2	22.61	2841.4	1.0000
3	47.33	5948.6	0.3133
4	62.98	7915.1	-0.2795
5	139.54	17538.0	0.2003
6	151.70	19066.8	0.1987

A4. 6-Ethoxy-9-isopropyl-9H-purine (H-NMR)

11 Jul 2012

Acquisition Time (sec)	2.0447	***Date***	Feb 4 2012		
File Name	C:\Users\usuario\Documents\Espectros Asier\RMN-99-12_ASIMJ-1\PROTON_01				
Frequency (MHz)	499.79	***Nucleus***	1H	***Number of Transients***	8
Original Points Count	16384	***Points Count***	16384	***Pulse Sequence***	s2pul
Solvent	CHLOROFORM-D			***Sweep Width (Hz)***	8012.82
Temperature (degree C)	25.000				

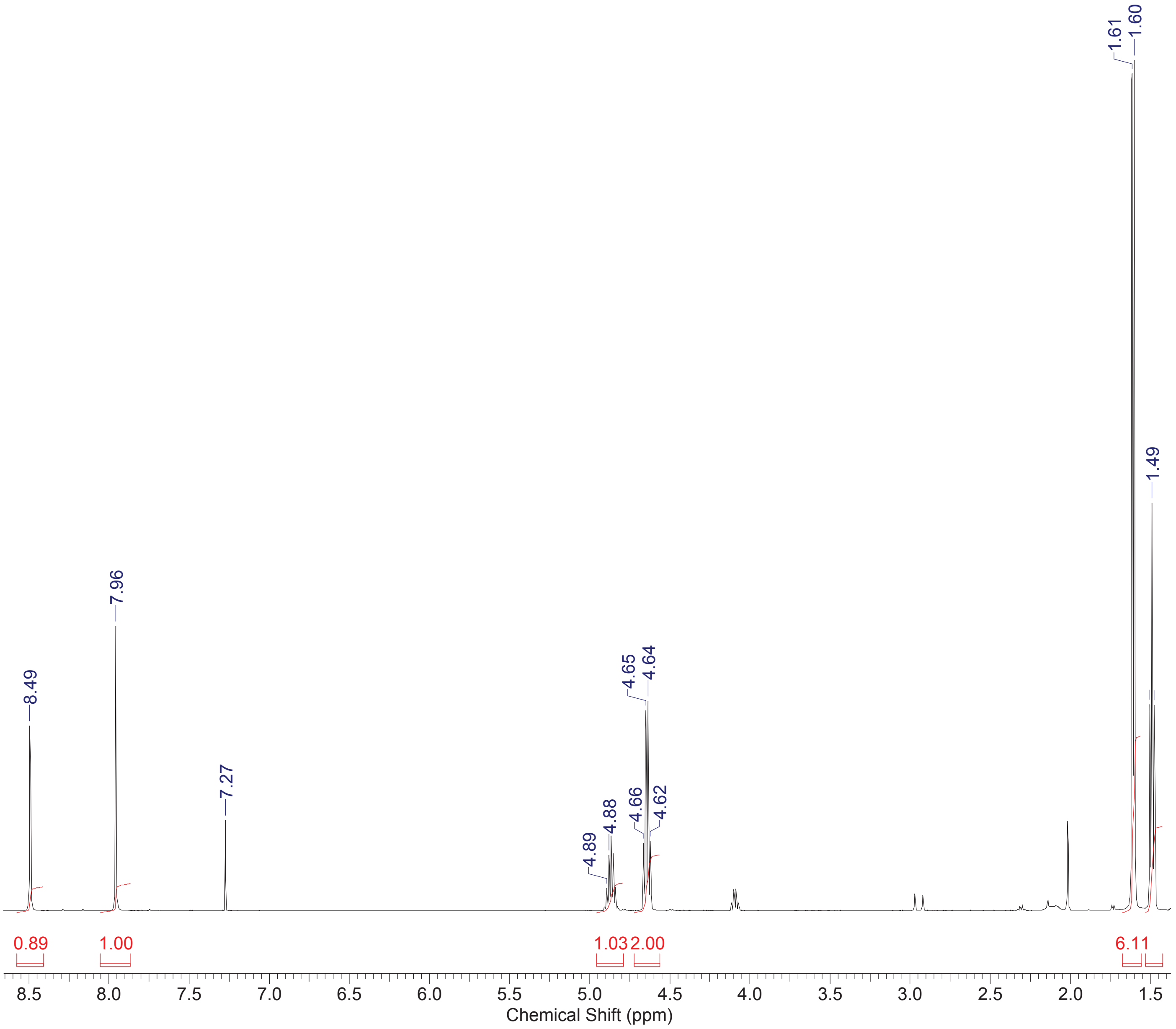

No.	(ppm)	(Hz)	Height
1	1.48	738.3	0.2434
2	1.49	745.2	0.4809
3	1.51	752.5	0.2440
4	1.60	800.9	1.0000
5	1.61	806.8	0.9840
6	4.62	2309.8	0.0837
7	4.64	2317.1	0.2483
8	4.65	2323.5	0.2373
9	4.66	2331.3	0.0814
10	4.88	2439.4	0.0676
11	4.89	2446.2	0.0283
12	7.27	3635.2	0.1085
13	7.96	3977.6	0.3363
14	8.49	4245.6	0.2190

No.	(ppm)	Value	Absolute Value
1	[1.42 .. 1.53]	2.979	1.74678e+8
2	[1.56 .. 1.67]	6.113	3.58379e+8
3	[4.56 .. 4.72]	2.000	1.17260e+8
4	[4.79 .. 4.96]	1.030	6.03864e+7
5	[7.87 .. 8.06]	1.002	5.87716e+7
6	[8.41 .. 8.58]	0.894	5.24249e+7

A4. 6-Ethoxy-9-isopropyl-9H-purine (MS)

Elemental Composition Report

Single Mass Analysis

Tolerance = 5.0 PPM / DBE: min = -1.5, max = 50.0
Element prediction: Off
Number of isotope peaks used for i-FIT = 3

Monoisotopic Mass, Even Electron Ions
369 formula(e) evaluated with 1 results within limits (up to 50 best isotopic matches for each mass)
Elements Used:
C: 0-22 H: 0-1000 N: 0-5 O: 0-6 F: 0-3

12/3289

ASIMJ-1 40 (0.885)

1: TOF MS ES+
1.90e+002

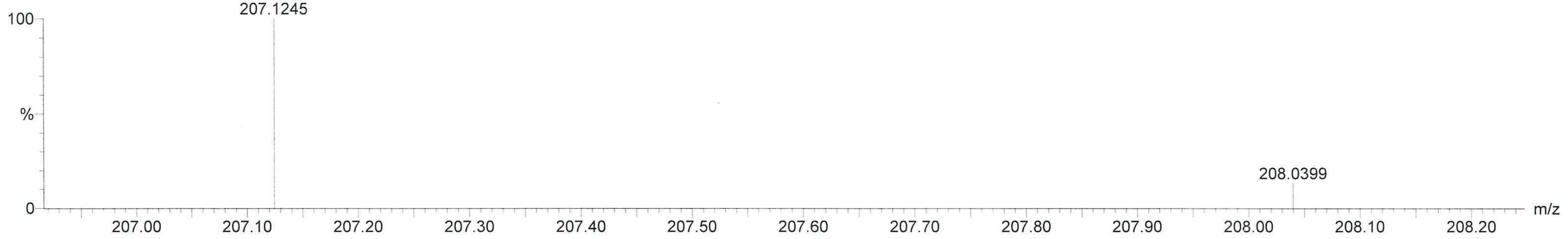

Minimum:				-1.5		
Maximum:		20.0	5.0	50.0		
Mass	Calc. Mass	mDa	PPM	DBE	i-FIT	Formula
207.1245	207.1246	-0.1	-0.5	5.5	n/a	C10 H15 N4 O

A4. 6-Ethoxy-9-isopropyl-9H-purine (C-NMR)

11 Jul 2012

Acquisition Time (sec)	1.0486	***Date***	Feb 4 2012		
File Name	C:\Users\usuario\Documents\Espectros Asier\RMN-99-12_ASIMJ-1\CARBON_01				
Frequency (MHz)	125.68	***Nucleus***	13C	***Number of Transients***	3200
Original Points Count	32768	***Points Count***	32768	***Pulse Sequence***	s2pul
Solvent	CHLOROFORM-D			***Sweep Width (Hz)***	31250.00
Temperature (degree C)	25.000				

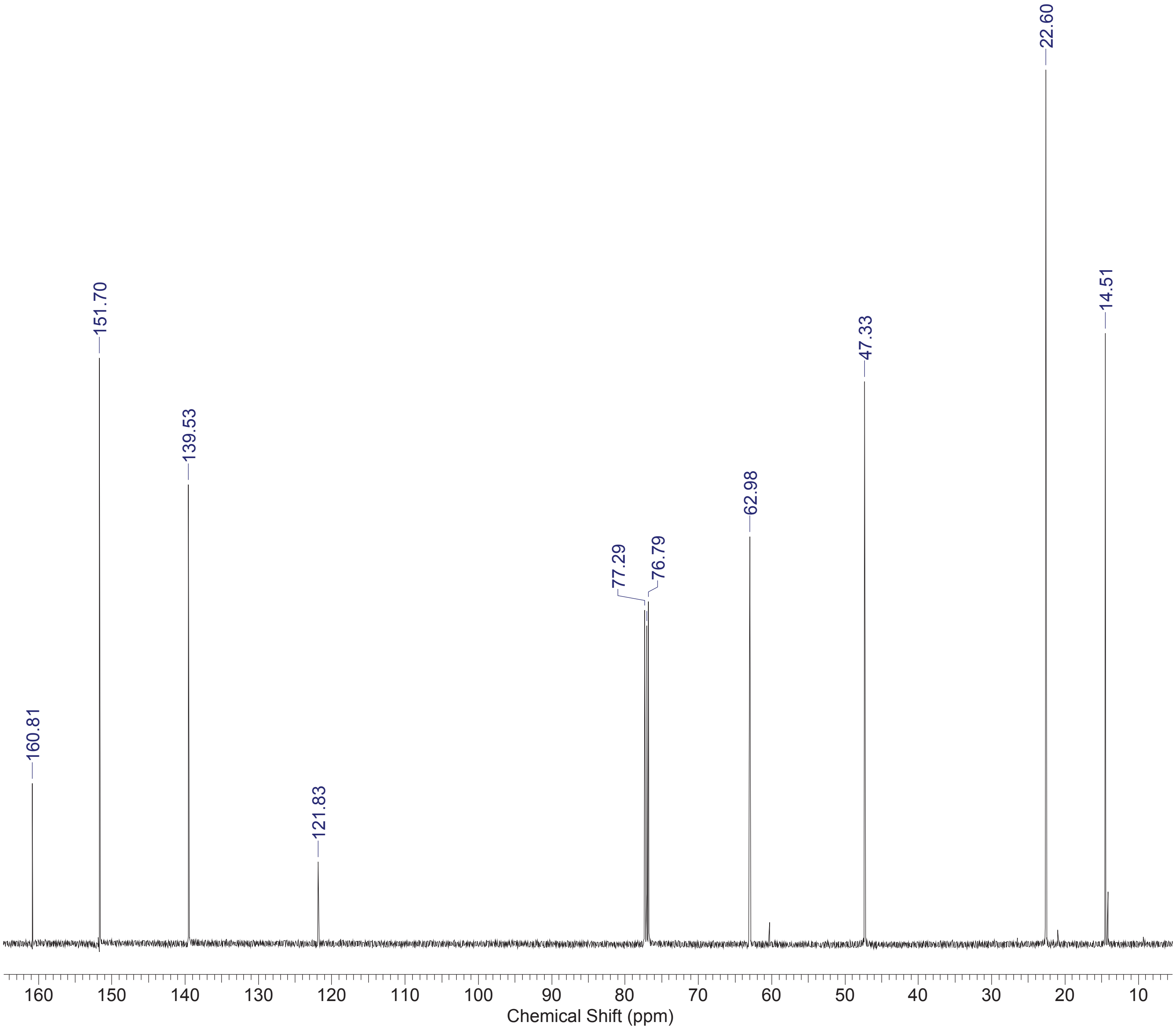

No.	(ppm)	(Hz)	Height
1	14.51	1823.8	0.6984
2	22.60	2840.4	1.0000
3	47.33	5948.6	0.6432
4	62.98	7915.1	0.4655
5	76.79	9650.8	0.3913
6	77.04	9683.3	0.3638
7	77.29	9714.7	0.3814
8	121.83	15312.0	0.0933
9	139.53	17537.0	0.5250
10	151.70	19066.8	0.6704
11	160.81	20211.2	0.1830

A5.   6-(Benzyloxy)-9-isopropyl-9H-purine (C-NMR)

12 Jul 2012

Acquisition Time (sec)	1.0486	***Date***	Feb 20 2012		
File Name	C:\Users\usuario\Documents\Espectros Asier\RMN-145-12_ASIMJ-4\carbono				
Frequency (MHz)	125.68	***Nucleus***	13C	***Number of Transients***	32000
Original Points Count	32768	***Points Count***	32768	***Pulse Sequence***	s2pul
Solvent	CHLOROFORM-D			***Sweep Width (Hz)***	31250.00
Temperature (degree C)	25.000				

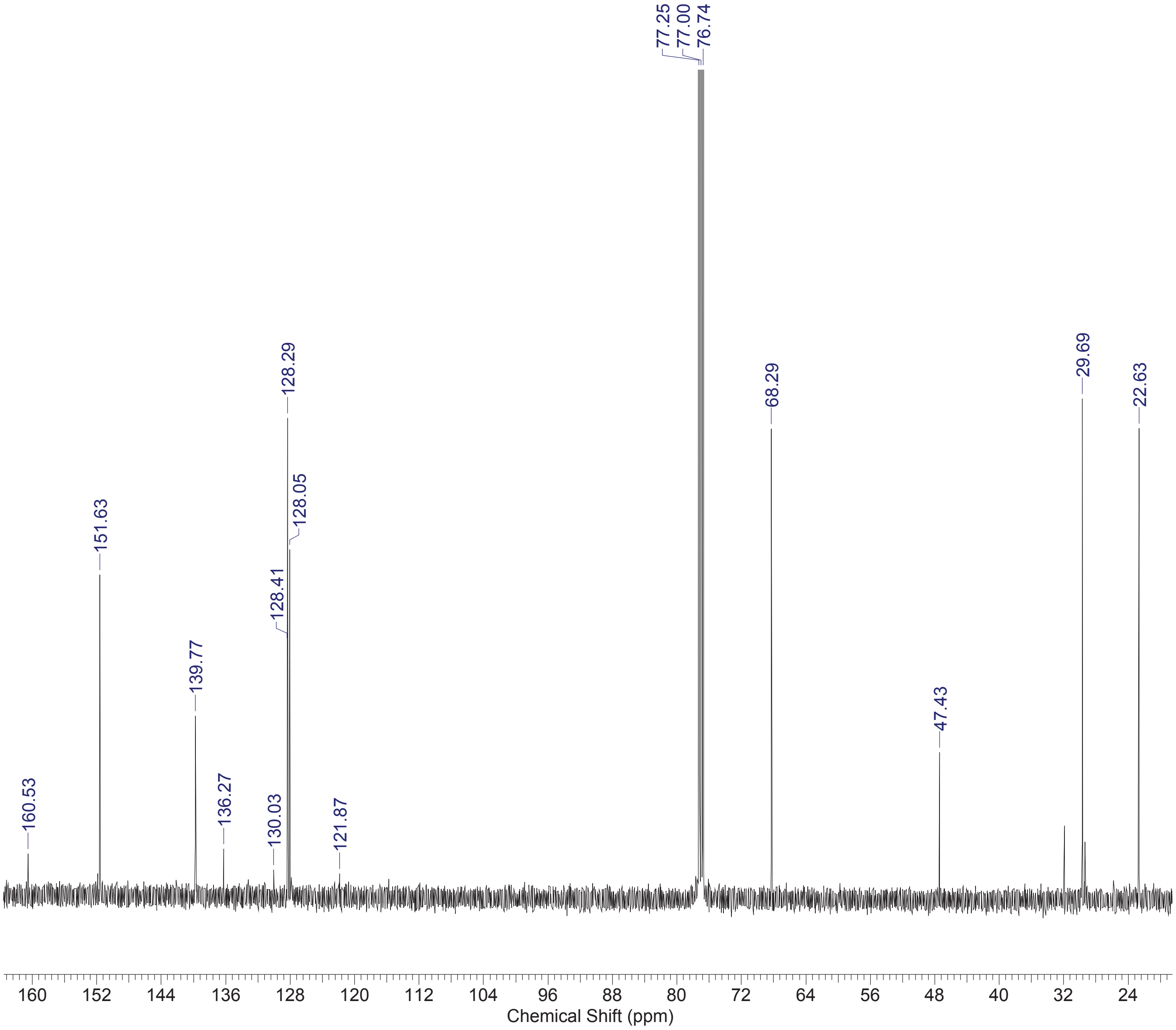

No.	(ppm)	(Hz)	Height
1	22.63	2844.3	0.2148
2	29.69	3731.2	0.2284
3	47.43	5961.0	0.0663
4	68.29	8582.7	0.2146
5	76.74	9645.1	1.0000
6	77.00	9677.5	0.9472
7	77.25	9709.0	0.9887
8	121.87	15316.8	0.0107
9	128.05	16094.1	0.1592
10	128.29	16124.6	0.2194
11	128.41	16138.9	0.1165
12	130.03	16343.0	0.0125
13	136.27	17126.9	0.0220
14	139.77	17567.5	0.0830
15	151.63	19057.2	0.1476
16	160.53	20175.9	0.0197

A5. 6-(Benzyloxy)-9-isopropyl-9H-purine (H-NMR)

12 Jul 2012

Acquisition Time (sec)	1.9923	***Date***	Feb 20 2012		
File Name	C:\Users\usuario\Documents\Espectros Asier\RMN-145-12_ASIMJ-4\proton				
Frequency (MHz)	499.79	***Nucleus***	1H	***Number of Transients***	8
Original Points Count	15964	***Points Count***	16384	***Pulse Sequence***	s2pul
Solvent	CHLOROFORM-D			***Sweep Width (Hz)***	8012.82
Temperature (degree C)	25.000				

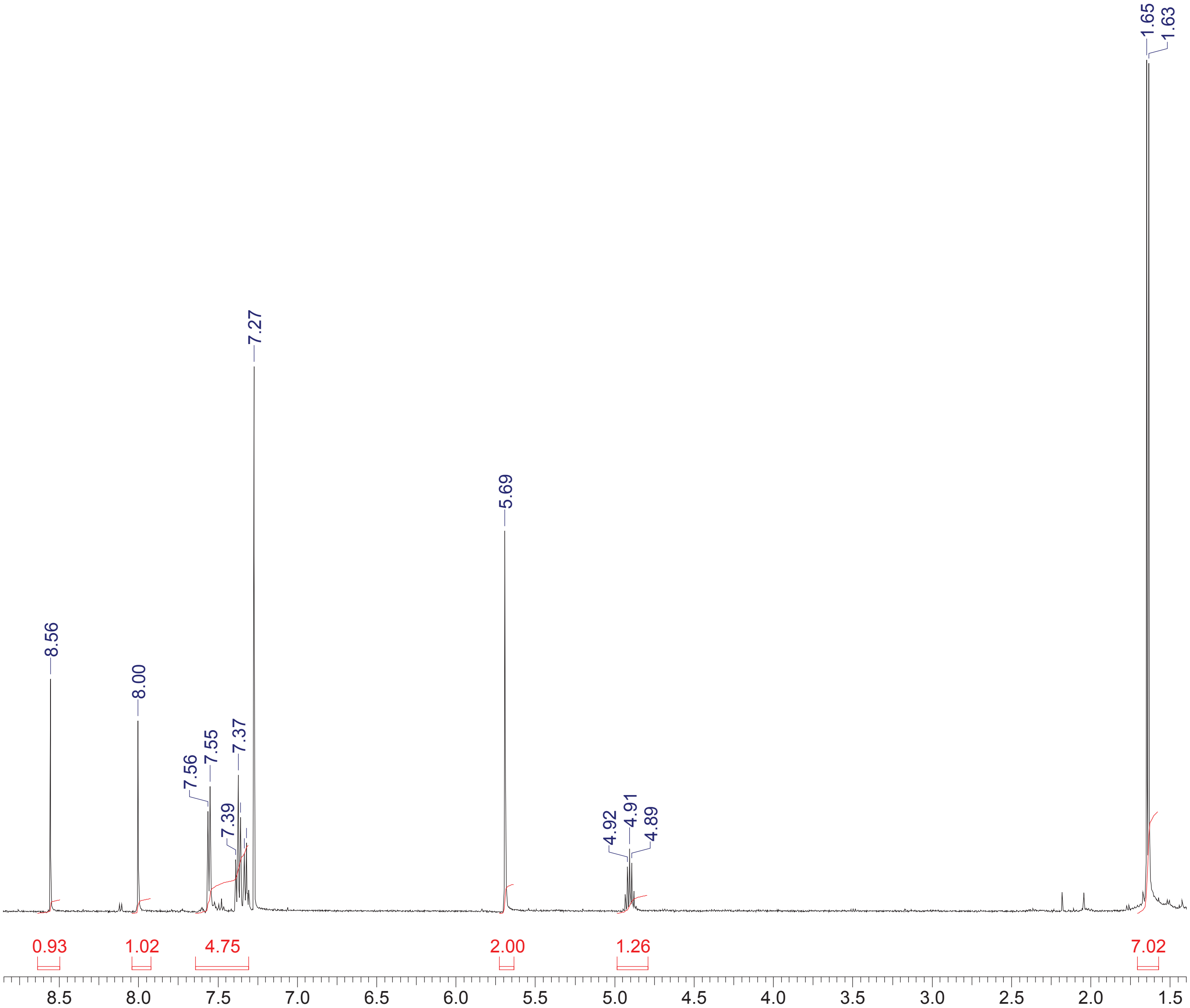

No.	(ppm)	(Hz)	Height
1	1.63	816.9	0.9483
2	1.65	823.8	0.9517
3	4.89	2445.1	0.0564
4	4.91	2452.0	0.0723
5	4.92	2458.8	0.0524
6	5.69	2845.2	0.4268
7	7.27	3634.6	0.6098
8	7.32	3658.6	0.0785
9	7.33	3665.9	0.0672
10	7.36	3677.6	0.1072
11	7.37	3685.0	0.1544
12	7.39	3692.3	0.0603
13	7.55	3773.0	0.1416
14	7.56	3780.4	0.1137
15	8.00	4000.0	0.2149
16	8.56	4276.3	0.2617

No.	(ppm)	Value	Absolute Value
1	[1.57 .. 1.71]	7.019	9.08332e+6
2	[4.79 .. 4.98]	1.259	1.62902e+6
3	[5.64 .. 5.73]	2.000	2.58826e+6
4	[7.31 .. 7.64]	4.745	6.14069e+6
5	[7.92 .. 8.04]	1.024	1.32578e+6
6	[8.50 .. 8.64]	0.932	1.20635e+6

A5. 6-(Benzyloxy)-9-isopropyl-9H-purine (MS)

Elemental Composition Report

Single Mass Analysis

Tolerance = 5.0 PPM / DBE: min = -1.5, max = 50.0
Element prediction: Off
Number of isotope peaks used for i-FIT = 3

Monoisotopic Mass, Even Electron Ions
498 formula(e) evaluated with 4 results within limits (up to 50 best isotopic matches for each mass)
Elements Used:
C: 0-22 H: 0-1000 N: 0-5 O: 0-6 F: 0-3

12/3292

ASIMJ-4 9 (0.217)

1: TOF MS ES+
4.60e+001

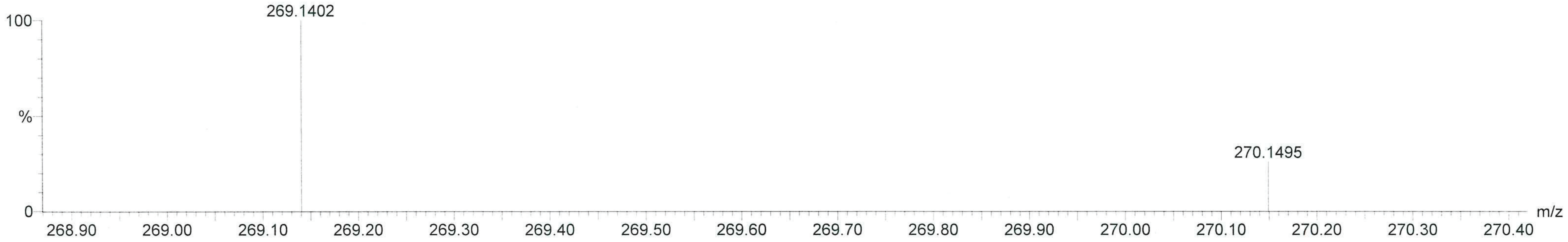

Minimum:				-1.5		
Maximum:		20.0	5.0	50.0		

Mass	Calc. Mass	mDa	PPM	DBE	i-FIT	Formula
269.1402	269.1402	0.0	0.0	9.5	n/a	C15 H17 N4 O
	269.1414	-1.2	-4.5	5.5	n/a	C12 H18 N4 O2 F
	269.1389	1.3	4.8	4.5	n/a	C14 H21 O5
	269.1400	0.2	0.7	0.5	n/a	C11 H22 O6 F

B1, 10. 6-Benzyloxi-9-isopropyl-8-phenyl-9H-purine (H-NMR)

9 Jul 2012

Acquisition Time (sec)	2.0487	***Comment***	ASIMJ-6p	***Date***	Apr 19 2012
File Name	C:\Users\usuario\Documents\Espectros Asier\12-5265_ASIMJ-6p\1H-ASIMJ-6p				
Frequency (MHz)	300.20	***Nucleus***	1H	***Number of Transients***	64
Original Points Count	7380	***Points Count***	8192	***Pulse Sequence***	s2pul
Solvent	CHLOROFORM-D			***Sweep Width (Hz)***	3602.31
Temperature (degree C)	30.000				

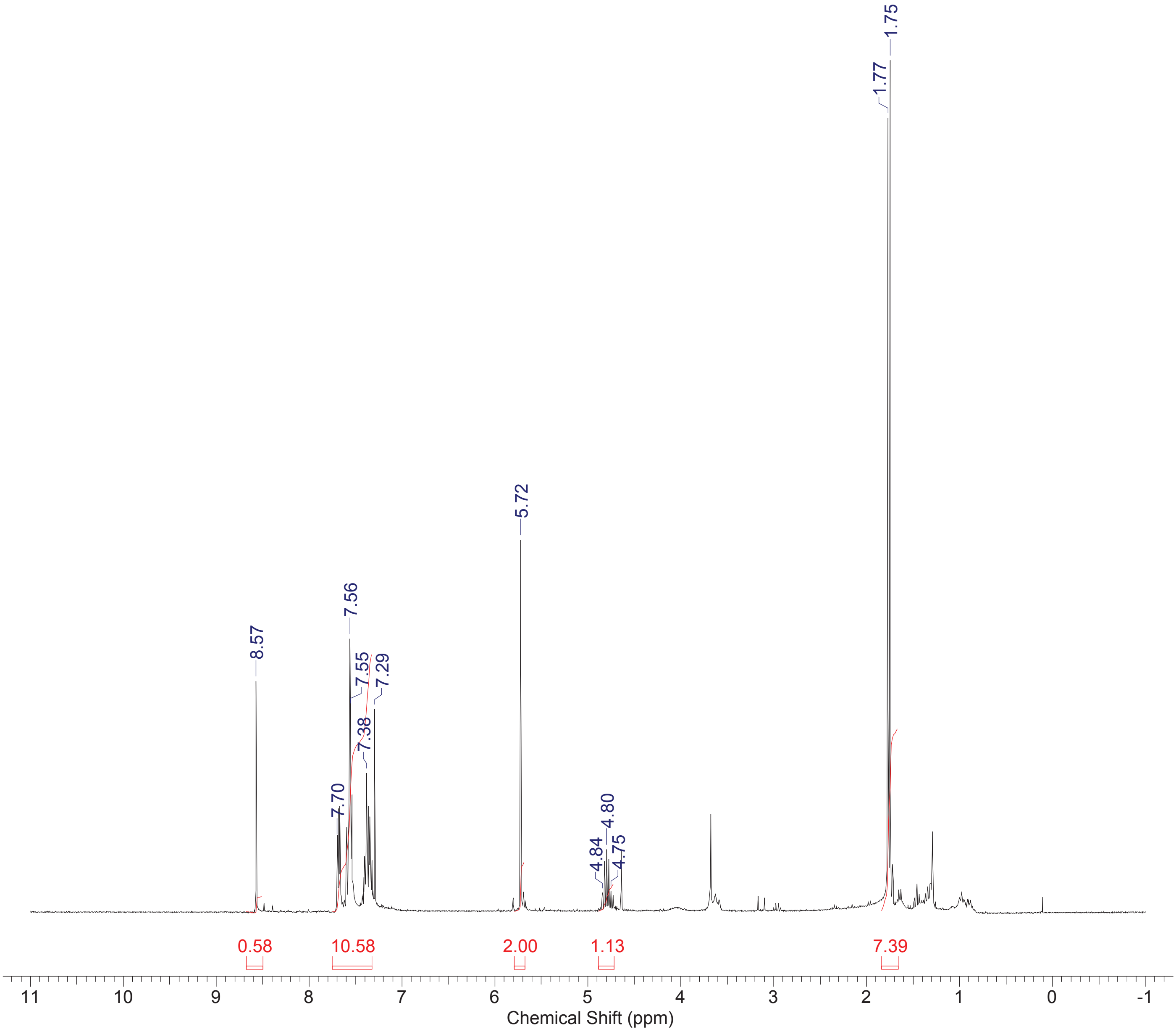

No.	(ppm)	(Hz)	Height
1	1.75	524.4	1.0000
2	1.77	531.5	0.9324
3	4.75	1426.0	0.0237
4	4.80	1439.6	0.0729
5	4.84	1453.7	0.0214
6	5.72	1717.6	0.4364
7	7.29	2189.5	0.2378
8	7.38	2215.9	0.1621
9	7.55	2267.8	0.2390
10	7.56	2270.4	0.3199
11	7.70	2311.7	0.0844
12	8.57	2572.5	0.2705

No.	(ppm)	Value	Absolute Value
1	[1.66 .. 1.84]	7.393	2.72495e+8
2	[4.72 .. 4.88]	1.126	4.14961e+7
3	[5.68 .. 5.79]	2.000	7.37148e+7
4	[7.32 .. 7.75]	10.583	3.90054e+8
5	[8.50 .. 8.68]	0.582	2.14378e+7

B1, 10. 6-Benzyloxy-9-isopropyl-8-phenyl-9H-purine (MS)

Elemental Composition Report

Single Mass Analysis
Tolerance = 5.0 PPM / DBE: min = -1.5, max = 50.0
Element prediction: Off
Number of isotope peaks used for i-FIT = 3

Monoisotopic Mass, Even Electron Ions
136 formula(e) evaluated with 1 results within limits (up to 50 best isotopic matches for each mass)
Elements Used:
C: 0-21 H: 0-1000 N: 0-4 O: 0-4 Na: 0-1

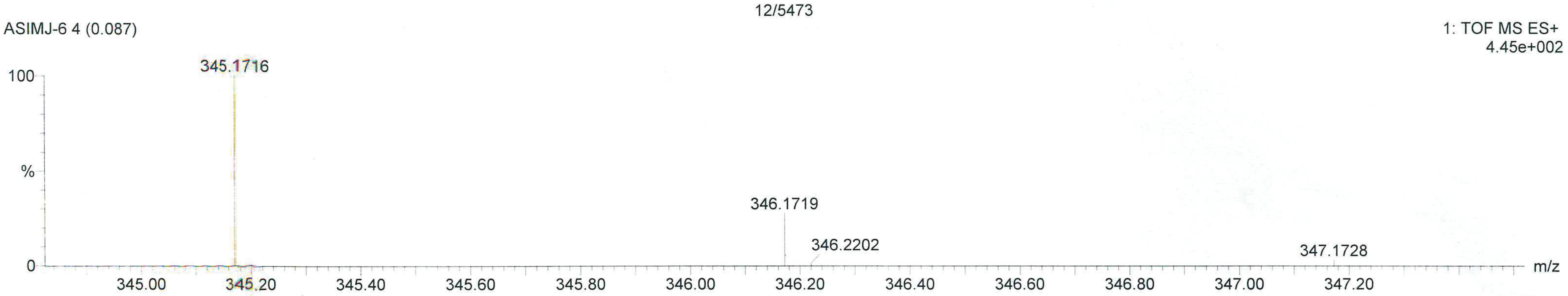

Minimum:				-1.5		
Maximum:		20.0	5.0	50.0		
Mass	Calc. Mass	mDa	PPM	DBE	i-FIT	Formula
345.1716	345.1715	0.1	0.3	13.5	0.5	C21 H21 N4 O

B1, 10. 6-Benzyloxy-9-isopropyl-8-phenyl-9H-purine (C-NMR)

9 Jul 2012

Acquisition Time (sec)	1.3005	***Comment***	ASIMJ-6p	***Date***	Apr 19 2012
File Name	C:\Users\usuario\Documents\Espectros Asier\12-5265_ASIMJ-6p\13C-ASIMJ-6p				
Frequency (MHz)	75.49	***Nucleus***	13C	***Number of Transients***	6400
Original Points Count	23559	***Points Count***	32768	***Pulse Sequence***	s2pul
Solvent	CHLOROFORM-D			***Sweep Width (Hz)***	18115.94
Temperature (degree C)	30.000				

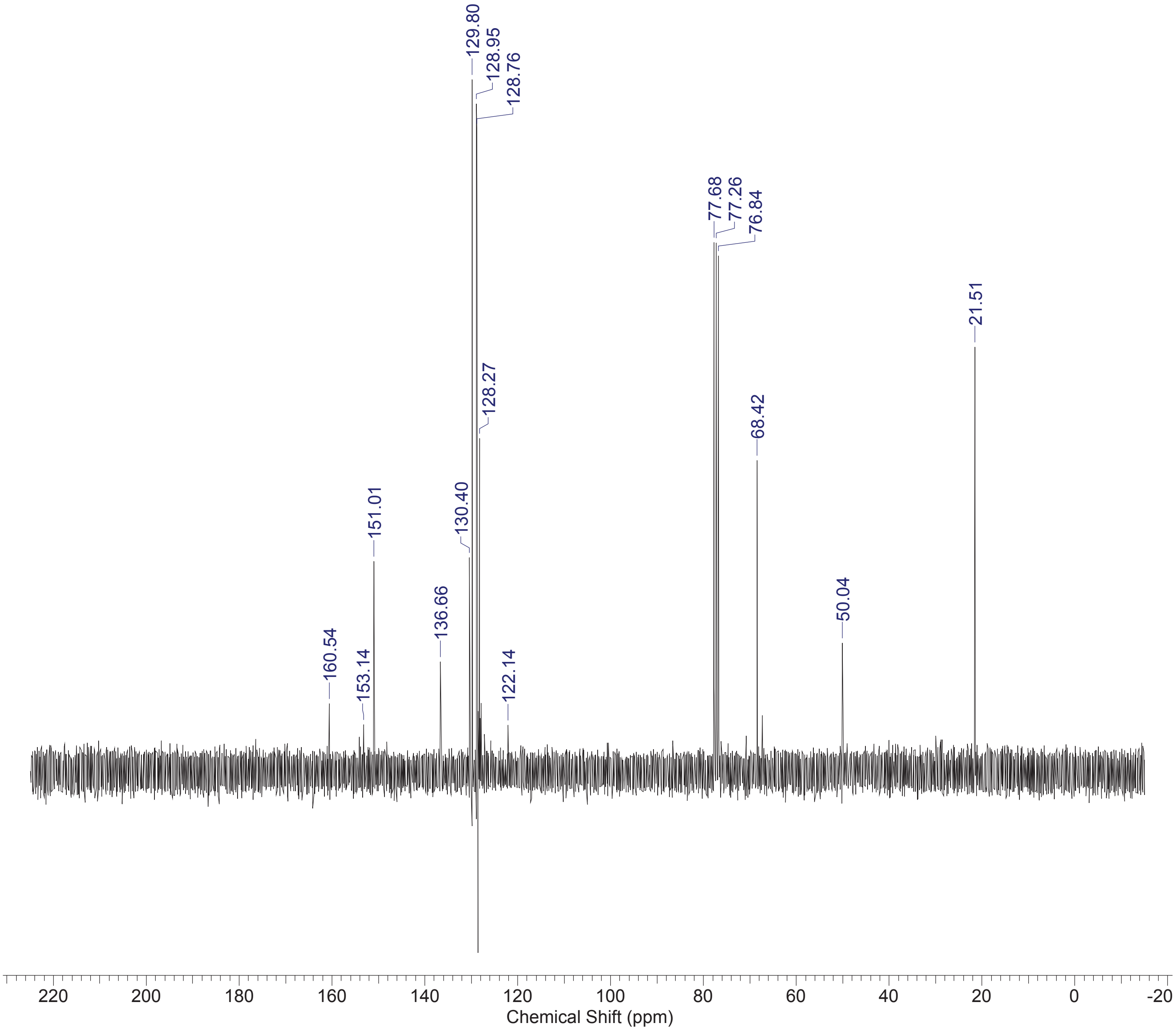

No.	(ppm)	(Hz)	Height
1	21.51	1624.0	0.6139
2	50.04	3777.4	0.1866
3	68.42	5165.1	0.4500
4	76.84	5800.9	0.7455
5	77.26	5832.4	0.7640
6	77.68	5864.5	0.7647
7	122.14	9220.4	0.0674
8	128.27	9683.8	0.4819
9	128.61	9709.2	0.8357
10	128.76	9720.2	0.9293
11	128.95	9734.6	0.9649
12	129.80	9799.3	1.0000
13	130.40	9844.1	0.3097
14	136.66	10316.8	0.1588
15	151.01	11399.9	0.3044
16	153.14	11560.8	0.0682
17	160.54	12119.7	0.0983

B1, 10. 6-Benzyloxy-9-isopropyl-8-phenyl-9H-purine (DEPT)

9 Jul 2012

**Acquisition Time (sec)**	1.0000	**Comment**	ASIMJ-6p	**Date**	Apr 19 2012
**File Name**	C:\Users\usuario\Documents\Espectros Asier\12-5265_ASIMJ-6p\DEPT-ASIMJ-6p				
**Frequency (MHz)**	75.49	**Nucleus**	13C	**Number of Transients**	4800
**Original Points Count**	18116	**Points Count**	32768	**Pulse Sequence**	DEPT135
**Solvent**	CHLOROFORM-D			**Sweep Width (Hz)**	18115.94
**Temperature (degree C)**	30.000				

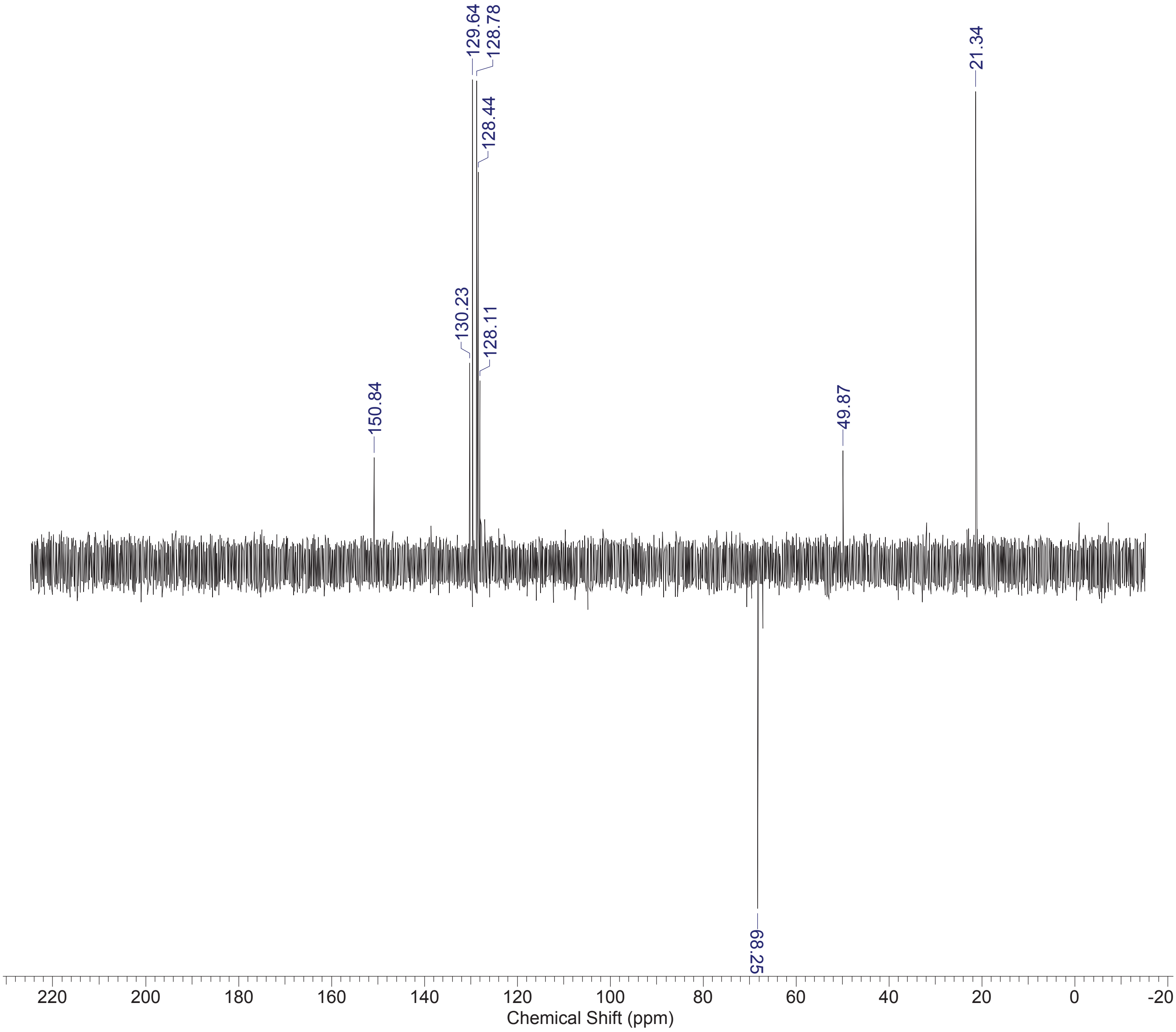

No.	(ppm)	(Hz)	Height
1	21.34	1611.3	0.9748
2	49.87	3764.7	0.2347
3	68.25	5152.5	-0.7110
4	128.11	9671.1	0.3783
5	128.44	9696.5	0.8089
6	128.59	9707.6	0.8026
7	128.78	9721.9	0.9972
8	129.64	9786.6	1.0000
9	130.23	9831.4	0.4147
10	150.84	11387.2	0.2201

B2 9-tert-butyl-6-(benzyloxy)-8-phenyl-9H-purine (MS)

Elemental Composition Report

Single Mass Analysis

Tolerance = 5.0 PPM / DBE: min = -1.5, max = 50.0
Element prediction: Off
Number of isotope peaks used for i-FIT = 3

Monoisotopic Mass, Even Electron Ions
635 formula(e) evaluated with 1 results within limits (up to 50 closest results for each mass)
Elements Used:
C: 0-22 H: 0-1000 N: 0-5 O: 0-20 Na: 0-1

12/21702

ASIMJ-27P3 14 (0.317)

1: TOF MS ES+
8.90e+002

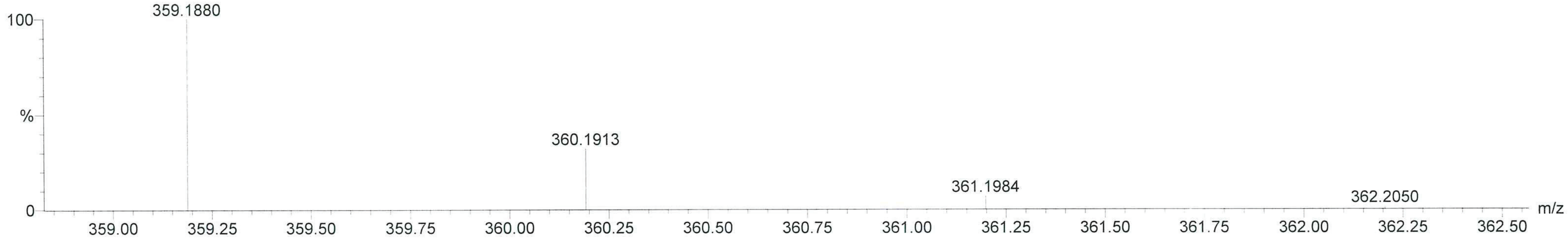

Minimum:				-1.5		
Maximum:		5.0	5.0	50.0		
Mass	Calc. Mass	mDa	PPM	DBE	i-FIT	Formula
359.1880	359.1872	0.8	2.2	13.5	10.3	C22 H23 N4 O

B2. 9-tert-butyl-6-(benzyloxy)-8-phenyl-9H-purine (C-NMR)

12 Nov 2012

Acquisition Time (sec)	1.3005	***Comment***	ASIMJ-27-P2-P	***Date***	Oct 22 2012
File Name	C:\Users\usuario\Documents\Espectros Asier\12-21939_ASIMJ-27-P2-P\13C-ASIMJ-27-P2-P				
Frequency (MHz)	75.49	***Nucleus***	13C	***Number of Transients***	10000
Original Points Count	23559	***Points Count***	32768	***Pulse Sequence***	s2pul
Solvent	CHLOROFORM-D			***Sweep Width (Hz)***	18115.94
Temperature (degree C)	30.000				

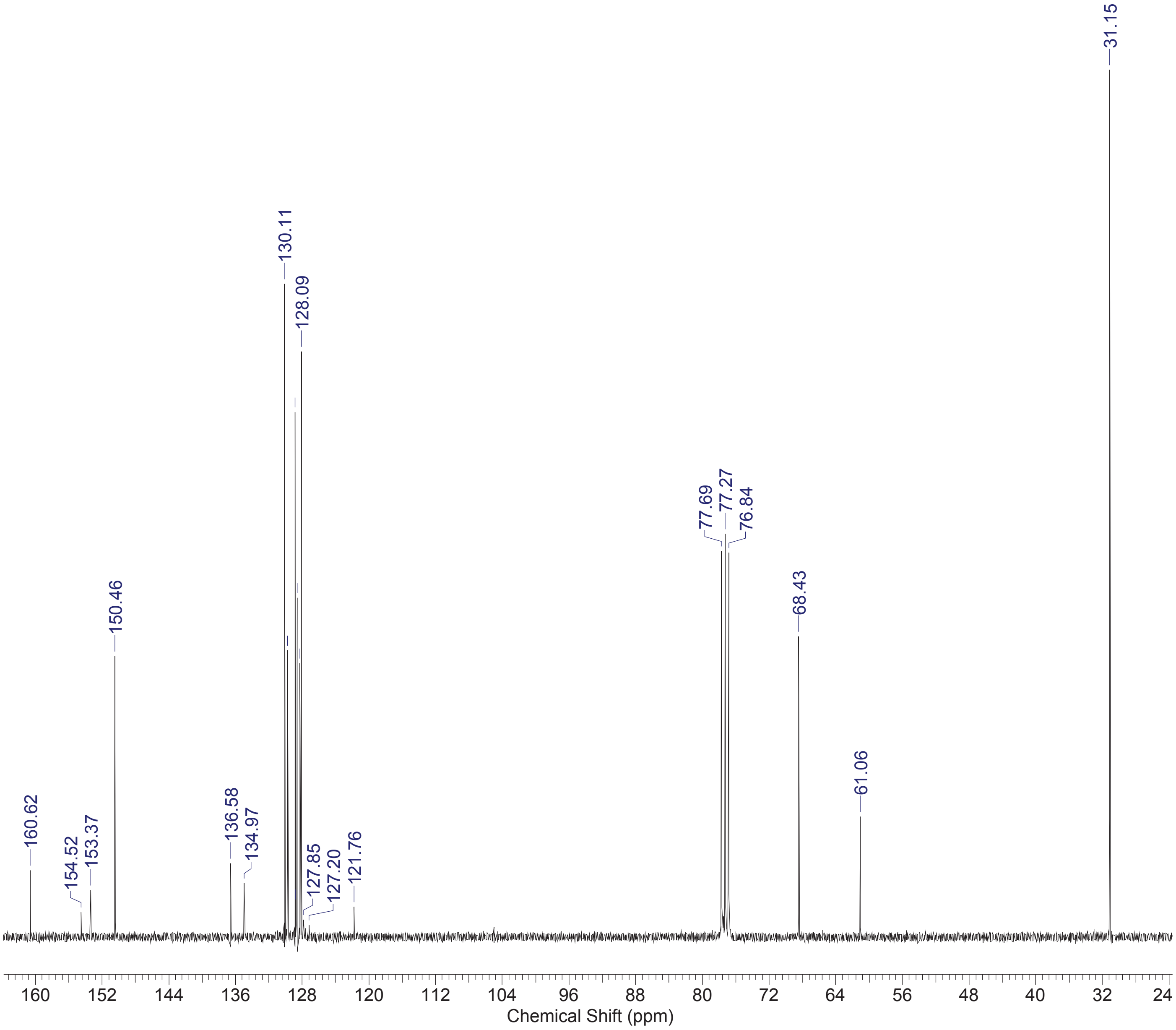

No.	(ppm)	(Hz)	Height	No.	(ppm)	(Hz)	Height
1	31.15	2351.6	1.0000	13	128.59	9707.5	0.3914
2	61.06	4609.5	0.1388	14	128.67	9713.6	0.0190
3	68.43	5166.3	0.3470	15	128.78	9721.9	0.0381
4	76.84	5800.9	0.4431	16	128.85	9727.4	0.6056
5	77.27	5833.0	0.4649	17	129.72	9793.2	0.3308
6	77.69	5865.1	0.4451	18	130.11	9822.5	0.7535
7	121.76	9192.3	0.0353	19	134.97	10189.6	0.0624
8	127.20	9602.5	0.0139	20	136.58	10310.7	0.0852
9	127.85	9651.7	0.0204	21	150.46	11358.4	0.3239
10	128.09	9669.9	0.6750	22	153.37	11578.5	0.0544
11	128.27	9683.8	0.3159	23	154.52	11665.3	0.0286
12	128.52	9702.6	0.0149	24	160.62	12125.8	0.0769

B2. 9-tert-butyl-6-(benzyloxy)-8-phenyl-9H-purine (H-NMR)

12 Nov 2012

Acquisition Time (sec)	2.0487	***Comment***	ASIMJ-27-P2-P	***Date***	Oct 19 2012
File Name	C:\Users\usuario\Documents\Espectros Asier\12-21939_ASIMJ-27-P2-P\1H-ASIMJ-27-P2-P				
Frequency (MHz)	300.20	***Nucleus***	1H	***Number of Transients***	64
Original Points Count	7380	***Points Count***	8192	***Pulse Sequence***	s2pul
Solvent	CHLOROFORM-D			***Sweep Width (Hz)***	3602.31
Temperature (degree C)	30.000				

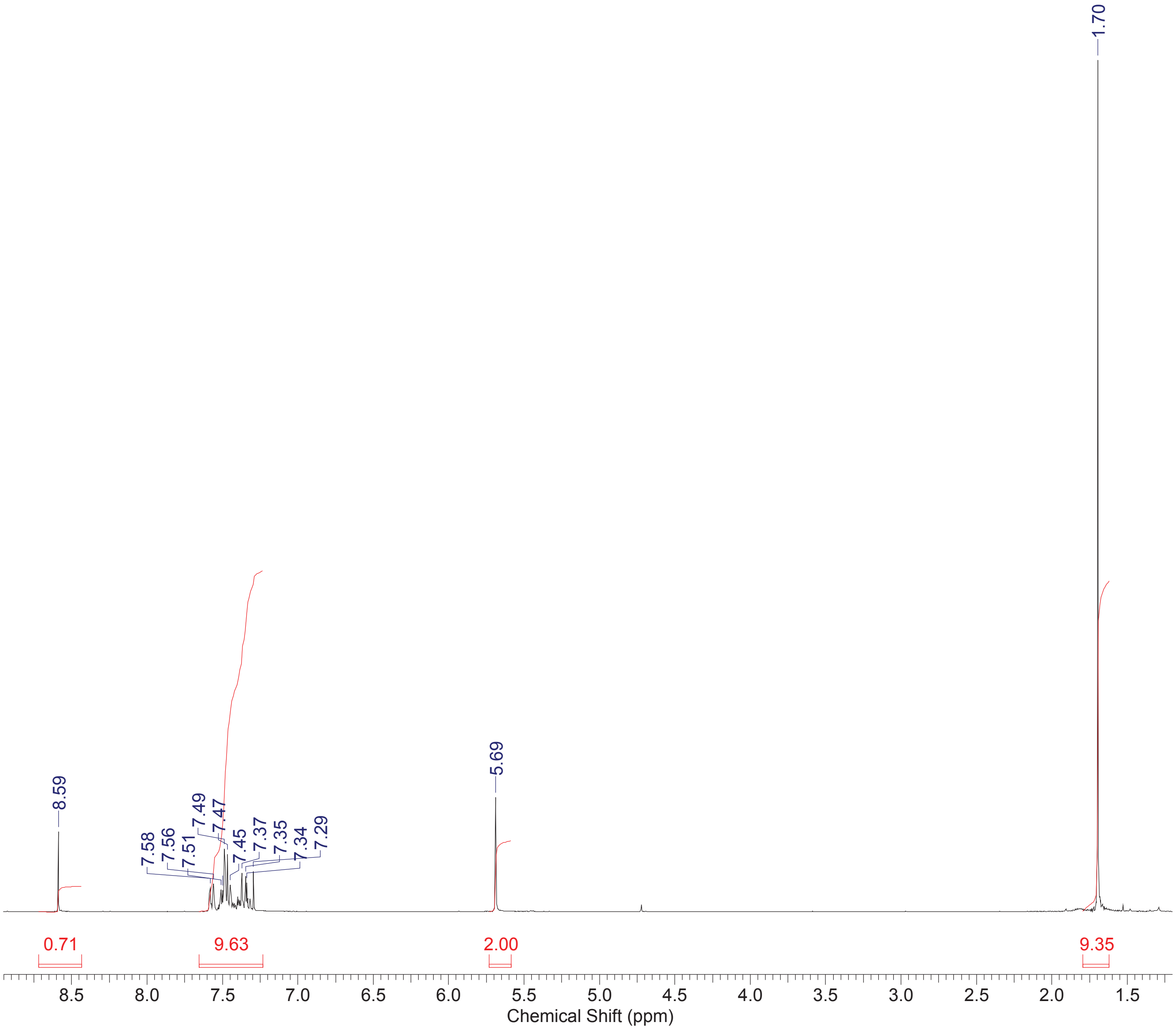

No.	(ppm)	(Hz)	Height
1	1.70	509.0	1.0000
2	5.69	1707.9	0.1344
3	7.29	2189.9	0.0470
4	7.34	2203.1	0.0338
5	7.35	2205.3	0.0415
6	7.37	2212.8	0.0455
7	7.45	2236.1	0.0317
8	7.47	2241.4	0.0675
9	7.49	2247.5	0.0736
10	7.50	2251.0	0.0253
11	7.51	2254.6	0.0254
12	7.56	2269.5	0.0329
13	7.58	2276.1	0.0273
14	8.59	2578.2	0.0940

No.	(ppm)	Value	Absolute Value
1	[1.62 .. 1.80]	9.348	7.90278e+8
2	[5.58 .. 5.73]	2.000	1.69088e+8
3	[7.23 .. 7.66]	9.629	8.14099e+8
4	[8.43 .. 8.72]	0.706	5.96692e+7

B3. 6-(3-methoxybenzyloxy)-9-isopropyl-8-(3-methoxyphenyl)-9H-purine (C-NMR)

2 Jul 2013

Acquisition Time (sec)	1.0486	***Comment***	new experiment	***Date***	Jun 3 2013
File Name	C:\Users\usuario\Documents\Espectros 2"Gen-Asier\13-13401_2G-ASIMJ-4\carbono				
Frequency (MHz)	125.68	***Nucleus***	13C	***Number of Transients***	1200
Original Points Count	32768	***Points Count***	32768	***Pulse Sequence***	s2pul
Solvent	CHLOROFORM-D			***Sweep Width (Hz)***	31250.00
Temperature (degree C)	25.000				

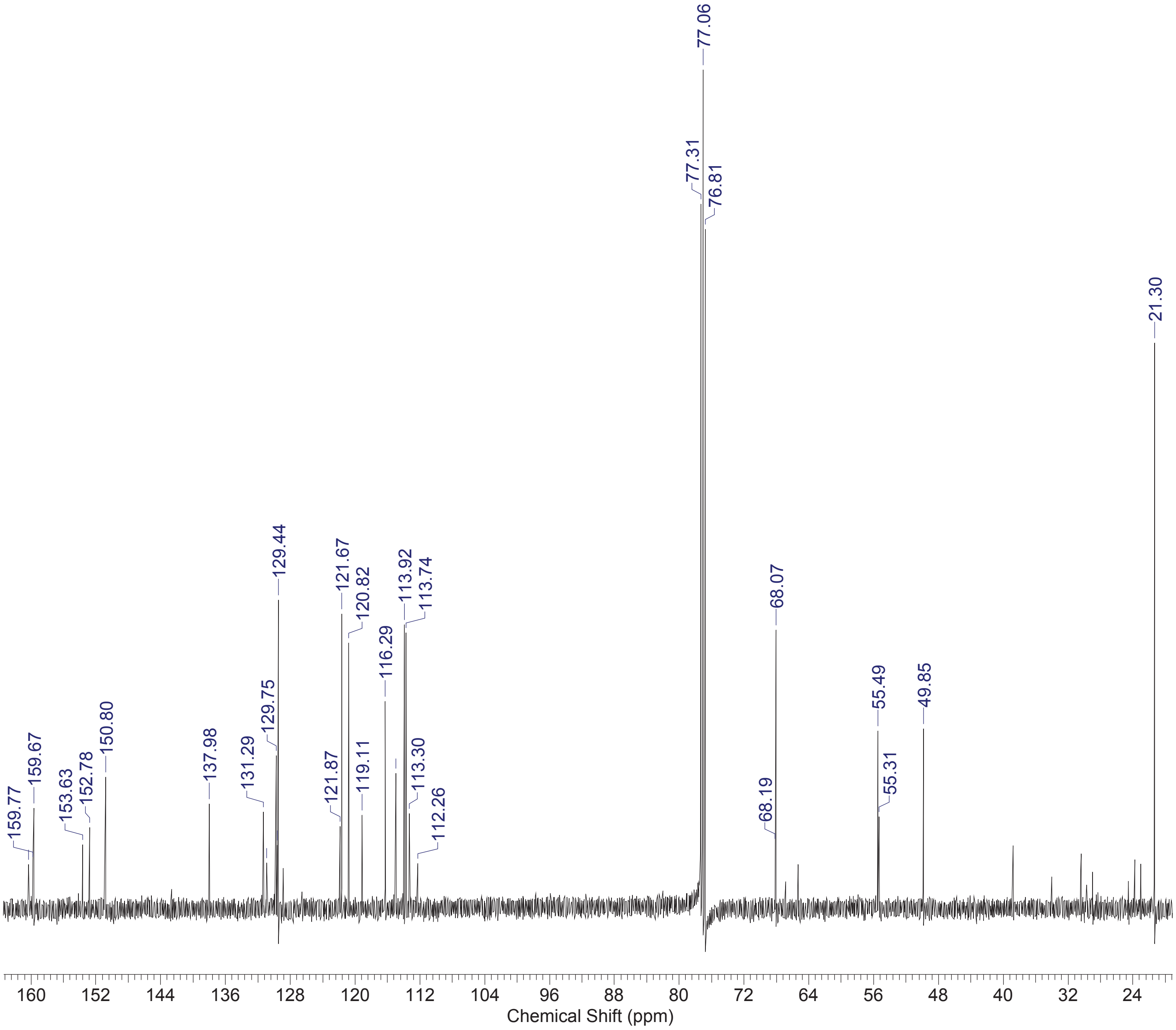

No.	(ppm)	(Hz)	Height	No.	(ppm)	(Hz)	Height
1	21.30	2676.9	0.6743	17	119.11	14970.1	0.1106
2	49.85	6265.7	0.2134	18	120.82	15184.7	0.3164
3	55.26	6945.7	0.0986	19	121.67	15291.5	0.3509
4	55.31	6951.4	0.1089	20	121.87	15317.3	0.0970
5	55.49	6974.3	0.2111	21	129.44	16269.1	0.3675
6	68.07	8555.5	0.3317	22	129.63	16292.0	0.0748
7	68.19	8570.8	0.0769	23	129.75	16308.2	0.1815
8	76.81	9653.3	0.8097	24	130.91	16453.2	0.0534
9	77.06	9684.7	1.0000	25	131.29	16500.8	0.1145
10	77.31	9717.1	0.8400	26	137.98	17342.0	0.1240
11	112.26	14109.9	0.0529	27	150.80	18953.8	0.1563
12	113.30	14240.6	0.1126	28	152.78	19202.7	0.0955
13	113.74	14294.9	0.3282	29	153.63	19308.5	0.0752
14	113.92	14317.8	0.3379	30	159.67	20068.6	0.1188
15	114.96	14448.5	0.1602	31	159.77	20081.0	0.0549
16	116.29	14616.3	0.2461	32	160.27	20144.0	0.0514

B3. 6-(3-methoxybenzyloxy)-9-isopropyl-8-(3-methoxyphenyl)-9H-purine (DEPT)

6 Jun 2013

**Acquisition Time (sec)**	1.0486	**Comment**	new experiment	**Date**	Jun 3 2013
**File Name**	C:\Users\usuario\Documents\Espectros 2"Gen-Asier\13-13401_2G-ASIMJ-4\dept				
**Frequency (MHz)**	125.68	**Nucleus**	13C	**Number of Transients**	1200
**Original Points Count**	32768	**Points Count**	32768	**Pulse Sequence**	DEPT135
**Solvent**	CHLOROFORM-D			**Sweep Width (Hz)**	31250.00
**Temperature (degree C)**	25.000				

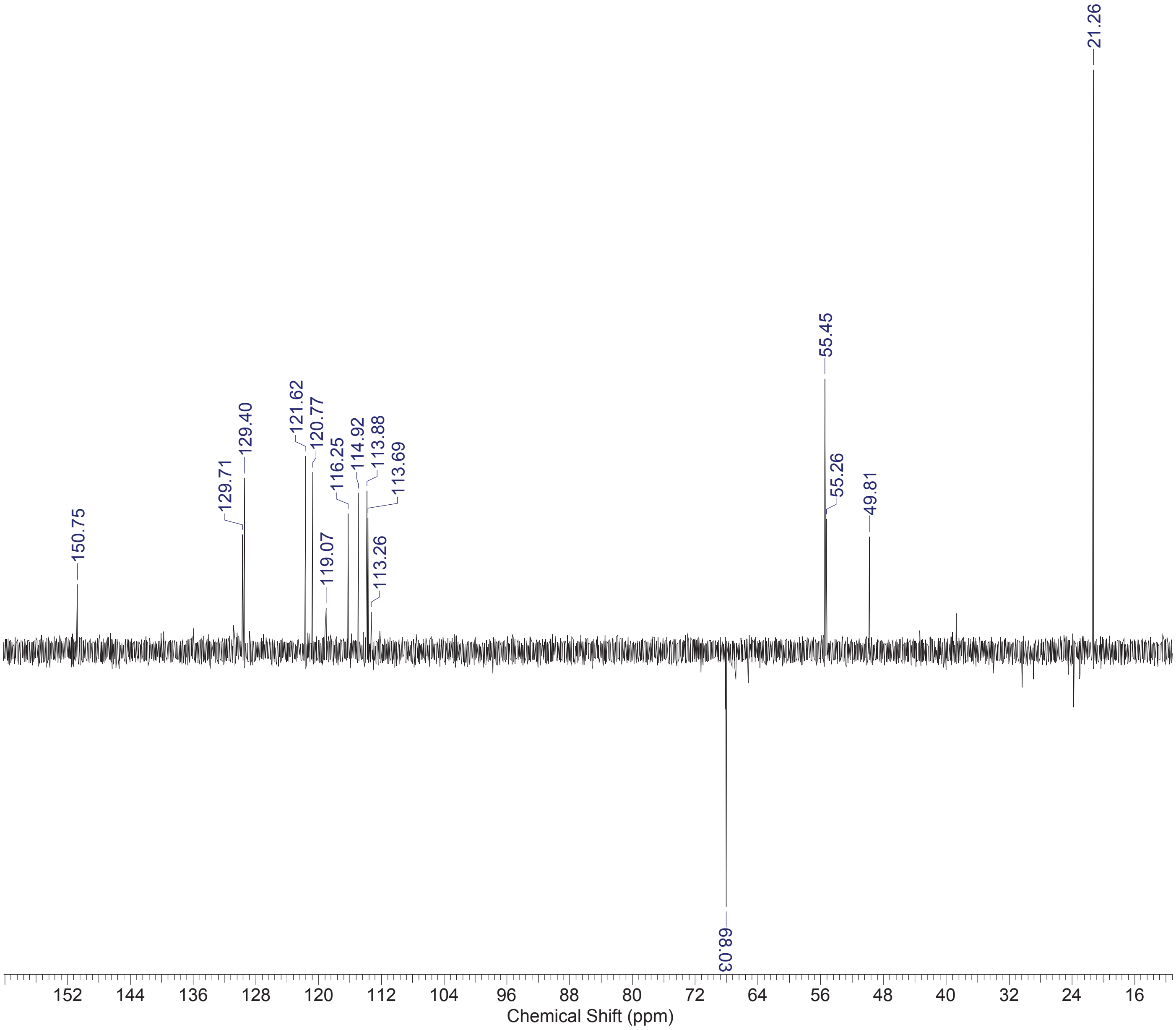

No.	(ppm)	(Hz)	Height
1	21.26	2671.6	1.0000
2	49.81	6260.4	0.1963
3	55.22	6940.4	0.1545
4	55.26	6945.2	0.2267
5	55.45	6969.0	0.4680
6	68.03	8550.3	-0.4404
7	113.26	14235.3	0.0677
8	113.69	14289.7	0.2291
9	113.88	14312.5	0.2754
10	114.92	14443.2	0.2714
11	116.25	14611.1	0.2360
12	119.07	14964.9	0.0744
13	120.77	15179.5	0.3076
14	121.62	15286.3	0.3350
15	129.40	16263.8	0.2975
16	129.71	16302.9	0.1999
17	150.75	18947.5	0.1144

B3. 6-(3-methoxybenzyloxy)-9-isopropyl-8-(3-methoxyphenyl)-9H-purine (H-NMR)

2 Jul 2013

**Acquisition Time (sec)**	2.0447	**Date**	Jun 3 2013		
**File Name**	C:\Users\usuario\Documents\Espectros 2"Gen-Asier\13-13401_2G-ASIMJ-4\proton				
**Frequency (MHz)**	499.79	**Nucleus**	1H	**Number of Transients**	8
**Original Points Count**	16384	**Points Count**	16384	**Pulse Sequence**	s2pul
**Solvent**	CHLOROFORM-D			**Sweep Width (Hz)**	8012.82
**Temperature (degree C)**	25.000				

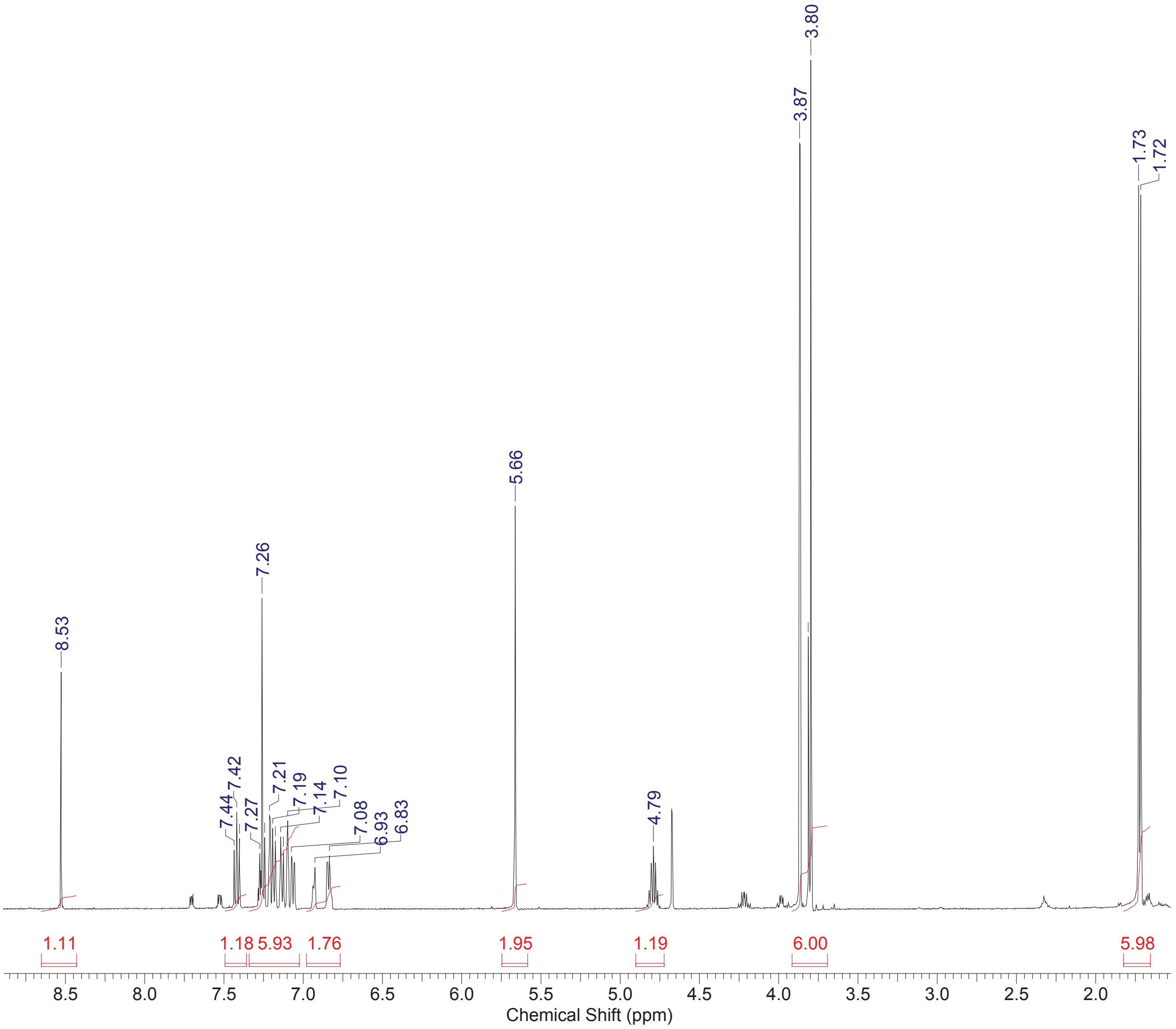

No.	(ppm)	(Hz)	Height
1	1.72	858.2	0.8415
2	1.73	865.1	0.8527
3	3.80	1898.0	1.0000
4	3.81	1905.9	0.3225
5	3.87	1933.3	0.9023
6	4.79	2394.0	0.0764
7	5.66	2830.7	0.4761
8	6.83	3415.7	0.0656
9	6.93	3461.2	0.0517
10	7.08	3536.0	0.0642
11	7.10	3547.3	0.1063
12	7.13	3561.5	0.0704
13	7.14	3568.8	0.0876
14	7.18	3586.4	0.0823
15	7.19	3594.2	0.0979
16	7.21	3604.5	0.1128
17	7.24	3620.1	0.0868
18	7.26	3628.5	0.3677
19	7.27	3635.8	0.0678
20	7.40	3699.9	0.0857
21	7.42	3707.7	0.1164
22	7.44	3716.0	0.0716
23	8.53	4262.3	0.2806

No.	(ppm)	Value	Absolute Value
1	[1.66 .. 1.82]	5.979	6.38854e+7
2	[3.69 .. 3.92]	6.000	6.41093e+7
3	[4.72 .. 4.90]	1.187	1.26874e+7
4	[5.58 .. 5.75]	1.947	2.08065e+7
5	[6.77 .. 6.98]	1.756	1.87648e+7
6	[7.02 .. 7.34]	5.934	6.34028e+7
7	[7.36 .. 7.49]	1.177	1.25738e+7
8	[8.43 .. 8.65]	1.106	1.18136e+7

B3. 6-(3-methoxybenzyloxy)-9-isopropyl-8-(3-methoxyphenyl)-9H-purine (MS)

Elemental Composition Report **Page 1**

Single Mass Analysis
Tolerance = 10.0 PPM / DBE: min = -1.5, max = 50.0
Element prediction: Off
Number of isotope peaks used for i-FIT = 3

Monoisotopic Mass, Even Electron Ions
359 formula(e) evaluated with 2 results within limits (up to 50 best isotopic matches for each mass)
Elements Used:
C: 0-23 H: 0-1000 N: 0-4 O: 0-4 Na: 0-1 I: 0-2

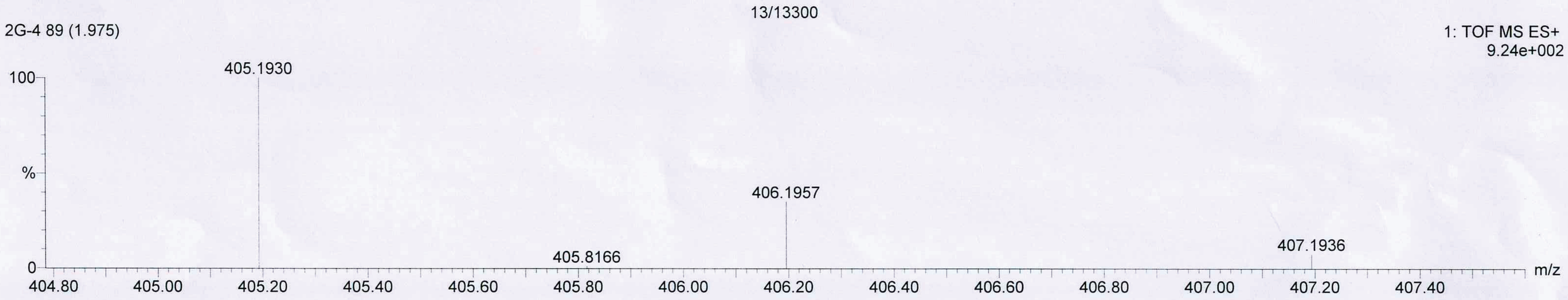

Minimum:				-1.5		
Maximum:		10.0	10.0	50.0		
Mass	Calc. Mass	mDa	PPM	DBE	i-FIT	Formula
405.1930	405.1927	0.3	0.7	13.5	12.2	C23 H25 N4 O3
	405.1903	2.7	6.7	10.5	19.1	C21 H26 N4 O3 Na

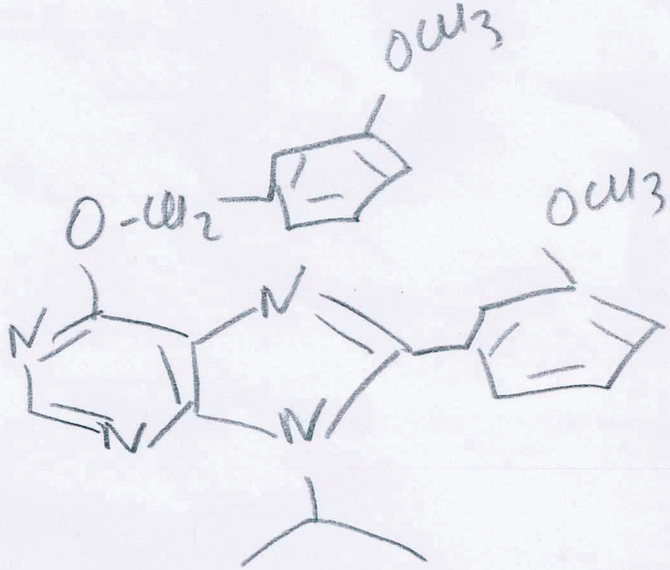

B4 6-(3-methoxybenzyloxy)-9-tert-butyl-8-(3-methoxyphenyl)-9H-purine (C-NMR)

2 Jul 2013

**Acquisition Time (sec)**	1.0486	**Date**	May 9 2013
**File Name**	C:\Users\usuario\Documents\Espectros 2”Gen-Asier\13-11181_2ASIMJ-1_500\CARBON_01		
**Frequency (MHz)**	125.68	**Nucleus**	13C
**Number of Transients**	9600		
**Original Points Count**	32768	**Points Count**	32768
**Pulse Sequence**	s2pul		
**Solvent**	CHLOROFORM-D	**Sweep Width (Hz)**	31250.00
**Temperature (degree C)**	25.000		

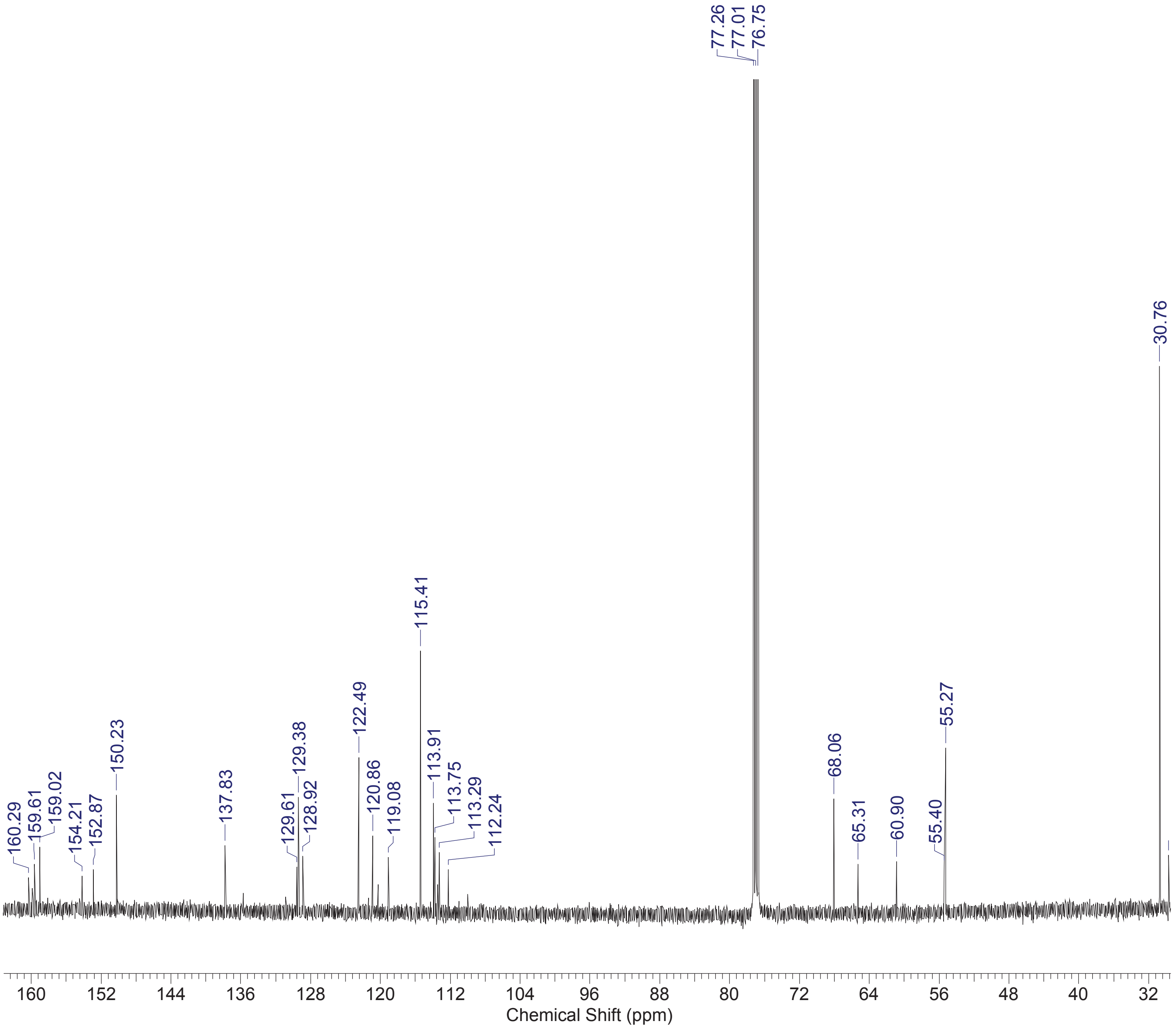

No.	(ppm)	(Hz)	Height
1	29.69	3732.2	0.0320
2	30.76	3865.7	0.3134
3	55.27	6946.1	0.0937
4	55.40	6963.3	0.0260
5	60.90	7653.8	0.0282
6	65.31	8207.9	0.0267
7	68.06	8554.1	0.0643
8	76.75	9646.1	0.9387
9	77.01	9678.5	1.0000
10	77.26	9710.0	0.9149
11	112.24	14106.5	0.0238
12	113.29	14239.1	0.0335
13	113.75	14296.3	0.0422
14	113.91	14317.3	0.0619

No.	(ppm)	(Hz)	Height
15	115.41	14505.2	0.1495
16	119.08	14966.8	0.0306
17	120.86	15190.0	0.0431
18	122.49	15395.0	0.0882
19	128.92	16203.7	0.0316
20	129.38	16261.0	0.0655
21	129.61	16289.6	0.0254
22	137.83	17323.4	0.0375
23	150.23	18881.7	0.0665
24	152.87	19213.6	0.0236
25	154.21	19381.5	0.0199
26	159.02	19986.1	0.0368
27	159.61	20060.5	0.0268
28	160.29	20146.3	0.0191

B4. 6-(3-methoxybenzyloxy)-9-tert-butyl-8-(3-methoxyphenyl)-9H-purine (DEPT)

2 Jul 2013

Acquisition Time (sec)	1.0486	***Date***	May 9 2013		
File Name	C:\Users\usuario\Documents\Espectros 2"Gen-Asier\13-11181_2ASIMJ-1_500\DEPT_01				
Frequency (MHz)	125.68	***Nucleus***	13C	***Number of Transients***	4800
Original Points Count	32768	***Points Count***	32768	***Pulse Sequence***	DEPT135
Solvent	CHLOROFORM-D			***Sweep Width (Hz)***	31250.00
Temperature (degree C)	25.000				

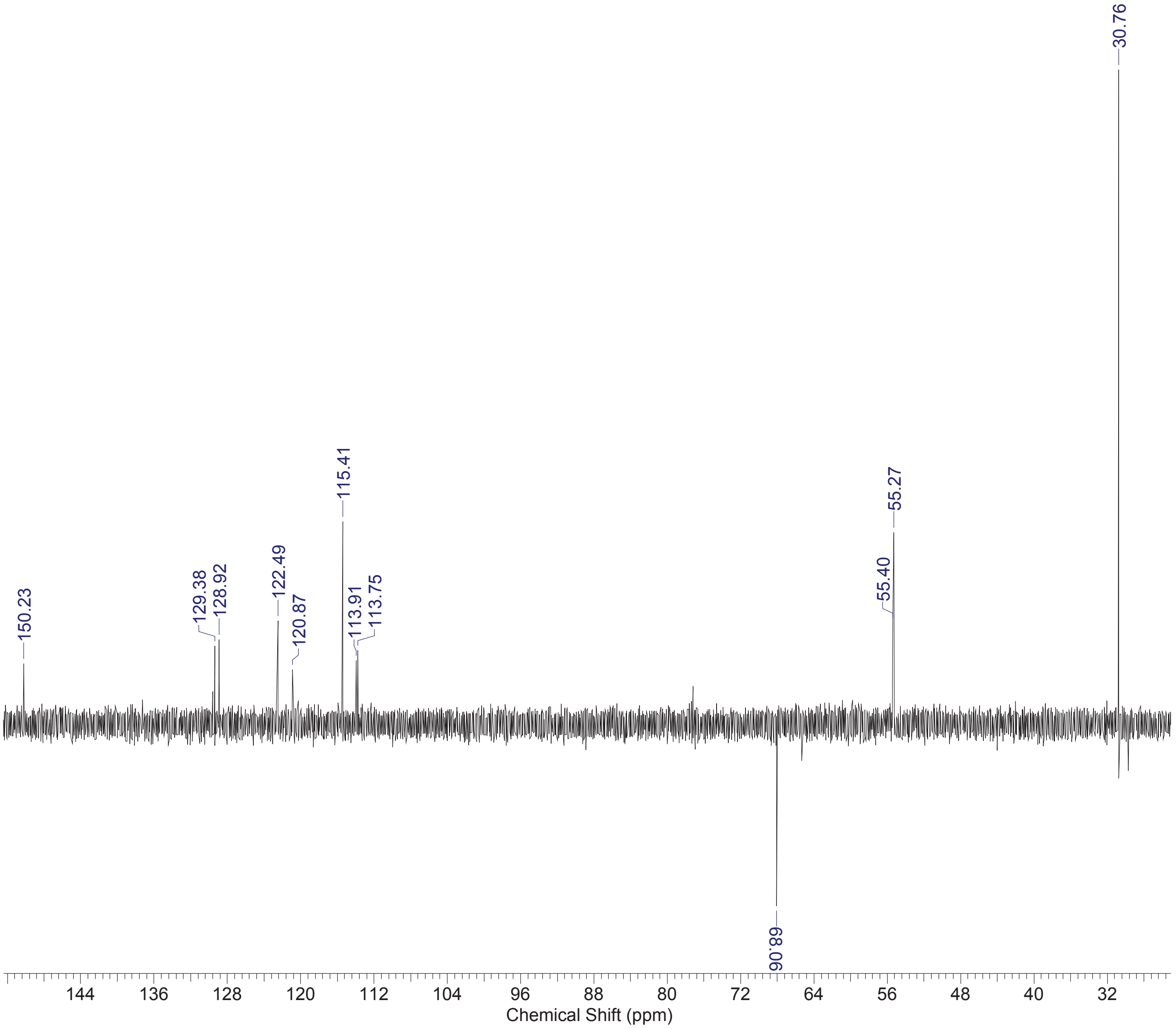

No.	(ppm)	(Hz)	Height
1	30.76	3865.7	1.0000
2	55.27	6946.1	0.2925
3	55.40	6963.3	0.1543
4	68.06	8554.1	-0.2783
5	113.75	14296.3	0.1128
6	113.91	14317.3	0.0972
7	115.41	14505.2	0.3092
8	120.87	15190.9	0.0830
9	122.49	15395.0	0.1573
10	128.92	16203.7	0.1286
11	129.38	16261.0	0.1188
12	150.23	18881.7	0.0920

B4. 6-(3-methoxybenzyloxy)-9-tert-butyl-8-(3-methoxyphenyl)-9H-purine (H-NMR)

7 May 2013

Acquisition Time (sec)	2.5559	***Date***	May 7 2013		
File Name	C:\Users\usuario\Documents\Espectros 2”Gen-Asier\13-11181_2ASIMJ-1\proton				
Frequency (MHz)	400.57	***Nucleus***	1H	***Number of Transients***	8
Original Points Count	16384	***Points Count***	16384	***Pulse Sequence***	s2pul
Solvent	CHLOROFORM-D			***Sweep Width (Hz)***	6410.26
Temperature (degree C)	30.000				

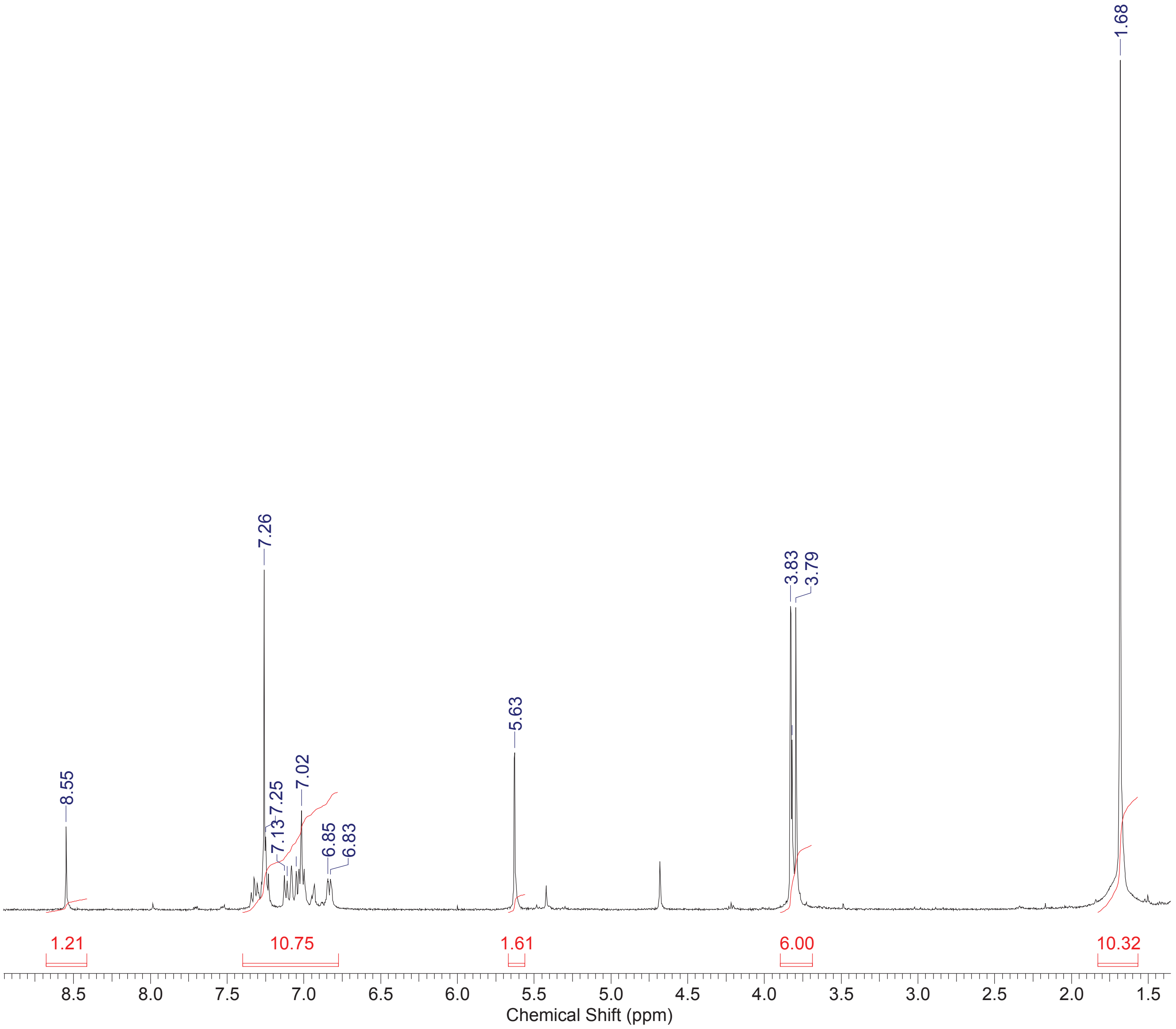

No.	(ppm)	(Hz)	Height
1	0.07	27.3	0.0985
2	1.68	674.1	1.0000
3	3.79	1519.7	0.3582
4	3.82	1529.8	0.2027
5	3.83	1533.7	0.3594
6	5.63	2254.1	0.1875
7	6.83	2734.2	0.0392
8	6.85	2742.0	0.0397
9	7.02	2810.1	0.1198
10	7.05	2824.2	0.0487
11	7.11	2846.9	0.0375
12	7.13	2854.7	0.0438
13	7.25	2903.6	0.0888
14	7.26	2907.9	0.4022
15	8.55	3424.4	0.1006

No.	(ppm)	Value	Absolute Value
1	[1.57 .. 1.83]	10.325	4.28525e+8
2	[3.69 .. 3.90]	6.000	2.49022e+8
3	[5.56 .. 5.67]	1.614	6.70018e+7
4	[6.77 .. 7.40]	10.753	4.46282e+8
5	[8.41 .. 8.68]	1.215	5.04239e+7

B4. 6-(3-methoxybenzyloxy)-9-tert-butyl-8-(3-methoxyphenyl)-9H-purine (MS)

Elemental Composition Report

Single Mass Analysis

Tolerance = 5.0 mDa / DBE: min = -1.5, max = 50.0
Element prediction: Off
Number of isotope peaks used for i-FIT = 3

Monoisotopic Mass, Even Electron Ions
468 formula(e) evaluated with 6 results within limits (up to 50 best isotopic matches for each mass)
Elements Used:
C: 0-24 H: 0-1000 N: 0-8 O: 0-7 Na: 0-1

MJN 40 (0.880)

1: TOF MS ES+

1.03e+002

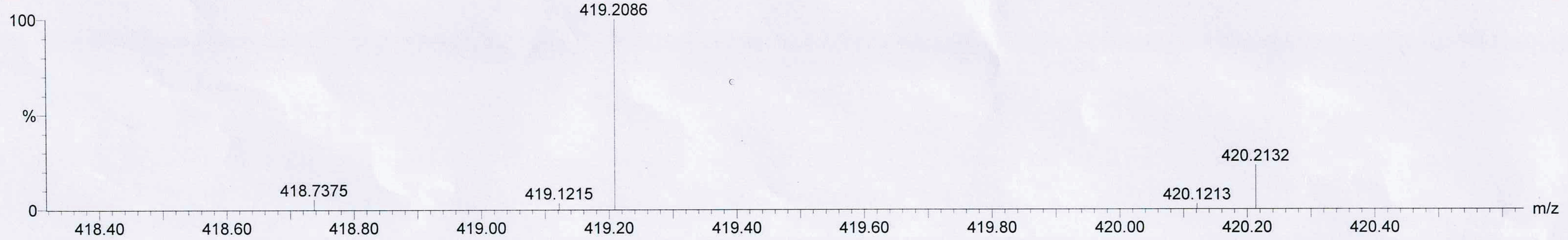

Minimum:				-1.5		
Maximum:		5.0	10.0	50.0		

Mass	Calc. Mass	mDa	PPM	DBE	i-FIT	Formula
419.2086	419.2083	0.3	0.7	13.5	n/a	C24 H27 N4 O3
	419.2059	2.7	6.4	10.5	n/a	C22 H28 N4 O3 Na
	419.2043	4.3	10.3	9.5	n/a	C19 H27 N6 O5
	419.2131	-4.5	-10.7	6.5	n/a	C16 H28 N8 O4 Na
	419.2070	1.6	3.8	8.5	n/a	C23 H31 O7
	419.2046	4.0	9.5	5.5	n/a	C21 H32 O7 Na

B5. 9-isopropyl-6-methoxy-9H-purine (H-NMR)

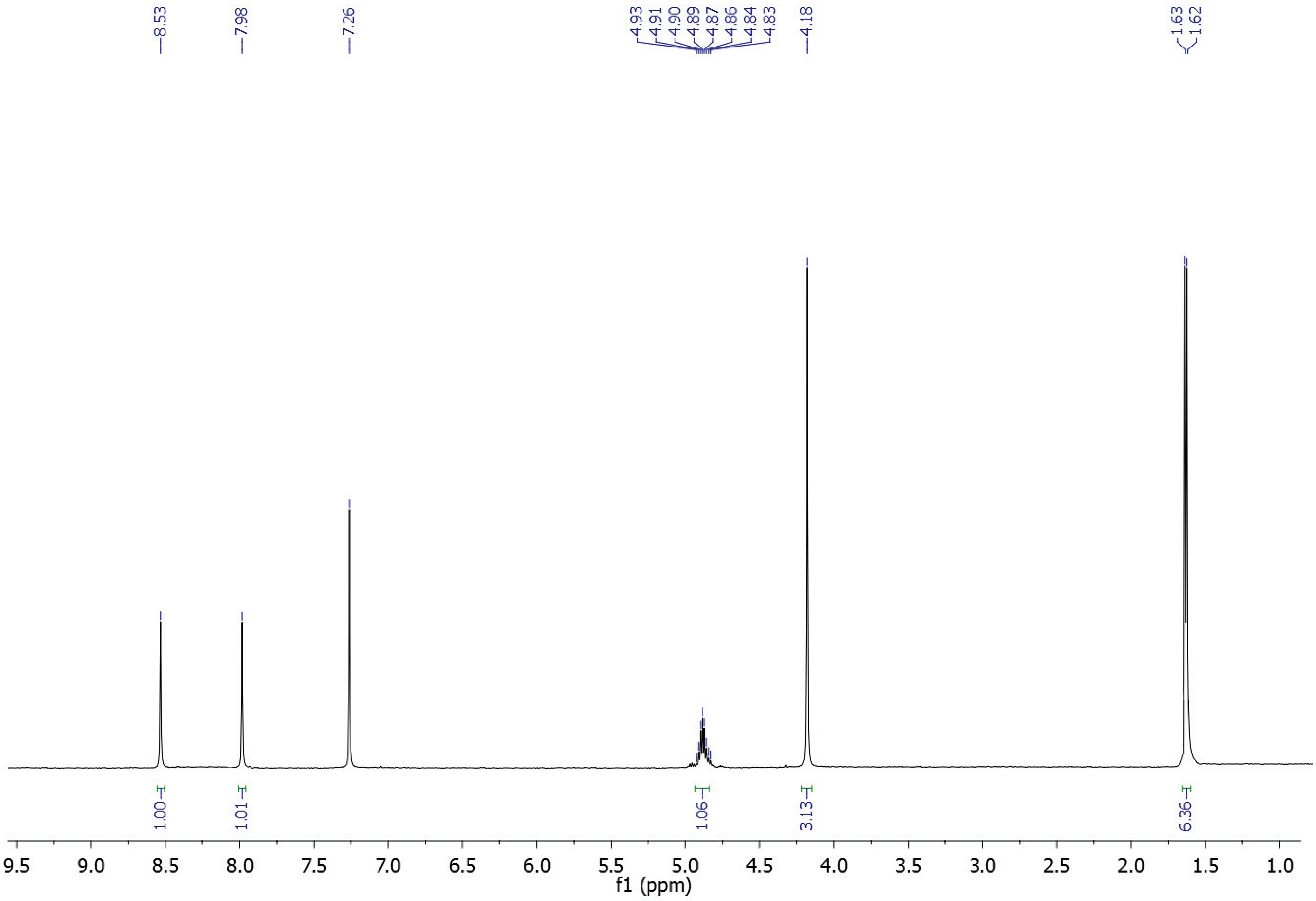

B5. 9-Isopropyl-6-methoxy-9H-purine (MS)

Elemental Composition Report

Single Mass Analysis

Tolerance = 5.0 PPM / DBE: min = -1.5, max = 50.0
Element prediction: Off
Number of isotope peaks used for i-FIT = 3

Monoisotopic Mass, Even Electron Ions
729 formula(e) evaluated with 1 results within limits (up to 50 closest results for each mass)
Elements Used:
C: 0-14 H: 0-1000 N: 0-5 O: 0-5 Na: 0-1 S: 0-1 Cl: 0-1 Br: 0-2

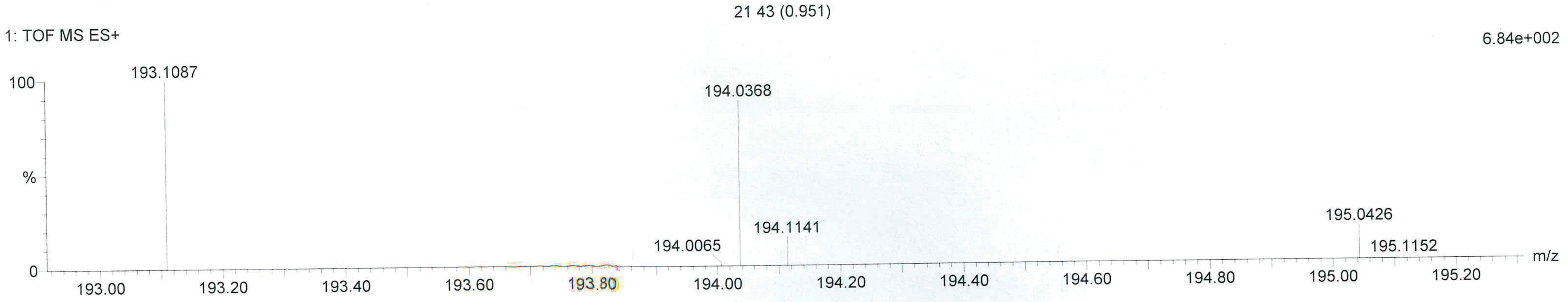

Minimum:				-1.5		
Maximum:		20.0	5.0	50.0		
Mass	Calc. Mass	mDa	PPM	DBE	i-FIT	Formula
193.1087	193.1089	-0.2	-1.0	5.5	2.7	C9 H13 N4 O

B5. 9-Isopropyl-6-methoxy-9H-purine(C-NMR)

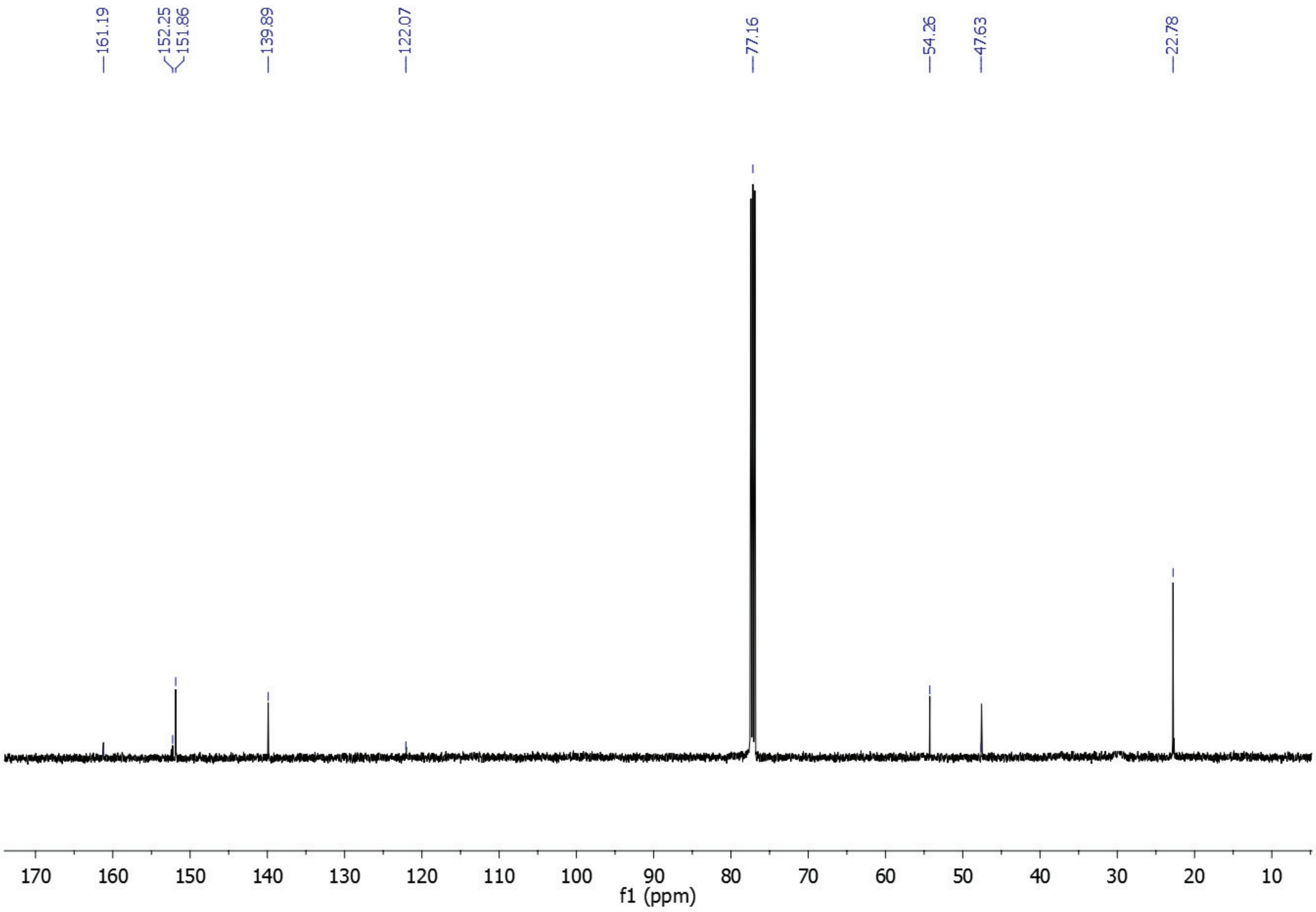

B6, B7 6-Ethoxy-9-isopropyl-8-methyl-9H-purine (H-NMR)

9 Jul 2012

Acquisition Time (sec)	2.0447	***Date***	Jun 8 2012		
File Name	C:\Users\usuario\Documents\Espectros Asier\12-11090_ASIMJ-3NP\PROTON_01				
Frequency (MHz)	499.79	***Nucleus***	1H	***Number of Transients***	8
Original Points Count	16384	***Points Count***	16384	***Pulse Sequence***	s2pul
Solvent	CHLOROFORM-D			***Sweep Width (Hz)***	8012.82
Temperature (degree C)	25.000				

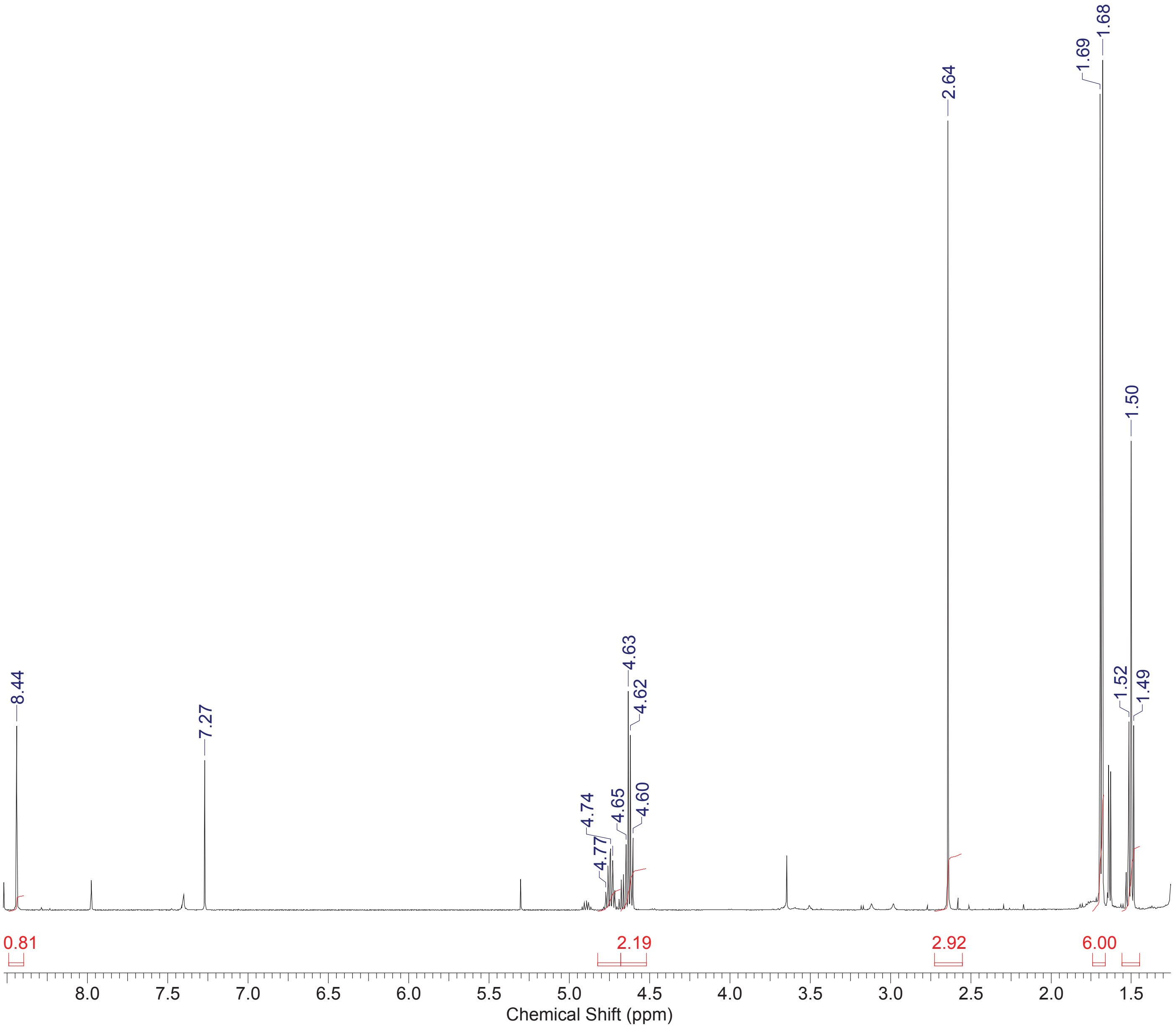

No.	(ppm)	(Hz)	Height
1	1.49	742.7	0.2185
2	1.50	750.1	0.5522
3	1.52	757.4	0.2229
4	1.68	839.6	1.0000
5	1.69	846.4	0.9601
6	2.64	1320.8	0.9286
7	4.60	2301.0	0.0863
8	4.62	2308.3	0.2071
9	4.63	2315.2	0.2585
10	4.65	2322.5	0.0785
11	4.73	2364.1	0.0599
12	4.74	2370.9	0.0736
13	4.77	2385.1	0.0228
14	7.27	3634.2	0.1774
15	8.44	4218.7	0.2174

No.	(ppm)	Value	Absolute Value
1	[1.45 .. 1.56]	3.311	1.98984e+8
2	[1.66 .. 1.74]	6.000	3.60639e+8
3	[2.55 .. 2.72]	2.923	1.75698e+8
4	[4.52 .. 4.68]	2.188	1.31488e+8
5	[4.68 .. 4.82]	1.122	6.74613e+7
6	[8.40 .. 8.49]	0.805	4.83939e+7

B6, B7. 6-Ethoxy-9-isopropyl-8-methyl-9H-purine (C-NMR)

9 Jul 2012

Acquisition Time (sec)	1.0486	***Date***	Jun 8 2012		
File Name	C:\Users\usuario\Documents\Espectros Asier\12-11090_ASIMJ-3NP\CARBON_01				
Frequency (MHz)	125.68	***Nucleus***	13C	***Number of Transients***	9600
Original Points Count	32768	***Points Count***	32768	***Pulse Sequence***	s2pul
Solvent	CHLOROFORM-D			***Sweep Width (Hz)***	31250.00
Temperature (degree C)	25.000				

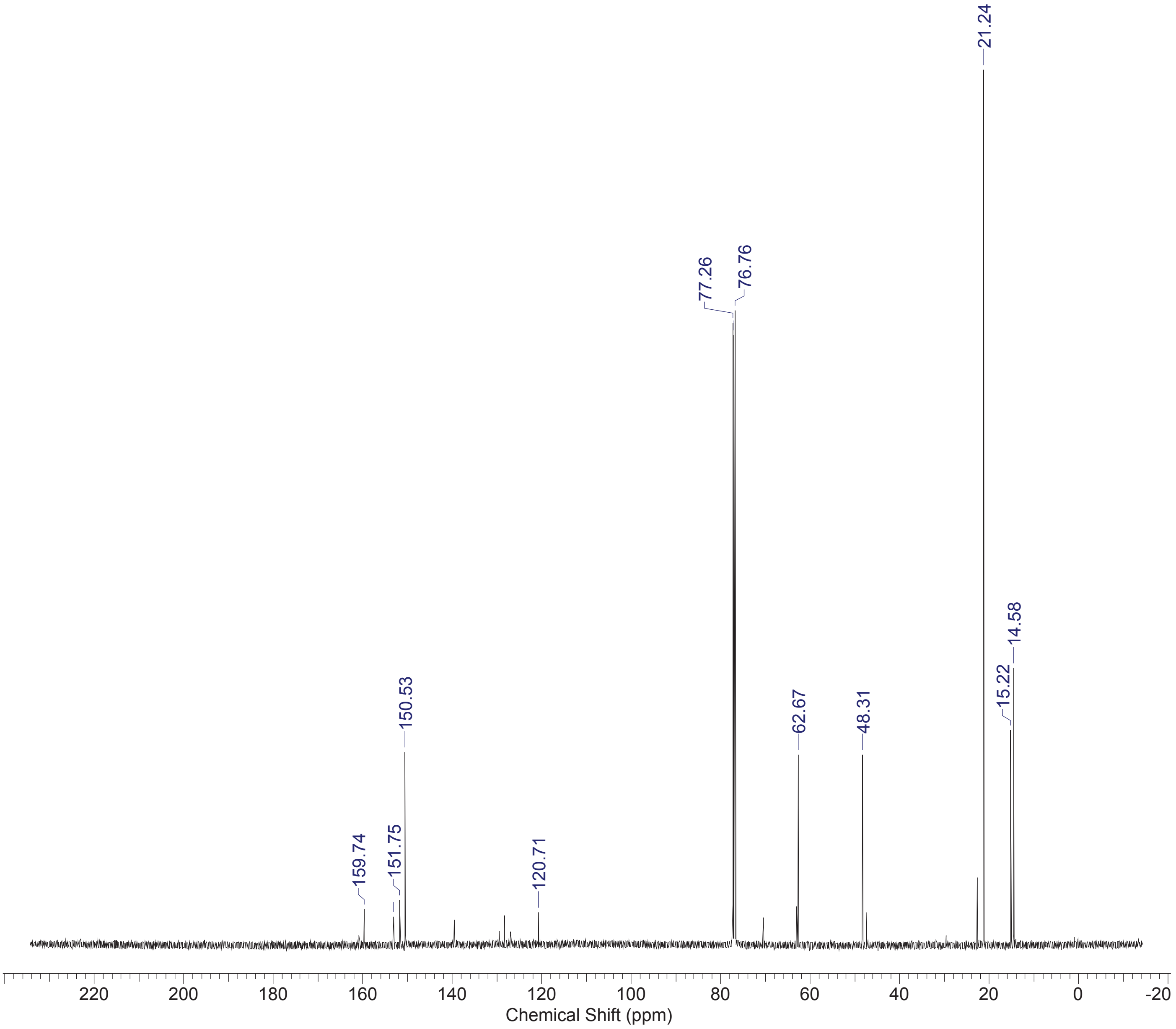

No.	(ppm)	(Hz)	Height
1	14.58	1832.4	0.3162
2	15.22	1912.5	0.2453
3	21.24	2669.7	1.0000
4	48.31	6071.6	0.2170
5	62.67	7877.0	0.2167
6	76.76	9647.0	0.7249
7	77.01	9678.5	0.6964
8	77.26	9710.9	0.7109
9	120.71	15171.8	0.0363
10	150.53	18918.9	0.2196
11	151.75	19072.5	0.0509
12	153.15	19248.9	0.0315
13	159.74	20076.7	0.0403

B6, B7. 6-Ethoxy-9-isopropyl-8-methyl-9H-purine (DEPT)

9 Jul 2012

Acquisition Time (sec)	1.0486	***Date***	Jun 8 2012		
File Name	C:\Users\usuario\Documents\Espectros Asier\12-11090_ASIMJ-3NP\DEPT_01				
Frequency (MHz)	125.68	***Nucleus***	13C	***Number of Transients***	4800
Original Points Count	32768	***Points Count***	32768	***Pulse Sequence***	DEPT135
Solvent	CHLOROFORM-D			***Sweep Width (Hz)***	31250.00
Temperature (degree C)	25.000				

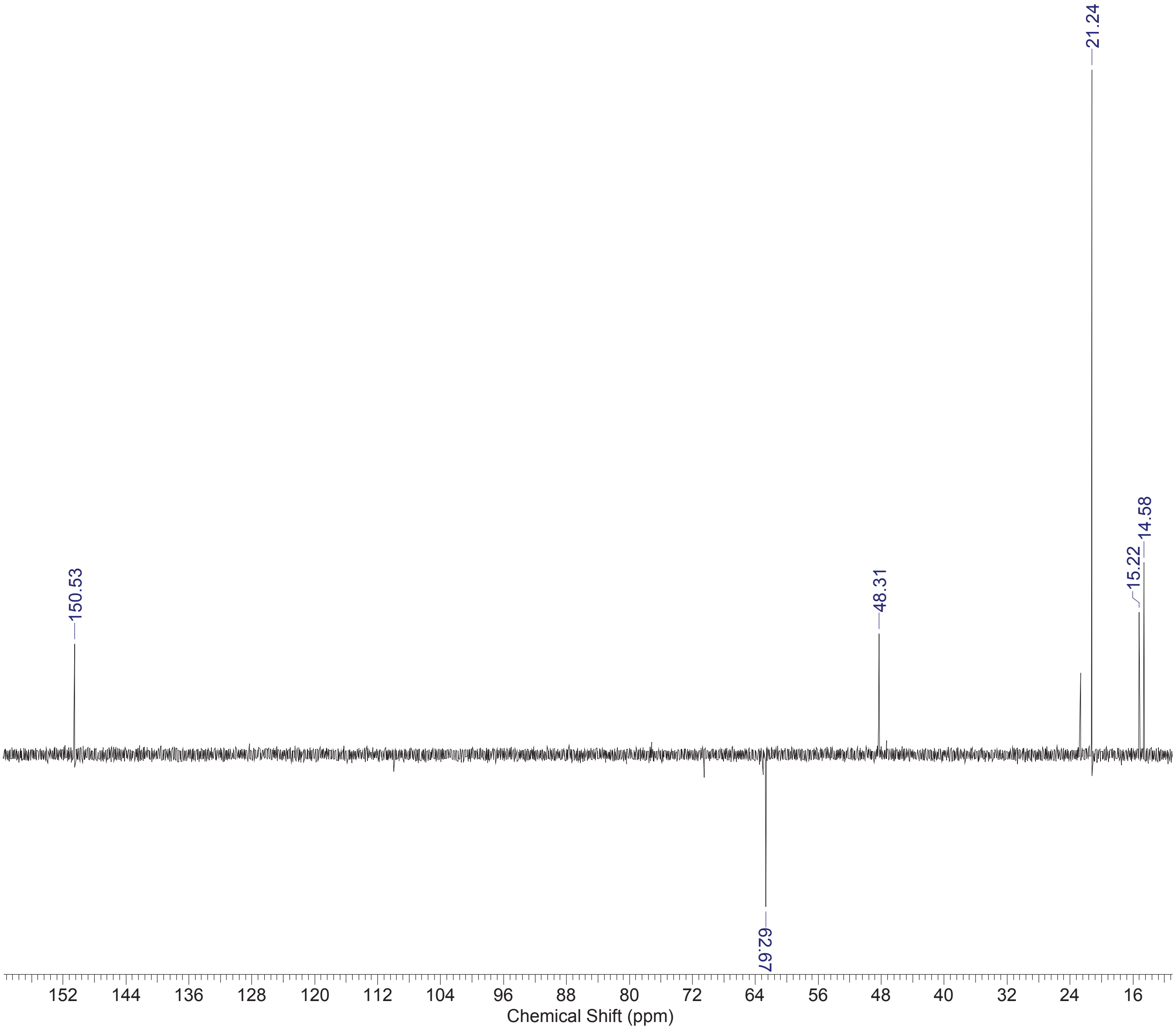

No.	(ppm)	(Hz)	Height
1	14.58	1832.4	0.2804
2	15.22	1913.4	0.2078
3	21.24	2669.7	1.0000
4	48.31	6071.6	0.1761
5	62.67	7877.0	-0.2224
6	150.53	18918.9	0.1615

B6. 6-Ethoxy-9-isopropyl-8-methyl-9H-purine (MS) with propionamide

Elemental Composition Report

Single Mass Analysis

Tolerance = 5.0 PPM / DBE: min = -1.5, max = 50.0
Element prediction: Off
Number of isotope peaks used for i-FIT = 3

Monoisotopic Mass, Even Electron Ions
279 formula(e) evaluated with 1 results within limits (up to 50 closest results for each mass)
Elements Used:
C: 0-20 H: 0-1000 N: 0-6 O: 0-7 Na: 0-1

12/11078

MJ-3NP 6 (0.141)

1: TOF MS ES+
1.31e+003

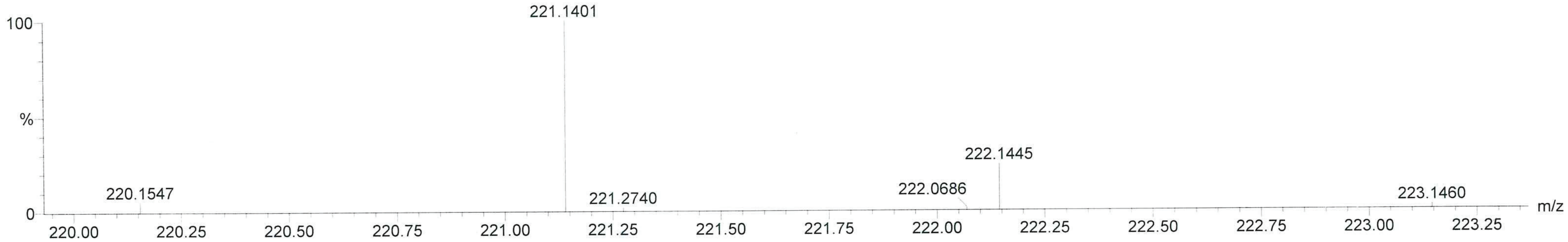

Minimum:				-1.5		
Maximum:		20.0	5.0	50.0		
Mass	Calc. Mass	mDa	PPM	DBE	i-FIT	Formula
221.1401	221.1402	-0.1	-0.5	5.5	29.2	C11 H17 N4 O

B7. 6-Ethoxy-9-isopropyl-8-methyl-9H-purine (MS)(with benzamide)

Elemental Composition Report

Single Mass Analysis

Tolerance = 5.0 PPM / DBE: min = -1.5, max = 50.0
Element prediction: Off
Number of isotope peaks used for i-FIT = 3

Monoisotopic Mass, Even Electron Ions
398 formula(e) evaluated with 2 results within limits (up to 50 best isotopic matches for each mass)
Elements Used:
C: 0-22 H: 0-1000 N: 0-5 O: 0-6 F: 0-3

12/3291

ASIMJ-3 7 (0.163)

1: TOF MS ES+
4.26e+002

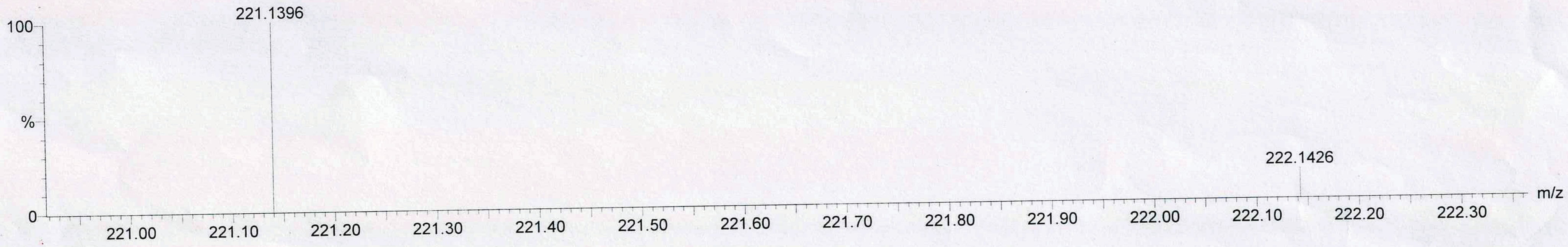

Minimum:				-1.5		
Maximum:		20.0	5.0	50.0		
Mass	Calc. Mass	mDa	PPM	DBE	i-FIT	Formula
221.1396	221.1402	-0.6	-2.7	5.5	n/a	C11 H17 N4 O
	221.1389	0.7	3.2	0.5	n/a	C10 H21 O5

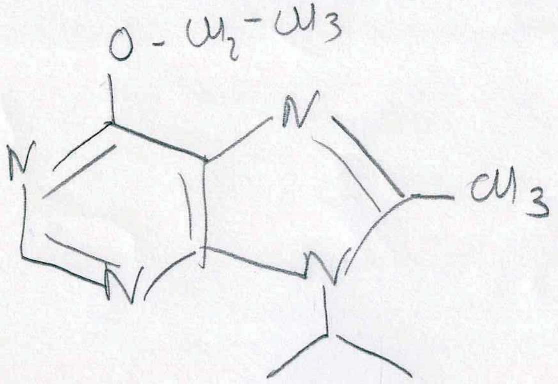

B8. 8-Ethyl-9-isopropyl-6-propoxy-9H-purine (H-NMR)

8 Jul 2013

Acquisition Time (sec)	2.0487	***Comment***	MJ-A2	***Date***	Sep 20 2012
File Name	C:\Users\usuario\Documents\Espectros Asier\1H-MJ-A2				
Frequency (MHz)	300.20	***Nucleus***	1H	***Number of Transients***	64
Original Points Count	7380	***Points Count***	8192	***Pulse Sequence***	s2pul
Solvent	CHLOROFORM-D			***Sweep Width (Hz)***	3602.31
Temperature (degree C)	30.000				

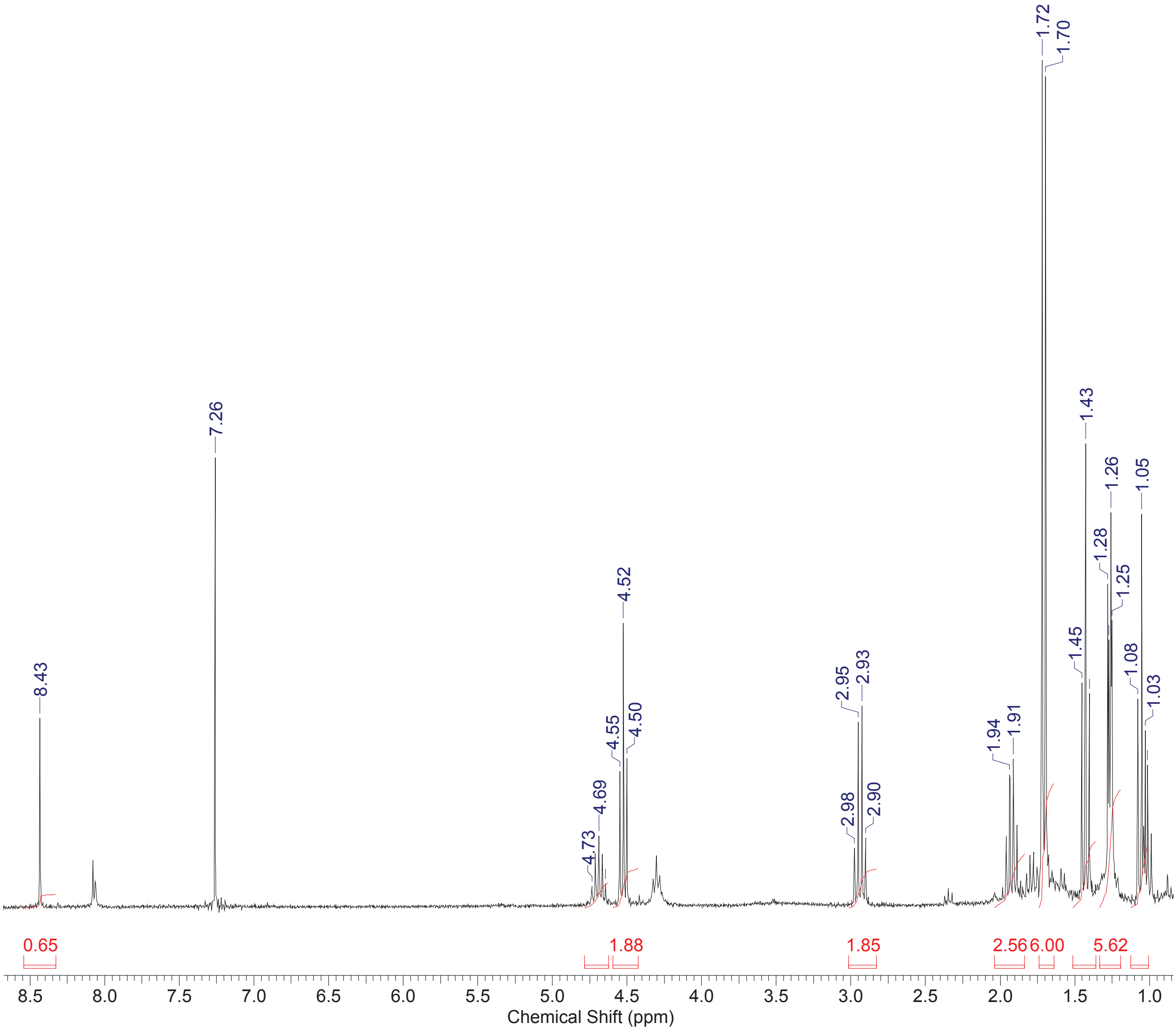

No.	(ppm)	(Hz)	Height
1	1.01	303.8	0.1688
2	1.03	308.2	0.2095
3	1.05	315.6	0.4648
4	1.08	323.1	0.2467
5	1.25	375.0	0.3390
6	1.26	377.6	0.4661
7	1.27	381.6	0.3163
8	1.28	384.2	0.3821
9	1.40	421.2	0.2530
10	1.43	428.7	0.5473
11	1.45	436.1	0.2653
12	1.70	509.1	0.9809
13	1.72	515.7	1.0000
14	1.91	573.8	0.1757

No.	(ppm)	(Hz)	Height
15	1.94	581.3	0.1573
16	2.90	871.1	0.0824
17	2.93	878.6	0.2381
18	2.95	886.0	0.2196
19	2.98	893.9	0.0706
20	4.50	1351.3	0.1761
21	4.52	1358.4	0.3361
22	4.55	1365.4	0.1610
23	4.64	1394.0	0.0288
24	4.69	1407.6	0.0850
25	4.73	1421.3	0.0257
26	7.26	2179.5	0.5310
27	8.43	2531.7	0.2238

No.	(ppm)	Value	Absolute Value
1	[1.01 .. 1.13]	3.126	7.11969e+7
2	[1.19 .. 1.33]	5.618	1.27962e+8
3	[1.36 .. 1.51]	3.214	7.32059e+7
4	[1.64 .. 1.74]	6.000	1.36670e+8
5	[1.84 .. 2.04]	2.559	5.82966e+7
6	[2.83 .. 3.01]	1.852	4.21822e+7
7	[4.43 .. 4.59]	1.879	4.27982e+7
8	[4.62 .. 4.78]	1.198	2.72945e+7
9	[8.33 .. 8.54]	0.645	1.47017e+7

B8. 8-Ethyl-9-isopropyl-6-propoxy-9H-purine (C-NMR)

2 Jul 2013

**Acquisition Time (sec)**	1.3005	**Comment**	MJ-A2	**Date**	Sep 20 2012
**File Name**	C:\Users\usuario\Documents\Espectros Asier\12-20186_MJ-A2\13C-MJ-A2				
**Frequency (MHz)**	75.49	**Nucleus**	13C	**Number of Transients**	16000
**Original Points Count**	23559	**Points Count**	32768	**Pulse Sequence**	s2pul
**Solvent**	CHLOROFORM-D			**Sweep Width (Hz)**	18115.94
**Temperature (degree C)**	30.000				

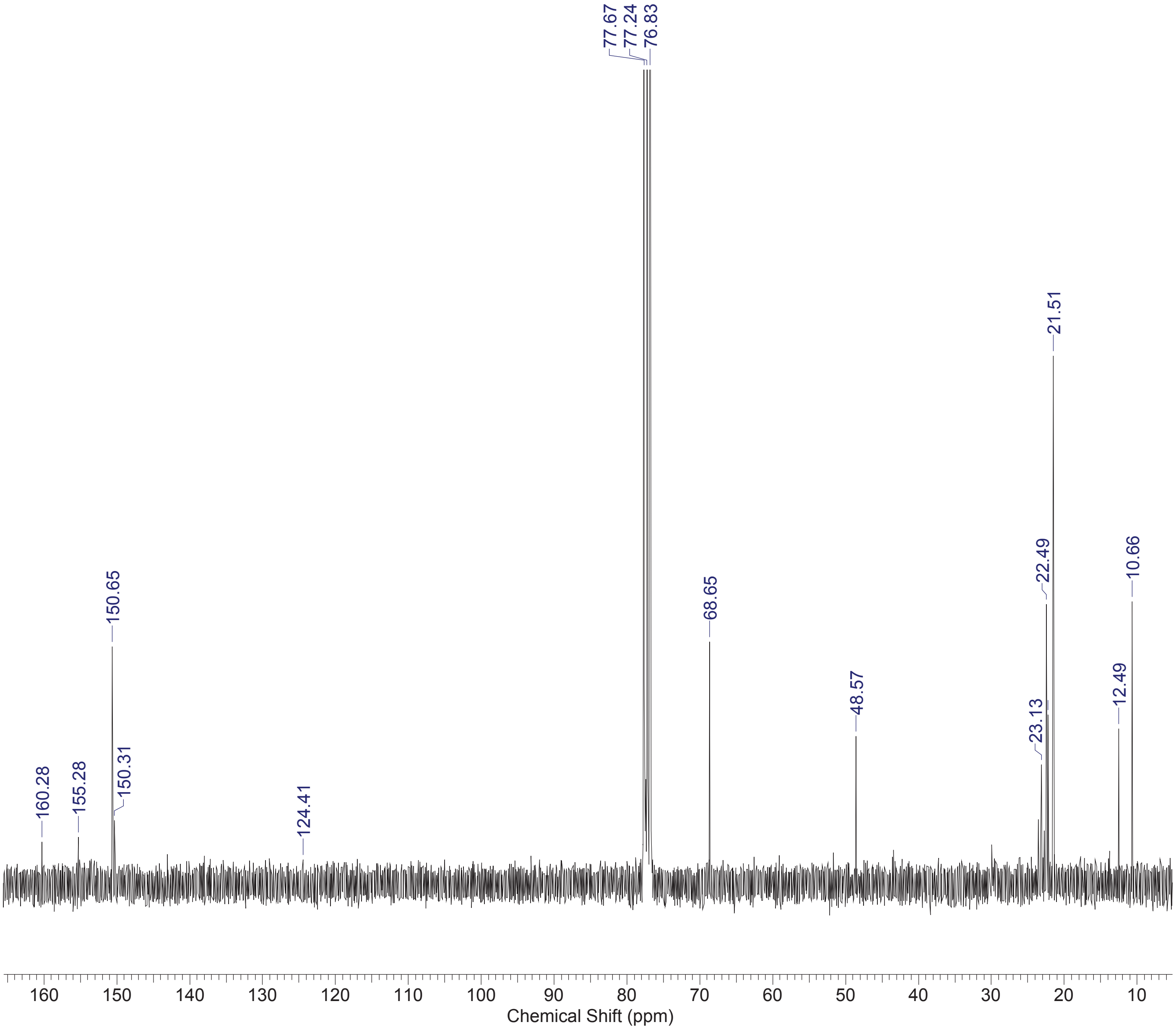

No.	(ppm)	(Hz)	Height
1	10.66	804.6	0.1251
2	12.49	942.9	0.0691
3	21.51	1624.0	0.2337
4	22.20	1676.0	0.0753
5	22.49	1697.5	0.1240
6	23.13	1746.2	0.0532
7	48.57	3666.9	0.0657
8	68.65	5182.8	0.1075
9	76.83	5799.8	0.9759
10	77.24	5831.4	0.9895
11	77.67	5863.4	1.0000
12	124.41	9391.8	0.0113
13	150.31	11347.4	0.0285
14	150.65	11372.8	0.1053
15	155.28	11722.8	0.0213
16	160.28	12099.8	0.0190

B8. 8-Ethyl-9-isopropyl-6-propoxy-9H-purine (MS)

Elemental Composition Report

Single Mass Analysis
Tolerance = 5.0 PPM / DBE: min = -1.5, max = 50.0
Element prediction: Off
Number of isotope peaks used for i-FIT = 3

Monoisotopic Mass, Even Electron Ions
933 formula(e) evaluated with 1 results within limits (up to 50 closest results for each mass)
Elements Used:
C: 0-20 H: 0-1000 N: 0-10 O: 0-20 S: 0-1 Cl: 0-1

12/20093

A3 72 (1.585)

1: TOF MS ES+
6.28e+002

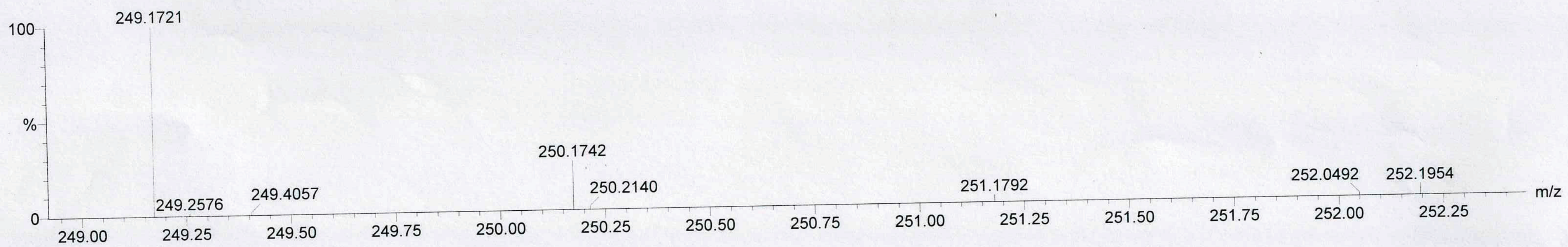

Minimum:				-1.5		
Maximum:		5.0	5.0	50.0		
Mass	Calc. Mass	mDa	PPM	DBE	i-FIT	Formula
249.1721	249.1715	0.6	2.4	5.5	13.5	C13 H21 N4 O

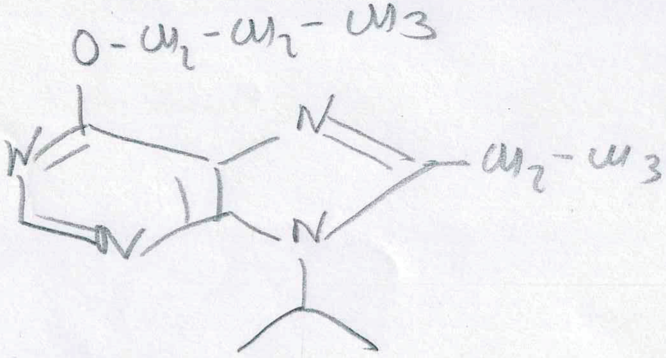

B9. 6-Isobutoxy-8,9-diisopropyl-9H-purine (H-NMR)

2 Jul 2013

Acquisition Time (sec)	2.0487	***Comment***	ASIMJ-28	***Date***	Nov 8 2012
File Name	C:\Users\usuario\Documents\Espectros Asier\12-22927_ASIMJ-28\1H-ASIMJ-28				
Frequency (MHz)	300.20	***Nucleus***	1H	***Number of Transients***	64
Original Points Count	7380	***Points Count***	8192	***Pulse Sequence***	s2pul
Solvent	CHLOROFORM-D			***Sweep Width (Hz)***	3602.31
Temperature (degree C)	30.000				

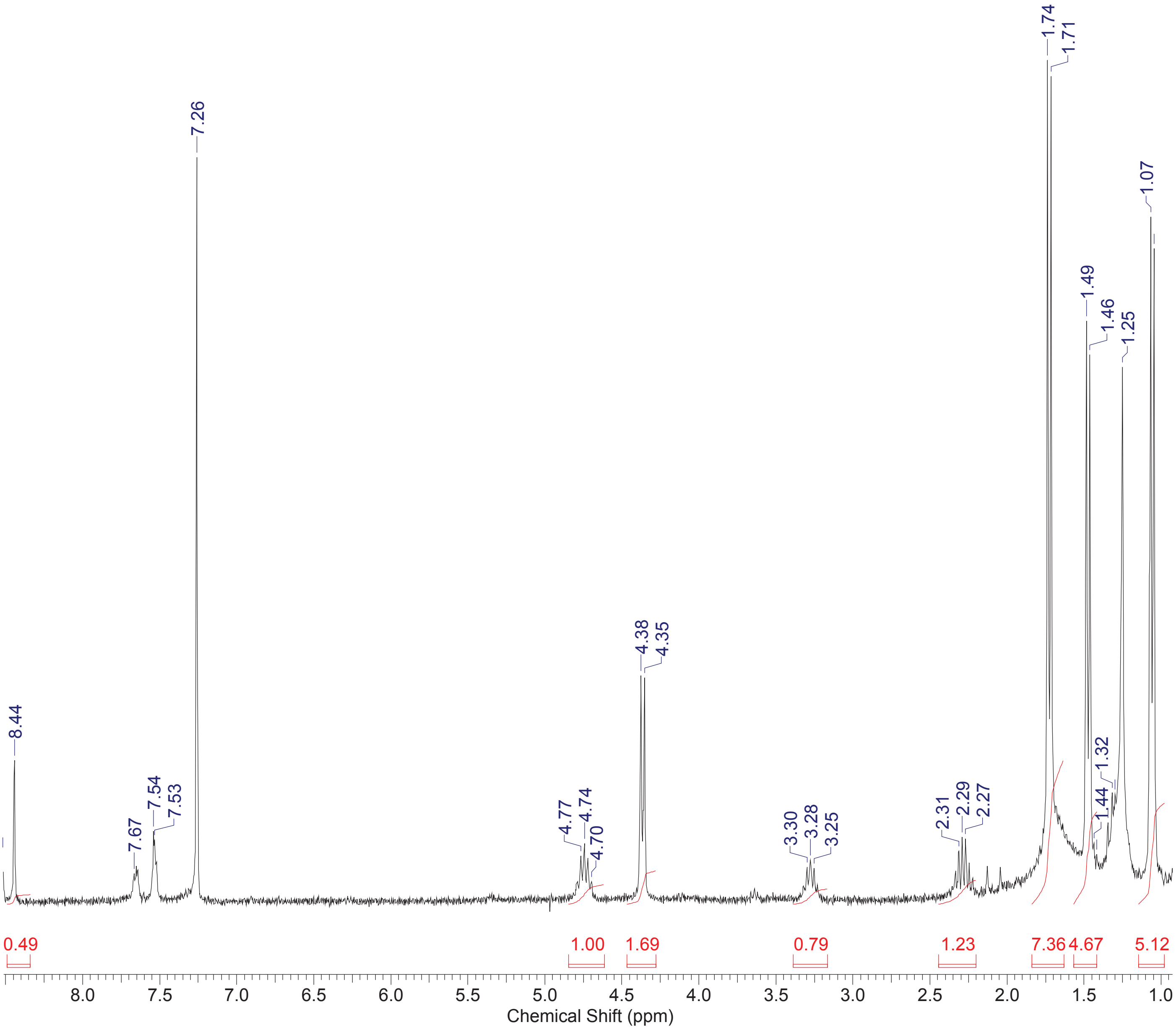

No.	(ppm)	(Hz)	Height
1	0.90	270.8	0.0616
2	1.05	313.9	0.7765
3	1.07	320.5	0.8141
4	1.25	375.9	0.6365
5	1.30	390.4	0.1307
6	1.32	395.2	0.1323
7	1.35	404.0	0.0966
8	1.42	426.0	0.0595
9	1.44	431.3	0.0728
10	1.46	438.8	0.6506
11	1.49	445.8	0.6911
12	1.71	514.4	0.9806
13	1.74	521.4	1.0000
14	2.27	681.1	0.0776
15	2.29	687.7	0.0800

No.	(ppm)	(Hz)	Height
16	2.31	694.3	0.0634
17	3.25	976.6	0.0428
18	3.28	983.7	0.0524
19	3.30	990.3	0.0439
20	4.35	1306.5	0.2686
21	4.38	1313.5	0.2709
22	4.70	1410.3	0.0271
23	4.74	1423.9	0.0719
24	4.77	1430.5	0.0572
25	7.26	2179.5	0.8846
26	7.53	2261.3	0.0765
27	7.54	2263.9	0.0865
28	7.67	2301.3	0.0365
29	8.44	2534.8	0.1705
30	8.52	2557.2	0.0618

No.	(ppm)	Value	Absolute Value
1	[0.98 .. 1.15]	5.125	3.00038e+8
2	[1.42 .. 1.57]	4.671	2.73462e+8
3	[1.63 .. 1.84]	7.357	4.30721e+8
4	[2.20 .. 2.44]	1.231	7.20844e+7
5	[3.16 .. 3.39]	0.786	4.60005e+7
6	[4.28 .. 4.47]	1.692	9.90542e+7
7	[4.61 .. 4.85]	1.000	5.85494e+7
8	[8.34 .. 8.49]	0.490	2.86777e+7

B9. 6-Isobutoxy-8,9-diisopropyl-9H-purine (MS)

Elemental Composition Report

Single Mass Analysis

Tolerance = 5.0 PPM / DBE: min = -1.5, max = 50.0
Element prediction: Off
Number of isotope peaks used for i-FIT = 3

Monoisotopic Mass, Even Electron Ions
910 formula(e) evaluated with 2 results within limits (up to 50 closest results for each mass)
Elements Used:
C: 0-23 H: 0-1000 N: 0-10 O: 0-10 Cl: 0-1 Br: 0-1

12/22452

MJ-28 96 (2.120)

1: TOF MS ES+
1.15e+002

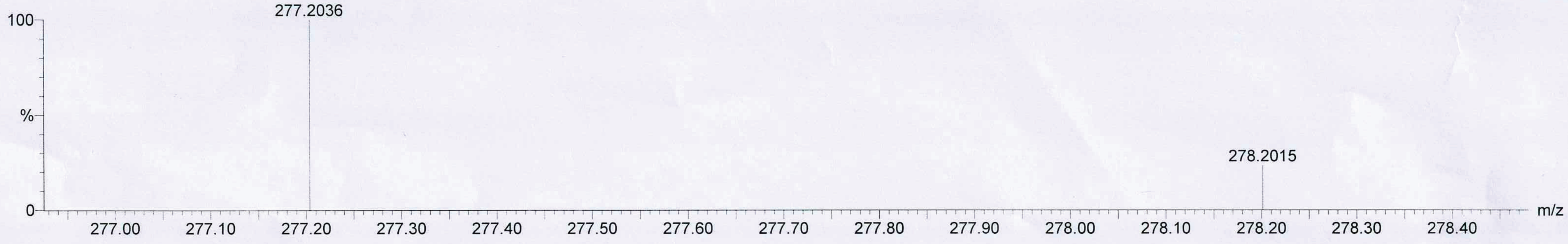

Minimum:				-1.5		
Maximum:		5.0	5.0	50.0		

Mass	Calc. Mass	mDa	PPM	DBE	i-FIT	Formula
277.2036	277.2028	0.8	2.9	5.5	n/a	C15 H25 N4 O
	277.2047	-1.1	-4.0	0.5	n/a	C14 H30 N2 O Cl

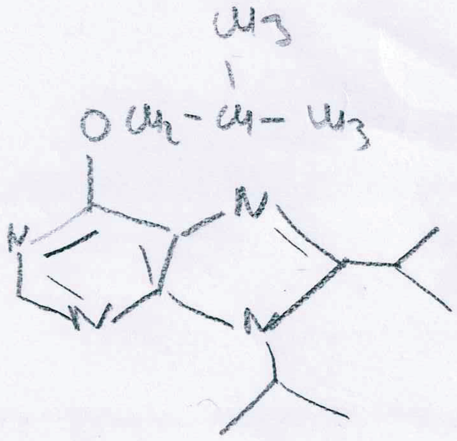

Supplement: Supplementary Information — Supplementary Material [file srep09139-s1.pdf]
